# THE CONCISE GUIDE TO PHARMACOLOGY 2017/18: G protein‐coupled receptors

**DOI:** 10.1111/bph.13878

**Published:** 2017-10-21

**Authors:** Stephen PH Alexander, Arthur Christopoulos, Anthony P Davenport, Eamonn Kelly, Neil V Marrion, John A Peters, Elena Faccenda, Simon D Harding, Adam J Pawson, Joanna L Sharman, Christopher Southan, Jamie A Davies, CGTP Collaborators

**Affiliations:** ^1^ School of Life Sciences University of Nottingham Medical School Nottingham NG7 2UH UK; ^2^ Monash Institute of Pharmaceutical Sciences and Department of Pharmacology Monash University Parkville, Victoria 3052 Australia; ^3^ Clinical Pharmacology Unit University of Cambridge Cambridge CB2 0QQ UK; ^4^ School of Physiology, Pharmacology and Neuroscience University of Bristol Bristol BS8 1TD UK; ^5^ Neuroscience Division, Medical Education Institute, Ninewells Hospital and Medical School University of Dundee Dundee DD1 9SY UK; ^6^ Centre for Integrative Physiology University of Edinburgh Edinburgh EH8 9XD UK

## Abstract

The Concise Guide to PHARMACOLOGY 2017/18 provides concise overviews of the key properties of nearly 1800 human drug targets with an emphasis on selective pharmacology (where available), plus links to an open access knowledgebase of drug targets and their ligands (www.guidetopharmacology.org), which provides more detailed views of target and ligand properties. Although the Concise Guide represents approximately 400 pages, the material presented is substantially reduced compared to information and links presented on the website. It provides a permanent, citable, point‐in‐time record that will survive database updates. The full contents of this section can be found at http://onlinelibrary.wiley.com/doi/10.1111/bph.13878/full. G protein‐coupled receptors are one of the eight major pharmacological targets into which the Guide is divided, with the others being: ligand‐gated ion channels, voltage‐gated ion channels, other ion channels, nuclear hormone receptors, catalytic receptors, enzymes and transporters. These are presented with nomenclature guidance and summary information on the best available pharmacological tools, alongside key references and suggestions for further reading. The landscape format of the Concise Guide is designed to facilitate comparison of related targets from material contemporary to mid‐2017, and supersedes data presented in the 2015/16 and 2013/14 Concise Guides and previous Guides to Receptors and Channels. It is produced in close conjunction with the Nomenclature Committee of the Union of Basic and Clinical Pharmacology (NC‐IUPHAR), therefore, providing official IUPHAR classification and nomenclature for human drug targets, where appropriate.

1

### Conflict of interest

The authors state that there are no conflicts of interest to declare.

### Overview

G protein‐coupled receptors (GPCRs) are the largest class of membrane proteins in the human genome. The term "7TM receptor" is commonly used interchangeably with "GPCR", although there are some receptors with seven transmembrane domains that do not signal through G proteins. GPCRs share a common architecture, each consisting of a single polypeptide with an extracellular N‐terminus, an intracellular C‐terminus and seven hydrophobic transmembrane domains (TM1‐TM7) linked by three extracellular loops (ECL1‐ECL3) and three intracellular loops (ICL1‐ICL3). About 800 GPCRs have been identified in man, of which about half have sensory functions, mediating olfaction (∼400), taste (33), light perception (10) and pheromone signalling (5) [1362]. The remaining ∼350 non‐sensory GPCRs mediate signalling by ligands that range in size from small molecules to peptides to large proteins; they are the targets for the majority of drugs in clinical usage [1519, 1631], although only a minority of these receptors are exploited therapeutically. The first classification scheme to be proposed for GPCRs [1030] divided them, on the basic of sequence homology, into six classes. These classes and their prototype members were as follows: Class A (rhodopsin‐like), Class B (secretin receptor family), Class C (metabotropic glutamate), Class D (fungal mating pheromone receptors), Class E (cyclic AMP receptors) and Class F (frizzled/smoothened). Of these, classes D and E are not found in vertebrates. An alternative classification scheme "GRAFS" [1737] divides vertebrate GPCRs into five classes, overlapping with the A‐F nomenclature, viz:

Glutamate family (class C), which includes metabotropic glutamate receptors, a calcium‐sensing receptor and GABA_B_ receptors, as well as three taste type 1 receptors and a family of pheromone receptors (V2 receptors) that are abundant in rodents but absent in man [1362].

Rhodopsin family (class A), which includes receptors for a wide variety of small molecules, neurotransmitters, peptides and hormones, together with olfactory receptors, visual pigments, taste type 2 receptors and five pheromone receptors (V1 receptors).

Adhesion family GPCRs are phylogenetically related to class B receptors, from which they differ by possessing large extracellular N‐termini that are autoproteolytically cleaved from their 7TM domains at a conserved "GPCR proteolysis site" (GPS) which lies within a much larger (˜ 320 residue) "GPCR autoproteolysis‐inducing" (GAIN) domain, an evolutionary ancient mofif also found in polycystic kidney disease 1 (PKD1)‐like proteins, which has been suggested to be both required and sufficient for autoproteolysis [1609].

Frizzled family consists of 10 Frizzled proteins (FZD(1‐10)) and Smoothened (SMO). The FZDs are activated by secreted lipoglycoproteins of the WNT family, whereas SMO is indirectly activated by the Hedgehog (HH) family of proteins acting on the transmembrane protein Patched (PTCH).

Secretin family (class B), encoded by 15 genes in humans. The ligands for receptors in this family are polypeptide hormones of 27‐141 amino acid residues; nine of the mammalian receptors respond to ligands that are structurally related to one another (glucagon, glucagon‐like peptides (GLP‐1, GLP‐2), glucose‐dependent insulinotropic polypeptide (GIP), secretin, vasoactive intestinal peptide (VIP), pituitary adenylate cyclase‐activating polypeptide (PACAP) and growth‐hormone‐releasing hormone (GHRH)) [738].

### GPCR families

**Table:** 

Family	Class A	Class B (Secretin)	Class C (Glutamate)	Adhesion	Frizzled
Receptors with known ligands	197	15	12	0	11
Orphans	87 (54)^a^	‐	8 (1)^a^	26 (6)^a^	0
Sensory (olfaction)	390^b,c^	‐	‐	‐	‐
Sensory (vision)	10^d^ opsins	‐	‐	‐	‐
Sensory (taste)	30^c^ taste 2	‐	3^c^ taste 1	‐	‐
Sensory (pheromone)	5^c^ vomeronasal 1	‐	‐	‐	‐
Total	719	15	22	33	11

^a^Numbers in brackets refer to orphan receptors for which an endogenous ligand has been proposed in at least one publication, see [414]; ^b^[1511]; ^c^[1362]; ^d^[1941].

Much of our current understanding of the structure and function of GPCRs is the result of pioneering work on the visual pigment rhodopsin and on the *β*_2_ adrenoceptor, the latter culminating in the award of the 2012 Nobel Prize in chemistry to Robert Lefkowitz and Brian Kobilka [1021, 1137].

### Family structure

S19 Orphan and other 7TM receptors

S19 Class A Orphans

Ű Class B Orphans

S28 Class C Orphans

S28 Taste 1 receptors

S29 Taste 2 receptors

S30 Other 7TM proteins

S30 5‐Hydroxytryptamine receptors

S34 Acetylcholine receptors (muscarinic)

S36 Adenosine receptors

S37 Adhesion Class GPCRs

S39 Adrenoceptors

S43 Angiotensin receptors

S44 Apelin receptor

S45 Bile acid receptor

S45 Bombesin receptors

S47 Bradykinin receptors

S48 Calcitonin receptors

S50 Calcium‐sensing receptor

S51 Cannabinoid receptors

S52 Chemerin receptor

S52 Chemokine receptors

S57 Cholecystokinin receptors

S58 Class Frizzled GPCRs

S59 Complement peptide receptors

S60 Corticotropin‐releasing factor receptors

S61 Dopamine receptors

S63 Endothelin receptors

S64 G protein‐coupled estrogen receptor

S65 Formylpeptide receptors

S66 Free fatty acid receptors

S67 GABAB receptors

S69 Galanin receptors

S70 Ghrelin receptor

S71 Glucagon receptor family

S72 Glycoprotein hormone receptors

S73 Gonadotrophin‐releasing hormone receptors

S74 GPR18, GPR55 and GPR119

S76 Histamine receptors

S77 Hydroxycarboxylic acid receptors

S78 Kisspeptin receptor

S79 Leukotriene receptors

S80 Lysophospholipid (LPA) receptors

S82 Lysophospholipid (S1P) receptors

S83 Melanin‐concentrating hormone receptors

S84 Melanocortin receptors

S85 Melatonin receptors

S86 Metabotropic glutamate receptors

S88 Motilin receptor

S89 Neuromedin U receptors

S90 Neuropeptide FF/neuropeptide AF receptors

S91 Neuropeptide S receptor

S91 Neuropeptide W/neuropeptide B receptors

S92 Neuropeptide Y receptors

S94 Neurotensin receptors

S95 Opioid receptors

S97 Orexin receptors

S98 Oxoglutarate receptor

S98 P2Y receptors

S100 Parathyroid hormone receptors

S101 Platelet‐activating factor receptor

S102 Prokineticin receptors

S103 Prolactin‐releasing peptide receptor

S104 Prostanoid receptors

S106 Proteinase‐activated receptors

S107 QRFP receptor

S108 Relaxin family peptide receptors

S110 Somatostatin receptors

S110 Succinate receptor

S111 Tachykinin receptors

S112 Thyrotropin‐releasing hormone receptors

S113 Trace amine receptor

S114 Urotensin receptor

S115 Vasopressin and oxytocin receptors

S116 VIP and PACAP receptors

## Orphan and other 7TM receptors

## Class A Orphans

### Overview

Table 1 lists a number of putative GPCRs identified by NC‐IUPHAR[557], for which preliminary evidence for an endogenous ligand has been published, or for which there exists a potential link to a disease, or disorder. These GPCRs have recently been reviewed in detail [414]. The GPCRs in Table 1 are all Class A, rhodopsin‐like GPCRs. Class A orphan GPCRs not listed in Table 1 are putative GPCRs with as‐yet unidentified endogenous ligands.

### Table 1

Class A orphan GPCRs with putative endogenous ligands

**Table:** 

GPR1	GPR3	GPR4	GPR6	GPR12	GPR15	GPR17	GPR20
GPR22	GPR26	GPR31	GPR34	GPR35	GPR37	GPR39	GPR50
GPR63	GRP65	GPR68	GPR75	GPR84	GPR87	GPR88	GPR132
GPR149	GPR161	GPR183	LGR4	LGR5	LGR6	MAS1	MRGPRD
MRGPRX1	MRGPRX2	P2RY10	TAAR2				

In addition the orphan receptors GPR18, GPR55 and GPR119 which are reported to respond to endogenous agents analogous to the endogenous cannabinoid ligands have been grouped together (GPR18, GPR55 and GPR119).

**Table:** 

Nomenclature	GPR1	GPR3
HGNC, UniProt	GPR1, P46091	GPR3, P46089
Endogenous agonists	chemerin (RARRES2, Q99969) [101]	–
Agonists	–	diphenyleneiodonium chloride [2179]
Comments	Reported to act as a co‐receptor for HIV [1791]. See review [414] for discussion of pairing with chemerin.	sphingosine 1‐phosphate was reported to be an endogenous agonist [1997], but this finding was not replicated in subsequent studies [2182]. Reported to activate adenylyl cyclase constitutively through G_s_ [494]. Gene disruption results in premature ovarian ageing [1128], reduced *β*‐amyloid deposition [1943] and hypersensitivity to thermal pain [1689] in mice. First small molecule inverse agonist [903] and agonists identified [2179].

**Table:** 

Nomenclature	GPR4	GPR6	GPR42
HGNC, UniProt	GPR4, P46093	GPR6, P46095	GPR42, O15529
Endogenous ligands	Protons	–	–
Comments	An initial report suggesting activation by lysophosphatidylcholine and sphingosylphosphorylcholine [2225] has been retracted [1470]. GPR4, GPR65, GPR68 and GPR132 are now thought to function as proton‐sensing receptors detecting acidic pH [414, 1775]. Gene disruption is associated with increased perinatal mortality and impaired vascular proliferation [2173]. Negative allosteric modulators of GPR4 have been reported [1967].	An initial report that sphingosine 1‐phosphate (S1P) was a high‐affinity ligand (EC_50_ value of 39nM) [855, 1997] was not repeated in arrestin‐based assays [1854, 2182]. Reported to activate adenylyl cyclase constitutively through G_s_ and to be located intracellularly [1521]. GPR6‐deficient mice showed reduced striatal cyclic AMP production in vitro and selected alterations in instrumental conditioning in vivo. [1200].	–

**Table:** 

Nomenclature	GPR12	GPR15	GPR17
HGNC, UniProt	GPR12, P47775	GPR15, P49685	GPR17, Q13304
Endogenous agonists	–	–	UDP‐glucose [134, 359], LTC_4_ [359], UDP‐galactose [134, 359], uridine diphosphate [134, 359], LTD_4_ [359]
Comments	Reports that sphingosine 1‐phosphate is a ligand of GPR12 [854, 1997] have not been replicated in arrestin‐based assays [1854, 2182]. Gene disruption results in dyslipidemia and obesity [158].	Reported to act as a co‐receptor for HIV [490]. In an infection‐induced colitis model, Gpr15 knockout mice were more prone to tissue damage and inflammatory cytokine expression [991].	Reported to be a dual leukotriene and uridine diphosphate receptor [359]. Another group instead proposed that GPR17 functions as a negative regulator of the CysLT_1_ receptor response to leukotriene D_4_ (LTD_4_). For further discussion, see [414]. Reported to antagonize CysLT_1_ receptor signalling in vivo and in vitro [1239]. See reviews [258] and [414].

**Table:** 

Nomenclature	GPR19	GPR20	GPR21	GPR22	GPR25	GPR26	GPR27
HGNC, UniProt	GPR19, Q15760	GPR20, Q99678	GPR21, Q99679	GPR22, Q99680	GPR25, O00155	GPR26, Q8NDV2	GPR27, Q9NS67
Comments	–	Reported to inhibit adenylyl cyclase constitutively through G_i/o_ [743]. GPR20 deficient mice exhibit hyperactivity characterised by increased total distance travelled in an open field test [213].	Gpr21 knockout mice were resistant to diet‐induced obesity, exhibiting an increase in glucose tolerance and insulin sensitivity, as well as a modest lean phenotype [1516].	Gene disruption results in increased severity of functional decompensation following aortic banding [10]. Identified as a susceptibility locus for osteoarthritis [520, 975, 2011].	–	Has been reported to activate adenylyl cyclase constitutively through G_s_ [923]. Gpr26 knockout mice show increased levels of anxiety and depression‐like behaviours [2209].	Knockdown of Gpr27 reduces endogenous mouse insulin promotor activity and glucose‐stimulated insulin secretion [1059].

**Table:** 

Nomenclature	GPR31	GPR32	GPR33	GPR34
HGNC, UniProt	GPR31, O00270	GPR32, O75388	GPR33, Q49SQ1	GPR34, Q9UPC5
Potency order of endogenous ligands	–	resolvin D1>LXA_4_	–	–
Endogenous agonists	12S‐HETE [700] – Mouse	resolvin D1 [1052], LXA_4_ [1052]	–	lysophosphatidylserine [1008, 1891]
Labelled ligands	–	[^3^ H]resolvin D1 (Agonist) [1052]	–	–
Comments	See [414] for discussion of pairing.	resolvin D1 has been demonstrated to activate GPR32 in two publications [331, 1052]. The pairing was not replicated in a recent study based on arrestin recruitment [1854]. GPR32 is a pseudogene in mice and rats. See reviews [258] and [414].	GPR33 is a pseudogene in most individuals, containing a premature stop codon within the coding sequence of the second intracellular loop [1696].	Lysophosphatidylserine has been reported to be a ligand of GPR34 in several publications, but the pairing was not replicated in a recent study based on arrestin recruitment [1854]. Fails to respond to a variety of lipid‐derived agents [2182]. Gene disruption results in an enhanced immune response [1168]. Characterization of agonists at this receptor is discussed in [859] and [414].

**Table:** 

Nomenclature	GPR35	GPR37
HGNC, UniProt	GPR35, Q9HC97	GPR37, O15354
Endogenous agonists	2‐oleoyl‐LPA [1503], kynurenic acid [1854, 2066]	–
Agonists	–	neuropeptide head activator [1652]
Comments	Several studies have shown that kynurenic acid is an agonist of GPR35 but it remains controversial whether the proposed endogenous ligand reaches sufficient tissue concentrations to activate the receptor [1061]. 2‐oleoyl‐LPA has also been proposed as an endogenous ligand [1503] but these results were not replicated in an arrestin assay [1854]. The phosphodiesterase inhibitor zaprinast [1937] has become widely used as a surrogate agonist to investigate GPR35 pharmacology and signalling [1937]. GPR35 is also activated by the pharmaceutical adjunct pamoic acid [2218]. See reviews [414] and [453].	Reported to associate and regulate the dopamine transporter [1269] and to be a substrate for parkin [1267]. Gene disruption results in altered striatal signalling [1268]. The peptides prosaptide and prosaposin are proposed as endogenous ligands for GPR37 and GPR37L1 [1324].

**Table:** 

Nomenclature	GPR37L1	GPR39	GPR45	GPR50
HGNC, UniProt	GPR37L1, O60883	GPR39, O43194	GPR45, Q9Y5Y3	GPR50, Q13585
Endogenous agonists	–	Zn ^2+^ [813]	–	–
Comments	The peptides prosaptide and prosaposin are proposed as endogenous ligands for GPR37 and GPR37L1 [1324].	Zn^2+^ has been reported to be a potent and efficacious agonist of human, mouse and rat GPR39 [2176]. obestatin (GHRL, Q9UBU3), a fragment from the ghrelin precursor, was reported initially as an endogenous ligand, but subsequent studies failed to reproduce these findings. GPR39 has been reported to be down‐regulated in adipose tissue in obesity‐related diabetes [285]. Gene disruption results in obesity and altered adipocyte metabolism [1567]. Reviewed in [414].	–	GPR50 is structurally related to MT_1_ and MT_2_ melatonin receptors, with which it heterodimerises constitutively and specifically [1155]. Gpr50 knockout mice display abnormal thermoregulation and are much more likely than wild‐type mice to enter fasting‐induced torpor [117].

**Table:** 

Nomenclature	GPR52	GPR61	GPR62	GPR63	GPR65
HGNC, UniProt	GPR52, Q9Y2T5	GPR61, Q9BZJ8	GPR62, Q9BZJ7	GPR63, Q9BZJ6	GPR65, Q8IYL9
Endogenous ligands	–	–	–	–	Protons
Comments	First small molecule agonist reported [1774].	GPR61 deficient mice exhibit obesity associated with hyperphagia [1422]. Although no endogenous ligands have been identified, 5‐(nonyloxy)tryptamine has been reported to be a low affinity inverse agonist [1925].	–	sphingosine 1‐phosphate and dioleoylphosphatidic acid have been reported to be low affinity agonists for GPR63 [1459] but this finding was not replicated in an arrestin‐based assay [2182].	GPR4, GPR65, GPR68 and GPR132 are now thought to function as proton‐sensing receptors detecting acidic pH [414, 1775]. Reported to activate adenylyl cyclase; gene disruption leads to reduced eosinophilia in models of allergic airway disease [1044].

**Table:** 

Nomenclature	GPR68	GPR75	GPR78	GPR79	GPR82
HGNC, UniProt	GPR68, Q15743	GPR75, O95800	GPR78, Q96P69	GPR79, –	GPR82, Q96P67
Endogenous ligands	Protons	–	–	–	–
Allosteric modulators	lorazepam (Positive) [838]	–	–	–	–
Comments	GPR68 was previously identified as a receptor for sphingosylphosphorylcholine (SPC) [2157], but the original publication has been retracted [2156]. GPR4, GPR65, GPR68 and GPR132 are now thought to function as proton‐sensing receptors detecting acidic pH [414, 1775]. A family of 3,5‐disubstituted isoxazoles were identified as agonists of GPR68 [1691].	CCL5 (CCL5, P13501) was reported to be an agonist of GPR75 [856], but the pairing could not be repeated in an arrestin assay [1854].	GPR78 has been reported to be constitutively active, coupled to elevated cAMP production [923].	–	Mice with Gpr82 knockout have a lower body weight and body fat content associated with reduced food intake, decreased serum triglyceride levels, as well as higher insulin sensitivity and glucose tolerance [507].

**Table:** 

Nomenclature	GPR83	GPR84	GPR85	GPR87	GPR88	GPR101
HGNC, UniProt	GPR83, Q9NYM4	GPR84, Q9NQS5	GPR85, P60893	GPR87, Q9BY21	GPR88, Q9GZN0	GPR101, Q96P66
Endogenous agonists	–	–	–	LPA [1401, 1911]	–	–
Agonists	PEN {Mouse} [655] – Mouse, Zn ^2+^ [1409] – Mouse	decanoic acid [1854, 2067], undecanoic acid [2067], lauric acid [2067]	–	–		–
Comments	One isoform has been implicated in the induction of CD4(+) CD25(+) regulatory T cells (Tregs) during inflammatory immune responses [731]. The extracellular N‐terminal domain is reported as an intramolecular inverse agonist [1410].	Medium chain free fatty acids with carbon chain lengths of 9‐14 activate GPR84 [1901, 2067]. A surrogate ligand for GPR84, 6‐n‐octylaminouracil has also been proposed [1901]. See review [414] for discussion of classification. Mutational analysis and molecular modelling of GPR84 has been reported [1463].	Proposed to regulate hippocampal neurogenesis in the adult, as well as neurogenesis‐dependent learning and memory [319].	–	Gene disruption results in altered striatal signalling [1203]. Small molecule agonists have been reported [151].	Mutations in GPR101 have been linked to gigantism and acromegaly [1982].

**Table:** 

Nomenclature	GPR132	GPR135	GPR139	GPR141	GPR142	GPR146
HGNC, UniProt	GPR132, Q9UNW8	GPR135, Q8IZ08	GPR139, Q6DWJ6	GPR141, Q7Z602	GPR142, Q7Z601	GPR146, Q96CH1
Endogenous ligands	Protons	–	–	–	–	–
Comments	GPR4, GPR65, GPR68 and GPR132 are now thought to function as proton‐sensing receptors detecting acidic pH [414, 1775]. Reported to respond to lysophosphatidylcholine [934], but later retracted [2126].	–	Peptide agonists have been reported [867].	–	Small molecule agonists have been reported [1968, 2196].	Yosten et al. demonstrated inhibition of proinsulin C‐peptide (INS, P01308)‐induced stimulation of cFos expression folllowing knockdown of GPR146 in KATO III cells, suggesting proinsulin C‐peptide as an endogenous ligand of the receptor [2193].

**Table:** 

Nomenclature	GPR148	GPR149	GPR150	GPR151	GPR152	GPR153	GPR160
HGNC, UniProt	GPR148, Q8TDV2	GPR149, Q86SP6	GPR150, Q8NGU9	GPR151, Q8TDV0	GPR152, Q8TDT2	GPR153, Q6NV75	GPR160, Q9UJ42
Comments	–	Gpr149 knockout mice displayed increased fertility and enhanced ovulation, with increased levels of FSH receptor and cyclin D2 mRNA levels [491].	–	GPR151 responded to galanin with an EC_50_ value of 2 μM, suggesting that the endogenous ligand shares structural features with galanin (GAL, P22466) [853].	–	–	–

**Table:** 

Nomenclature	GPR161	GPR162	GPR171	GPR173	GPR174	GPR176	GPR182
HGNC, UniProt	GPR161, Q8N6U8	GPR162, Q16538	GPR171, O14626	GPR173, Q9NS66	GPR174, Q9BXC1	GPR176, Q14439	GPR182, O15218
Endogenous agonists	–	–	–	–	lysophosphatidylserine [864]	–	–
Comments	A C‐terminal truncation (deletion) mutation in Gpr161 causes congenital cataracts and neural tube defects in the vacuolated lens (vl) mouse mutant [1289]. The mutated receptor is associated with cataract, spina bifida and white belly spot phenotypes in mice [1039]. Gene disruption is associated with a failure of asymmetric embryonic development in zebrafish [1151].	–	GPR171 has been shown to be activated by the endogenous peptide BigLEN {Mouse}. This receptor‐peptide interaction is believed to be involved in regulating feeding and metabolism responses [654].	–	See [859] which discusses characterization of agonists at this receptor.	–	Rat GPR182 was first proposed as the adrenomedullin receptor [947]. However, it was later reported that rat and human GPR182 did not respond to adrenomedullin [973] and GPR182 is not currently considered to be a genuine adrenomedullin receptor [756].

**Table:** 

Nomenclature	GPR183	LGR4
HGNC, UniProt	GPR183, P32249	LGR4, Q9BXB1
Endogenous agonists	7*α* ,25‐dihydroxycholesterol [729, 1191], 7*α* ,27‐dihydroxycholesterol [1191], 7*β* , 25‐dihydroxycholesterol [1191], 7*β* , 27‐dihydroxycholesterol [1191]	R‐spondin‐2 (RSPO2, Q6UXX9) [277], R‐spondin‐1 (RSPO1, Q2MKA7) [277], R‐spondin‐3 (RSPO3, Q9BXY4) [277], R‐spondin‐4 (RSPO4, Q2I0M5) [277]
Comments	Two independent publications have shown that 7α,25‐dihydroxycholesterol is an agonist of GPR183 and have demonstrated by mass spectrometry that this oxysterol is present endogenously in tissues [729, 1191]. Gpr183‐deficient mice show a reduction in the early antibody response to a T‐dependent antigen. GPR183‐deficient B cells fail to migrate to the outer follicle and instead stay in the follicle centre [966, 1557].	LGR4 does not couple to heterotrimeric G proteins or recruit arrestins when stimulated by the R‐spondins, indicating a unique mechanism of action. R‐spondins bind to LGR4, which specifically associates with Frizzled and LDL receptor‐related proteins (LRPs) that are activated by the extracellular Wnt molecules and then trigger canonical Wnt signalling to increase gene expression [277, 426, 1686]. Gene disruption leads to multiple developmental disorders [911, 1219, 1849, 2092].

**Table:** 

Nomenclature	LGR5	LGR6	MAS1	MAS1L
HGNC, UniProt	LGR5, O75473	LGR6, Q9HBX8	MAS1, P04201	MAS1L, P35410
Endogenous agonists	R‐spondin‐2 (RSPO2, Q6UXX9) [277], R‐spondin‐1 (RSPO1, Q2MKA7) [277], R‐spondin‐3 (RSPO3, Q9BXY4) [277], R‐spondin‐4 (RSPO4, Q2I0M5) [277]	R‐spondin‐1 (RSPO1, Q2MKA7) [277, 426], R‐spondin‐2 (RSPO2, Q6UXX9) [277, 426], R‐spondin‐3 (RSPO3, Q9BXY4) [277, 426], R‐spondin‐4 (RSPO4, Q2I0M5) [277, 426]	–	–
Agonists	–	–	angiotensin‐(1‐7) (AGT, P01019) [645] – Mouse	–
Comments	The four R‐spondins can bind to LGR4, LGR5, and LGR6, which specifically associate with Frizzled and LDL receptor‐related proteins (LRPs), proteins that are activated by extracellular Wnt molecules and which then trigger canonical Wnt signalling to increase gene expression [277, 426].	–	–	–

**Table:** 

Nomenclature	MRGPRD	MRGPRE	MRGPRF	MRGPRG
HGNC, UniProt	MRGPRD, Q8TDS7	MRGPRE, Q86SM8	MRGPRF, Q96AM1	MRGPRG, Q86SM5
Endogenous agonists	*β* ‐alanine [1797, 1854]	–	–	–
Comments	An endogenous peptide with a high degree of sequence similarity to angiotensin‐(1‐7) (AGT, P01019), alamandine (AGT), was shown to promote NO release in MRGPRD‐transfected cells. The binding of alamandine to MRGPRD to was shown to be blocked by D‐Pro^7^‐angiotensin‐(1‐7), *β* ‐alanine and PD123319 [1102]. Genetic ablation of MRGPRD+ neurons of adult mice decreased behavioural sensitivity to mechanical stimuli but not to thermal stimuli [292]. See reviews [414] and [1847].	See reviews [414] and [1847].	MRGPRF has been reported to respond to stimulation by angiotensin metabolites [620]. See reviews [414] and [1847].	See reviews [414] and [1847].

**Table:** 

Nomenclature	MRGPRX1	MRGPRX2	MRGPRX3	MRGPRX4	OPN3	OPN4	OPN5
HGNC, UniProt	MRGPRX1, Q96LB2	MRGPRX2, Q96LB1	MRGPRX3, Q96LB0	MRGPRX4, Q96LA9	OPN3, Q9H1Y3	OPN4, Q9UHM6	OPN5, Q6U736
Endogenous agonists	bovine adrenal medulla peptide 8‐22 (PENK, P01210) [315, 1144, 1854]	PAMP‐20 (ADM, P35318) [942]	–	–	–	–	–
Agonists	–	cortistatin‐14 {Mouse, Rat} [942, 1667, 1854]	–	–	–	–	–
Selective agonists	–	PAMP‐12 (human) [942]	–	–	–	–	–
Comments	Reported to mediate the sensation of itch [1196, 1808]. Reports that bovine adrenal medulla peptide 8‐22 (PENK, P01210) was the most potent of a series of proenkephalin A‐derived peptides as an agonist of MRGPRX1 in assays of calcium mobilisation and radioligand binding [1144] were replicated in an independent study using an arrestin recruitment assay [1854]. See reviews [414] and [1847].	A diverse range of substances has been reported to be agonists of MRGPRX2, with cortistatin 14 the highest potency agonist in assays of calcium mobilisation [1667], also confirmed in an independent study using an arrestin recruitment assay [1854]. See reviews [414] and [1847].	–	See reviews [414] and [1847].	–	–	Evidence indicates OPN5 triggers a UV‐sensitive G_i_‐mediated signalling pathway in mammalian tissues [1028].

**Table:** 

Nomenclature	P2RY8	P2RY10	TAAR2	TAAR3	TAAR4P
HGNC, UniProt	P2RY8, Q86VZ1	P2RY10, O00398	TAAR2, Q9P1P5	TAAR3P, Q9P1P4	TAAR4P, –
Potency order of endogenous ligands	–	–	*β* ‐phenylethylamine>tryptamine [189]	–	–
Endogenous agonists	–	sphingosine 1‐phosphate [1401], LPA [1401]	–	–	–
Comments	–	–	Probable pseudogene in 10–15% of Asians due to a polymorphism (rs8192646) producing a premature stop codon at amino acid 168 [414].	TAAR3 is thought to be a pseudogene in man though functional in rodents [414].	Pseudogene in man but functional in rodents [414].

**Table:** 

Nomenclature	TAAR5	TAAR6	TAAR8	TAAR9
HGNC, UniProt	TAAR5, O14804	TAAR6, Q96RI8	TAAR8, Q969N4	TAAR9, Q96RI9
Comments	Trimethylamine is reported as an agonist [2058] and 3‐iodothyronamine an inverse agonist [450].	–	–	TAAR9 appears to be functional in most individuals but has a polymorphic premature stop codon at amino acid 61 (rs2842899) with an allele frequency of 10–30% in different populations [2023].

## Class C Orphans

**Table:** 

Nomenclature	GPR156	GPR158	GPR179	GPRC5A	GPRC5B	GPRC5C	GPRC5D	GPRC6 receptor
HGNC, UniProt	GPR156, Q8NFN8	GPR158, Q5T848	GPR179, Q6PRD1	GPRC5A, Q8NFJ5	GPRC5B, Q9NZH0	GPRC5C, Q9NQ84	GPRC5D, Q9NZD1	GPRC6A, Q5T6X5
Comments	–	–	–	–	–	–	–	GPRC6 is a related G_q_‐coupled receptor which responds to basic amino acids [2090].

## Taste 1 receptors

### Overview

Whilst the taste of acid and salty foods appear to be sensed by regulation of ion channel activity, bitter, sweet and umami tastes are sensed by specialised GPCR. Two classes of taste GPCR have been identified, T1R and T2R, which are similar in sequence and structure to Class C and Class A GPCR, respectively. Activation of taste receptors appears to involve gustducin‐ (G*α*t3) and G*α*14‐mediated signalling, although the precise mechanisms remain obscure. Gene disruption studies suggest the involvement of PLC*β*2 [2215], TRPM5 [2215] and IP3 [802] receptors in post‐receptor signalling of taste receptors. Although predominantly associated with the oral cavity, taste receptors are also located elsewhere, including further down the gastrointestinal system, in the lungs and in the brain.

Sweet/Umami

T1R3 acts as an obligate partner in T1R1/T1R3 and T1R2/T1R3 heterodimers, which sense umami or sweet, respectively. T1R1/T1R3 heterodimers respond to L‐glutamic acid and may be positively allosterically modulated by 5'‐nucleoside monophosphates, such as 5'‐GMP[1162]. T1R2/T1R3 heterodimers respond to sugars, such as sucrose, and artificial sweeteners, such as saccharin[1440].

**Table:** 

Nomenclature	TAS1R1	TAS1R2	TAS1R3
HGNC, UniProt	TAS1R1, Q7RTX1	TAS1R2, Q8TE23	TAS1R3, Q7RTX0

## Taste 2 receptors

### Overview

The composition and stoichiometry of bitter taste receptors is not yet established. Bitter receptors appear to separate into two groups, with very restricted ligand specificity or much broader responsiveness. For example, T2R5 responded to cycloheximide, but not 10 other bitter compounds [302], while T2R14 responded to at least eight different bitter tastants, including (‐)‐α‐thujone and picrotoxinin[124].

Specialist database BitterDB contains additional information on bitter compounds and receptors [2113].

**Table:** 

Nomenclature	TAS2R1	TAS2R3	TAS2R4	TAS2R5	TAS2R7	TAS2R8	TAS2R9
HGNC, UniProt	TAS2R1, Q9NYW7	TAS2R3, Q9NYW6	TAS2R4, Q9NYW5	TAS2R5, Q9NYW4	TAS2R7, Q9NYW3	TAS2R8, Q9NYW2	TAS2R9, Q9NYW1

**Table:** 

Nomenclature	TAS2R10	TAS2R13	TAS2R14	TAS2R16	TAS2R19	TAS2R20	TAS2R30
HGNC, UniProt	TAS2R10, Q9NYW0	TAS2R13, Q9NYV9	TAS2R14, Q9NYV8	TAS2R16, Q9NYV7	TAS2R19, P59542	TAS2R20, P59543	TAS2R30, P59541

**Table:** 

Nomenclature	TAS2R31	TAS2R38	TAS2R39	TAS2R40
HGNC, UniProt	TAS2R31, P59538	TAS2R38, P59533	TAS2R39, P59534	TAS2R40, P59535
Antagonists	6‐methoxysakuranetin (pIC_50_ 5.6) [1000], GIV3727 (pIC_50_ 5.1–5.2) [1823]	–	–	–

**Table:** 

Nomenclature	TAS2R41	TAS2R42	TAS2R43	TAS2R45	TAS2R46	TAS2R50	TAS2R60
HGNC, UniProt	TAS2R41, P59536	TAS2R42, Q7RTR8	TAS2R43, P59537	TAS2R45, P59539	TAS2R46, P59540	TAS2R50, P59544	TAS2R60, P59551

## Other 7TM proteins

**Table:** 

Nomenclature	GPR107	GPR137	OR51E1	TPRA1	GPR143	GPR157
HGNC, UniProt	GPR107, Q5VW38	GPR137, Q96N19	OR51E1, Q8TCB6	TPRA1, Q86W33	GPR143, P51810	GPR157, Q5UAW9
Endogenous agonists	–	–	–	–	levodopa [1207]	–
Comments	GPR107 is a member of the LUSTR family of proteins found in both plants and animals, having similar topology to G protein‐coupled receptors [489]	–	OR51E1 is a putative olfactory receptor.	TPRA1 shows no homology to known G protein‐coupled receptors.	Loss‐of‐function mutations underlie ocular albinism type 1 [109].	GPR157 has ambiguous sequence similarities to several different GPCR families (class A, class B and the slime mould cyclic AMP receptor). Because of its distant relationship to other GPCRs, it cannot be readily classified.

### Further reading on Orphan and other 7TM receptors

Davenport AP et al. (2013) International Union of Basic and Clinical Pharmacology. LXXXVIII. G protein‐coupled receptor list: recommendations for new pairings with cognate ligands. Pharmacol Rev 65: 967‐86 [PMID:23686350]

Gilissen J et al. (2016) Insight into SUCNR1 (GPR91) structure and function. Pharmacology & Therapeutics 159: 56‐65 [PMID:25118328]

Insel PA et al. (2015) G Protein‐Coupled Receptor (GPCR) Expression in Native Cells: "Novel" endoGPCRs as Physiologic Regulators and Therapeutic Targets. Molecular Pharmacology 88: 181‐187 [PM:25737495]

Khan MZ et al. (2017) Neuro‐psychopharmacological perspective of Orphan receptors of Rhodopsin (class A) family of G protein‐coupled receptors. Psychopharmacology (Berl) 234: 1181‐1207 [PMID:28289782]

Mackenzie AE et al. (2017) The emerging pharmacology and function of GPR35 in the nervous system. Neuropharmacology 113: 661‐671 [PMID:26232640]

Ngo T et al. (2016) Identifying ligands at orphan GPCRs: current status using structure‐based approaches. Br J Pharmacol 173: 2934‐2951 [PMID:26837045]

## 5‐Hydroxytryptamine receptors

### Overview

5‐HT receptors (nomenclature as agreed by the NC‐IUPHAR Subcommittee on 5‐HT receptors [828] and subsequently revised [742]) are, with the exception of the ionotropic 5‐HT_3_ class, GPCRs where the endogenous agonist is 5‐hydroxytryptamine. The diversity of metabotropic 5‐HT receptors is increased by alternative splicing that produces isoforms of the 5‐HT_2A_ (non‐functional), 5‐HT_2C_ (non‐functional), 5‐HT_4_, 5‐HT_6_ (non‐functional) and 5‐HT_7_ receptors. Unique amongst the GPCRs, RNA editing produces 5‐HT_2C_ receptor isoforms that differ in function, such as efficiency and specificity of coupling to G_q/11_ and also pharmacology [167, 2098]. Most 5‐HT receptors (except 5‐ht_1e_ and 5‐ht_5b_) play specific roles mediating functional responses in different tissues (reviewed by [1625, 2037]).

**Table:** 

Nomenclature	5‐HT_1A_ receptor	5‐HT_1B_ receptor
HGNC, UniProt	HTR1A, P08908	HTR1B, P28222
Agonists	U92016A [1302], vilazodone (Partial agonist) [421], vortioxetine (Partial agonist) [96]	L‐694,247 [671], naratriptan (Partial agonist) [1425], eletriptan [1425], frovatriptan [2158], zolmitriptan (Partial agonist) [1425], vortioxetine (Partial agonist) [96], rizatriptan (Partial agonist) [1425]
Selective agonists	8‐OH‐DPAT [431, 720, 939, 1143, 1338, 1451, 1453, 1454], NLX‐101 [1452]	CP94253 [1022]
Antagonists	(S)‐UH 301 (p*K*_i_ 7.9) [1451]	–
Selective antagonists	WAY‐100635 (p*K*_i_ 7.9–9.2) [1451, 1453], robalzotan (p*K*_i_ 9.2) [915]	SB 224289 (Inverse agonist) (p*K*_i_ 8.2–8.6) [614, 1449, 1768], SB236057 (Inverse agonist) (p*K*_i_ 8.2) [1331], GR‐55562 (p*K*_B_ 7.4) [830]
Labelled ligands	[^3^ H]robalzotan (Antagonist) (p*K*_d_ 9.8) [904], [^3^ H]WAY100635 (Antagonist) (p*K*_d_ 9.5) [978], [^3^ H]8‐OH‐DPAT (Agonist) [160, 939, 1450, 1453], [^3^ H]NLX‐112 (Agonist) [785], [^11^ C]WAY100635 (Antagonist) [1991], p‐[^18^ F]MPPF (Antagonist) [382]	[^3^ H]N‐methyl‐AZ10419369 (Agonist, Partial agonist) [1245], [^3^ H]GR 125,743 (Selective Antagonist) (p*K*_d_ 8.6–9.2) [671, 2150], [^3^ H]alniditan (Agonist) [1150], [^125^ I]GTI (Agonist) [197, 237] – Rat, [^3^ H]eletriptan (Agonist, Partial agonist) [1425], [^3^ H]sumatriptan (Agonist, Partial agonist) [1425], [^11^ C]AZ10419369 (Agonist, Partial agonist) [2029]

**Table:** 

Nomenclature	5‐HT_1D_ receptor	5‐ht_1e_ receptor	5‐HT_1F_ receptor
HGNC, UniProt	HTR1D, P28221	HTR1E, P28566	HTR1F, P30939
Agonists	dihydroergotamine [719, 1150, 1157], ergotamine [648], L‐694,247 [2140], naratriptan [457, 1425, 1651], zolmitriptan [1425], frovatriptan [2158], rizatriptan [1425]	BRL‐54443 [232]	BRL‐54443 [232], eletriptan [1425], sumatriptan [12, 13, 1425, 2052]
Selective agonists	PNU109291 [511] – Gorilla, eletriptan [1425]	–	lasmiditan [1439], LY334370 [2052], 5‐BODMT [1014], LY344864 [1572]
Selective antagonists	SB 714786 (p*K*_i_ 9.1) [2074]	–	–
Labelled ligands	[^3^ H]eletriptan (Agonist) [1425], [^3^ H]alniditan (Agonist) [1150], [^125^ I]GTI (Selective Agonist) [197, 237] – Rat, [^3^ H]GR 125,743 (Selective Antagonist) (p*K*_d_ 8.6) [2150], [^3^ H]sumatriptan (Agonist) [1425]	[^3^ H]5‐HT (Agonist) [1299, 1532]	[^3^ H]LY334370 (Agonist) [2052], [^125^ I]LSD (Agonist) [45] – Mouse

**Table:** 

Nomenclature	5‐HT_2A_ receptor	5‐HT_2B_ receptor
HGNC, UniProt	HTR2A, P28223	HTR2B, P41595
Agonists	DOI [210, 1438, 1825]	methysergide (Partial agonist) [1018, 1679, 2053], DOI [1077, 1438, 1730]
Selective agonists	–	BW723C86 [115, 1018, 1730], Ro 60‐0175 [1018]
Antagonists	risperidone (Inverse agonist) (p*K*_i_ 9.3–10) [1032, 1055, 1746], mianserin (p*K*_i_ 7.7–9.6) [1018, 1045, 1338], ziprasidone (p*K*_i_ 8.8–9.5) [1032, 1055, 1746, 1782], volinanserin (pIC_50_ 6.5–9.3) [1018, 1208, 1640], blonanserin (p*K*_i_ 9.1) [1487], clozapine (Inverse agonist) (p*K*_i_ 7.6–9) [1018, 1055, 1335, 1746, 2022]	mianserin (p*K*_i_ 7.9–8.8) [184, 1018, 2053]
Selective antagonists	ketanserin (p*K*_i_ 8.1–9.7) [241, 1018, 1630], pimavanserin (Inverse agonist) (p*K*_i_ 9.3) [603, 2022]	BF‐1 (p*K*_i_ 10.1) [1742], RS‐127445 (p*K*_i_ 9–9.5) [184, 1018], EGIS‐7625 (p*K*_i_ 9) [1045]
Labelled ligands	[^3^ H]fananserin (Antagonist) (p*K*_d_ 9.9) [1251] – Rat, [^3^ H]ketanserin (Antagonist) (p*K*_d_ 8.6–9.7) [1018, 1630], [^11^ C]volinanserin (Antagonist) [712], [^18^ F]altanserin (Antagonist) [1675]	[^3^ H]LSD (Agonist) [1630], [^3^ H]5‐HT (Agonist) [2051] – Rat, [^3^ H]mesulergine (Antagonist, Inverse agonist) (p*K*_d_ 7.9) [1018], [^125^ I]DOI (Agonist)

**Table:** 

Nomenclature	5‐HT_2C_ receptor	5‐HT_4_ receptor
HGNC, UniProt	HTR2C, P28335	HTR4, Q13639
Agonists	DOI [493, 1438, 1730], Ro 60‐0175 [999, 1018]	cisapride (Partial agonist) [80, 132, 631, 1326, 1327, 2013]
Selective agonists	WAY‐163909 [482], lorcaserin [1955]	TD‐8954 [1312], ML 10302 (Partial agonist) [140, 164, 1326, 1327, 1328], RS67506 [765] – Rat, relenopride (Partial agonist) [641], velusetrag [1205, 1832], BIMU 8 [362]
Antagonists	mianserin (Inverse agonist) (p*K*_i_ 8.3–9.2) [551, 1018, 1338], methysergide (p*K*_i_ 8.6–9.1) [493, 1018], ziprasidone (Inverse agonist) (p*K*_i_ 7.9–9) [779, 1055, 1782], olanzapine (Inverse agonist) (p*K*_i_ 8.1–8.4) [779, 1055, 1782], loxapine (Inverse agonist) (p*K*_i_ 7.8–8) [779, 1055]	–
Selective antagonists	FR260010 (p*K*_i_ 9) [735], SB 242084 (p*K*_i_ 8.2–9) [974, 1018], RS‐102221 (p*K*_i_ 8.3–8.4) [185, 1018]	RS 100235 (p*K*_i_ 8.7–12.2) [362, 1663], SB 204070 (p*K*_i_ 9.8–10.4) [132, 1326, 1327, 2013], GR 113808 (p*K*_i_ 9.3–10.3) [80, 132, 164, 362, 1327, 1663, 2013]
Labelled ligands	[^3^ H]mesulergine (Antagonist, Inverse agonist) (p*K*_d_ 8.7–9.3) [551, 1630], [^125^ I]DOI (Agonist) [551], [^3^ H]LSD (Agonist)	[^3^ H]GR 113808 (Antagonist) (p*K*_d_ 9.7–10.3) [80, 132, 1328, 2013], [^123^ I]SB 207710 (Antagonist) (p*K*_d_ 10.1) [233] – Pig, [^3^ H]RS 57639 (Selective Antagonist) (p*K*_d_ 9.7) [183] – Guinea pig, [^11^ C]SB207145 (Antagonist) (p*K*_d_ 8.6) [1233]

**Table:** 

Nomenclature	5‐HT_5A_ receptor	5‐ht_5b_ receptor	5‐HT_6_ receptor	5‐HT_7_ receptor
HGNC, UniProt	HTR5A, P47898	HTR5BP, –	HTR6, P50406	HTR7, P34969
Selective agonists	–	–	WAY‐181187 [1734], E6801 (Partial agonist) [808], WAY‐208466 [139], EMD‐386088 [1291]	LP‐12 [1148], LP‐44 [1148], LP‐211 [1149] – Rat, AS‐19 [993], E55888 [212]
Antagonists	–	–	–	lurasidone (p*K*_i_ 9.3) [868], pimozide (p*K*_i_ 9.3) [1678] – Rat, vortioxetine (p*K*_i_ 6.3) [96]
Selective antagonists	SB 699551 (p*K*_i_ 8.2) [380]	–	SB399885 (p*K*_i_ 9) [801], SB 271046 (p*K*_i_ 8.9) [229], cerlapirdine (p*K*_i_ 8.9) [371], SB357134 (p*K*_i_ 8.5) [230], Ro 63‐0563 (p*K*_i_ 7.9–8.4) [170, 1824]	SB269970 (p*K*_i_ 8.6–8.9) [1949], SB656104 (p*K*_i_ 8.7) [558], DR‐4004 (p*K*_i_ 8.7) [647, 985], JNJ‐18038683 (p*K*_i_ 8.2) [181], SB 258719 (Inverse agonist) (p*K*_i_ 7.5) [1950]
Labelled ligands	[^125^ I]LSD (Agonist) [670], [^3^ H]5‐CT (Agonist) [670]	[^125^ I]LSD (Agonist) [1290] – Mouse, [^3^ H]5‐CT (Agonist) [2049] – Mouse	[^11^ C]GSK215083 (Antagonist) (p*K*_i_ 9.8) [1531], [^125^ I]SB258585 (Selective Antagonist) (p*K*_d_ 9) [801], [^3^ H]LSD (Agonist) [169], [^3^ H]Ro 63‐0563 (Antagonist) (p*K*_d_ 8.3) [170], [^3^ H]5‐CT (Agonist)	[^3^ H]5‐CT (Agonist) [1949], [^3^ H]5‐HT (Agonist) [99, 1864], [^3^ H]SB269970 (Selective Antagonist) (p*K*_d_ 8.9) [1949], [^3^ H]LSD (Agonist) [1864]

### Comments

Tabulated pK_i_ and K_D_ values refer to binding to human 5‐HT receptors unless indicated otherwise. The nomenclature of 5‐HT_1B_/5‐HT_1D_ receptors has been revised [742]. Only the non‐rodent form of the receptor was previously called 5‐HT_1D_: the human 5‐HT_1B_ receptor (tabulated) displays a different pharmacology to the rodent forms of the receptor due to Thr335 of the human sequence being replaced by Asn in rodent receptors. Wang et al. (2013) report X‐ray structures which reveal the binding modality of ergotamine and dihydroergotamine to the 5‐HT_1B_ receptor in comparison with the structure of the 5‐HT_2B_ receptor [2064]. NAS181 is a selective antagonist of the rodent 5‐HT_1B_ receptor. Fananserin and ketanserin bind with high affinity to dopamine D4 and histamine H_1_ receptors respectively, and ketanserin is a potent *α*1 adrenoceptor antagonist, in addition to blocking 5‐HT_2A_ receptors. Lysergic acid(LSD) and ergotamine show a strong preference for arrestin recruitment over G protein coupling at the 5‐HT_2B_ receptor, with no such preference evident at 5‐HT_1B_ receptors, and they also antagonise 5‐HT_7A_ receptors [2047]. DHE (dihydroergocryptine), pergolide and cabergoline also show significant preference for arrestin recruitment over G protein coupling at 5‐HT_2B_ receptors [2047]. The serotonin antagonist mesulergine was key to the discovery of the 5‐HT_2C_ receptor [1546]. The human 5‐HT_5A_ receptor has been claimed to couple to several signal transduction pathways when stably expressed in C6 glioma cells [1472] and electrophysiological recordings from mice and rat prefrontal cortex (layer V pyramidal neurons) demonstrate 5‐HT‐elicited outward currents mediated via the 5‐HT_5A_ receptor [660]. The human orthologue of the mouse 5‐ht_5b_ receptor is non‐functional due to interruption of the gene by stop codons. The 5‐ht_1e_ receptor appears not to have been cloned from mouse, or rat, impeding definition of its function. In addition to the receptors listed in the table, an 'orphan' receptor, unofficially termed 5‐HT_1P_, has been described [635]. .

### Further reading on 5‐Hydroxytryptamine receptors

Bockaert J et al. (2011) 5‐HT(4) receptors, a place in the sun: act two. Curr Opin Pharmacol 11: 87‐93 [PMID:21342787]

Hayes DJ et al. (2011) 5‐HT receptors and reward‐related behaviour: a review. Neurosci Biobehav Rev 35: 1419‐49 [PMID:21402098]

Hoyer D et al. (1994) International Union of Pharmacology classification of receptors for 5‐hydroxytryptamine (Serotonin). Pharmacol. Rev. 46: 157‐203 [PMID:7938165]

Leopoldo M et al. (2011) Serotonin 5‐HT7 receptor agents: Structure‐activity relationships and potential therapeutic applications in central nervous system disorders. Pharmacol. Ther. 129: 120‐48 [PMID:20923682]

Meltzer HY et al. (2011) The role of serotonin receptors in the action of atypical antipsychotic drugs. Curr Opin Pharmacol 11: 59‐67 [PMID:21420906]

Roberts AJ et al. (2012) The 5‐HT(7) receptor in learning and memory. Hippocampus 22: 762‐71 [PMID:21484935]

## Acetylcholine receptors (muscarinic)

### Overview

Muscarinic acetylcholine receptors (nomenclature as agreed by the NC‐IUPHAR Subcommittee on Muscarinic Acetylcholine Receptors [288]) are GPCRs of the Class A, rhodopsin‐like family where the endogenous agonist is acetylcholine. In addition to the agents listed in the table, AC‐42, its structural analogues AC‐260584 and 77‐LH‐28‐1, N‐desmethylclozapine, TBPB and LuAE51090 have been described as functionally selective agonists of the M_1_ receptor subtype via binding in a mode distinct from that utilized by non‐selective agonists [74, 921, 1096, 1097, 1294, 1708, 1857, 1858, 1898]. There are two pharmacologically characterised allosteric sites on muscarinic receptors, one defined by it binding gallamine, strychnine and brucine, and the other defined by the binding of KT 5720, WIN 62,577, WIN 51,708 and staurosporine[1110, 1111].

**Table:** 

Nomenclature	M_1_ receptor	M_2_ receptor
HGNC, UniProt	CHRM1, P11229	CHRM2, P08172
Agonists	carbachol [350, 888, 2129], pilocarpine (Partial agonist) [888], bethanechol [888]	bethanechol [888]
Antagonists	glycopyrrolate (pIC_50_ 9.9) [1874], umeclidinium (p*K*_i_ 9.8) [1090, 1705], AE9C90CB (p*K*_i_ 9.7) [1818], propantheline (p*K*_i_ 9.7) [837], atropine (p*K*_i_ 8.5–9.6) [350, 582, 797, 837, 1555, 1831], tiotropium (p*K*_i_ 9.6) [452], 4‐DAMP (p*K*_i_ 9.2) [486]	tiotropium (p*K*_i_ 9.9) [452], umeclidinium (p*K*_i_ 9.8) [1090, 1705], propantheline (p*K*_i_ 9.5) [837], glycopyrrolate (Full agonist) (pIC_50_ 9.3) [1874], atropine (p*K*_i_ 7.8–9.2) [245, 325, 797, 837, 1046, 1437, 1555], AE9C90CB (p*K*_i_ 8.6) [1818], tolterodine (Inverse agonist) (p*K*_i_ 8.4–8.6) [642, 1437, 1818]
Selective antagonists	biperiden (p*K*_d_ 9.3) [175], VU0255035 (p*K*_i_ 7.8) [1786], guanylpirenzepine (p*K*_i_ 7.3–7.6) [23, 2050] – Rat	tripitramine (p*K*_i_ 9.6) [1240]
Allosteric modulators	muscarinic toxin 7 (Negative) (p*K*_i_ 11–11.1) [1480], benzoquinazolinone 12 (Positive) (p*K*_B_ 6.6) [4], KT 5720 (Positive) (p*K*_d_ 6.4) [1110], brucine (Positive) (p*K*_d_ 4.5–5.8) [888, 1109], BQCA (Positive) (p*K*_B_ 4–4.8) [4, 5, 271, 1225], VU0029767 (Positive) [1270], VU0090157 (Positive) [1270]	W‐84 (Negative) (p*K*_d_ 6–7.5) [1353, 1983], C_7_ /3‐phth (Negative) (p*K*_d_ 7.1) [351], alcuronium (Negative) (p*K*_d_ 6.1–6.9) [888, 1983], gallamine (Negative) (p*K*_d_ 5.9–6.3) [363, 1107], LY2119620 (Positive) (p*K*_d_ 5.7) [399, 1057], LY2033298 (Positive) (p*K*_d_ 4.4) [2009]
Labelled ligands	[^3^ H]QNB (Antagonist) (p*K*_d_ 10.6–10.8) [352, 1555], Cy3B‐telenzepine (Antagonist) (p*K*_d_ 10.5) [777], [^3^ H]N‐methyl scopolamine (Antagonist) (p*K*_d_ 9.4–10.3) [294, 350, 352, 797, 888, 889, 918, 977, 1107], [^3^ H](+)telenzepine (Antagonist) (p*K*_i_ 9.4) [526] – Rat, Alexa‐488‐telenzepine (Antagonist) (p*K*_d_ 9.3) [777], [^3^ H]pirenzepine (Antagonist) (p*K*_d_ 7.9) [2083], BODIPY‐pirenzepine (Antagonist) (p*K*_i_ 7) [860], [^11^ C]butylthio‐TZTP (Agonist) [530], [^11^ C]xanomeline (Agonist) [530], [^18^ F](R,R)‐quinuclidinyl‐4‐fluoromethyl‐benzilate (Antagonist) [982] – Rat	[^3^ H]QNB (Antagonist) (p*K*_d_ 10.1–10.6) [1555], Cy3B‐telenzepine (Antagonist) (p*K*_i_ 10.4) [1444], [^3^ H]tiotropium (Antagonist) (p*K*_d_ 10.3) [1705], [^3^ H]N‐methyl scopolamine (Antagonist) (p*K*_d_ 9.3–9.9) [294, 325, 797, 888, 889, 918, 977, 1107, 2072], Alexa‐488‐telenzepine (Antagonist) (p*K*_i_ 8.8) [1444], [^3^ H]acetylcholine (Agonist) [1108], [^3^ H]oxotremorine‐M (Agonist) [141], [^3^ H]dimethyl‐W84 (Allosteric modulator, Positive) (p*K*_d_ 8.5) [1983], [^18^ F]FP‐TZTP (Agonist) [887] – Mouse

**Table:** 

Nomenclature	M_3_ receptor	M_4_ receptor	M_5_ receptor
HGNC, UniProt	CHRM3, P20309	CHRM4, P08173	CHRM5, P08912
Agonists	pilocarpine (Partial agonist) [888], carbachol [325, 888, 2129], bethanechol [888]	pilocarpine (Partial agonist) [888], carbachol [888, 2129], bethanechol [888]	pilocarpine (Partial agonist) [673], carbachol [2129]
Antagonists	tiotropium (p*K*_i_ 9.5–11.1) [452, 469], umeclidinium (p*K*_i_ 10.2) [1090, 1705], propantheline (p*K*_i_ 10) [837], AE9C90CB (p*K*_i_ 9.9) [1818], atropine (p*K*_i_ 8.9–9.8) [245, 469, 797, 837, 1555, 1831], ipratropium (p*K*_i_ 9.3–9.8) [469, 797], aclidinium (pIC_50_ 9.8) [1601]	umeclidinium (p*K*_i_ 10.3) [1705], glycopyrrolate (pIC_50_ 9.8) [1874], AE9C90CB (p*K*_i_ 9.5) [1818], 4‐DAMP (p*K*_i_ 8.9) [486], oxybutynin (p*K*_i_ 8.7) [1818], biperiden (p*K*_d_ 8.6) [175], UH‐AH 37 (p*K*_i_ 8.3–8.4) [642, 2099]	umeclidinium (p*K*_i_ 9.9) [1705], glycopyrrolate (pIC_50_ 9.7) [1874], AE9C90CB (p*K*_i_ 9.5) [1818], 4‐DAMP (p*K*_i_ 9) [486], tolterodine (p*K*_i_ 8.5–8.8) [642, 1818], darifenacin (p*K*_i_ 7.9–8.6) [642, 764, 797, 1818]
Selective antagonists	–	–	ML381 (p*K*_i_ 6.3) [625]
Allosteric modulators	WIN 62,577 (Positive) (p*K*_d_ 5.1) [1111], N‐chloromethyl‐brucine (Positive) (p*K*_d_ 3.3) [1109]	muscarinic toxin 3 (Negative) (p*K*_i_ 8.7) [918, 1512], VU0152100 (Positive) (pEC_50_ 6.4) [207] – Rat, VU0152099 (Positive) (pEC_50_ 6.4) [207] – Rat, LY2119620 (Positive) (p*K*_d_ 5.7) [399], thiochrome (Positive) (p*K*_d_ 4) [1108], LY2033298 (Positive) [301]	ML380 (Positive) (pEC_50_ 6.7) [627]
Selective allosteric modulators	–	–	ML375 (Negative) (pIC_50_ 6.5) [626]
Labelled ligands	[^3^ H]tiotropium (Antagonist) (p*K*_d_ 10.7) [1705], [^3^ H]QNB (Antagonist) (p*K*_d_ 10.4) [1555], [^3^ H]N‐methyl scopolamine (Antagonist) (p*K*_d_ 9.7–10.2) [294, 325, 797, 837, 888, 918, 977, 1107], [^3^ H]darifenacin (Antagonist) (p*K*_d_ 9.5) [1831]	[^3^ H]QNB (Antagonist) (p*K*_d_ 9.7–10.5) [352, 1555], [^3^ H]N‐methyl scopolamine (Antagonist) (p*K*_d_ 9.9–10.2) [294, 325, 352, 797, 888, 918, 977, 1107, 1512, 2072], [^3^ H]acetylcholine (Agonist) [1108]	[^3^ H]QNB (Antagonist) (p*K*_d_ 10.2–10.7), [^3^ H]N‐methyl scopolamine (Antagonist) (p*K*_d_ 9.3–9.7) [294, 325, 797, 918, 977, 2072]

### Comments

LY2033298 and BQCA have also been shown to directly activate the M_4_ and M_1_ receptors, respectively, via an allosteric site [1119, 1121, 1427, 1428]. The allosteric site for gallamine and strychnine on M_2_ receptors can be labelled by [^3^ H]dimethyl‐W84[1983]. McN‐A‐343 is a functionally selective partial agonist that appears to interact in a bitopic mode with both the orthosteric and an allosteric site on the M_2_ muscarinic receptor [2010]. THRX160209, hybrid 1 and hybrid 2, are multivalent (bitopic) ligands that also achieve selectivity for M_2_ receptors by binding both to the orthosteric and a nearby allosteric site [55, 1866].

Although numerous ligands for muscarinic acetylcholine receptors have been described, relatively few selective antagonists have been described, so it is common to assess the rank order of affinity of a number of antagonists of limited selectivity (e.g. 4‐DAMP, darifenacin, pirenzepine) in order to identify the involvement of particular subtypes. It should be noted that the measured affinities of antagonists (and agonists) in radioligand binding studies are sensitive to ionic strength and can increase over 10‐fold at low ionic strength compared to their values at physiological ionic strengths [155].

### Further reading on Acetylcholine receptors (muscarinic)

Caulfield MP et al. (1998) International Union of Pharmacology. XVII. Classification of muscarinic acetylcholine receptors. Pharmacol. Rev. 50: 279‐290 [PMID:9647869]

Eglen RM. (2012) Overview of muscarinic receptor subtypes. Handb Exp Pharmacol 3‐28 [PMID:22222692]

Gregory KJ et al. (2007) Allosteric modulation of muscarinic acetylcholine receptors. Curr Neuropharmacol 5: 157‐67 [PMID:19305798]

Kruse AC et al. (2014) Muscarinic acetylcholine receptors: novel opportunities for drug development. Nat Rev Drug Discov 13: 549‐60 [PMID:24903776]

Leach K et al. (2012) Structure‐function studies of muscarinic acetylcholine receptors. Handb Exp Pharmacol 29‐48 [PMID:22222693]

Valant C et al. (2012) The best of both worlds? Bitopic orthosteric/allosteric ligands of g protein‐coupled receptors. Annu. Rev. Pharmacol. Toxicol. 52: 153‐78 [PMID:21910627]

## Adenosine receptors

### Overview

Adenosine receptors (nomenclature as agreed by the NC‐IUPHAR Subcommittee on Adenosine Receptors [569]) are activated by the endogenous ligand adenosine(potentially inosine also at A_3_ receptors). Crystal structures for the antagonist‐bound [374, 876, 1198, 1765], agonist‐bound [1126, 1127, 2154] and G protein‐bound A_2A_ adenosine receptors [278] have been described.

**Table:** 

Nomenclature	A_1_ receptor	A_2A_ receptor	A_2B_ receptor	A_3_ receptor
HGNC, UniProt	ADORA1, P30542	ADORA2A, P29274	ADORA2B, P29275	ADORA3, P0DMS8
Sub/family‐selective agonists	NECA [602, 913, 1665, 1980, 2166]	NECA [193, 451, 602, 990, 1071, 2166]	NECA [148, 193, 905, 1179, 1870, 2024, 2166]	NECA [193, 602, 883, 1707, 2025, 2166]
Selective agonists	cyclopentyladenosine [404, 428, 602, 771, 880, 913, 1665], 5‐Cl‐5‐deoxy‐(±)‐ENBA [565], TCPA [150], CCPA [880, 1485]	apadenoson [1548], UK‐432,097 [2154], compound 4g [374], CGS 21680 [193, 451, 602, 880, 990, 1016, 1071, 1485], regadenoson [880]	BAY 60‐6583 [488]	piclidenoson [537, 592, 1016, 2025], Cl‐IB‐MECA [208, 883, 987], MRS5698 [1977]
Sub/family‐selective antagonists	CGS 15943 (p*K*_i_ 8.5) [1513], xanthine amine congener (p*K*_d_ 7.5) [565]	CGS 15943 (p*K*_i_ 7.7–9.4) [451, 990, 1016, 1513], xanthine amine congener (p*K*_i_ 8.4–9) [451, 1016]	xanthine amine congener (p*K*_i_ 6.9–8.8) [148, 905, 906, 1016, 1179, 1870], CGS 15943 (p*K*_i_ 6–8.1) [68, 905, 906, 1016, 1513, 1870]	CGS 15943 (p*K*_i_ 7–7.9) [995, 1016, 1513, 2025], xanthine amine congener (p*K*_i_ 7–7.4) [1016, 1707, 2025]
Selective antagonists	PSB36 (p*K*_i_ 9.9) [6] – Rat, DPCPX (p*K*_i_ 7.4–9.2) [428, 865, 1485, 1665, 2102], derenofylline (p*K*_i_ 9) [939], WRC‐0571 (p*K*_i_ 8.8) [1272], DU172 (p*K*_i_ 7.4) [649]	SCH442416 (p*K*_i_ 8.4–10.3) [1796, 1969], ZM‐241385 (p*K*_i_ 8.8–9.1) [1513]	PSB‐0788 (p*K*_i_ 9.4) [192], PSB603 (p*K*_i_ 9.3) [192], MRS1754 (p*K*_i_ 8.8) [905, 994], PSB1115 (p*K*_i_ 7.3) [757]	MRS1220 (p*K*_i_ 8.2–9.2) [883, 995, 1892, 2177], VUF5574 (p*K*_i_ 8.4) [2016], MRS1523 (p*K*_i_ 7.7) [1158], MRS1191 (p*K*_i_ 7.5) [883, 909, 1163]
Allosteric modulators	PD81723 (Positive) [239]	–	–	LUF6000 (Positive) [701], LUF6096 (Positive) [770]
Labelled ligands	[^3^ H]CCPA (Agonist) [1016, 1665], [^3^ H]DPCPX (Antagonist) (p*K*_d_ 8.4–9.2) [404, 537, 1016, 1513, 1665, 1980]	[^3^ H]ZM 241385 (Antagonist) (p*K*_d_ 8.7–9.1) [36, 600], [^3^ H]CGS 21680 (Agonist) [894, 2062]	[^3^ H]MRS1754 (Antagonist) (p*K*_d_ 9.8) [905]	[^125^ I]AB‐MECA (Agonist) [1513, 2025]

### Comments

Adenosine inhibits many intracellular ATP‐utilising enzymes, including adenylyl cyclase (P‐site). A pseudogene exists for the A_2B_ adenosine receptor (ADORA2BP1) with 79% identity to the A_2B_ adenosine receptor cDNA coding sequence, but which is unable to encode a functional receptor [884]. DPCPX also exhibits antagonism at A_2B_ receptors (pK_i_ ca. 7,[34, 1016]). Antagonists at A_3_ receptors exhibit marked species differences, such that only MRS1523 and MRS1191 are selective at the rat A_3_ receptor. In the absence of other adenosine receptors, [^3^ H]DPCPX and [^3^ H]ZM 241385 can also be used to label A_2B_ receptors (K_D_ ca. 30 and 60 nM respectively). [^125^ I]AB‐MECA also binds to A_1_ receptors [1016]. [^3^ H]CGS 21680 is relatively selective for A_2A_ receptors, but may also bind to other sites in cerebral cortex [400, 914]. [^3^ H]NECA binds to other non‐receptor elements, which also recognise adenosine [1209]. XAC‐BY630 has been described as a fluorescent antagonist for labelling A_1_ adenosine receptors in living cells, although activity at other adenosine receptors was not examined [217].

### Further reading on Adenosine receptors

Fredholm BB et al. (2011) International Union of Basic and Clinical Pharmacology. LXXXI. Nomenclature and classification of adenosine receptors–an update. Pharmacol. Rev. 63: 1‐34 [PMID:21303899]

Guo D et al. (2017) Kinetic Aspects of the Interaction between Ligand and G Protein‐Coupled Receptor: The Case of the Adenosine Receptors. Chem. Rev. 117: 38‐66 [PMID:27088232]

Göblyös A et al. (2011) Allosteric modulation of adenosine receptors. Biochim. Biophys. Acta 1808: 1309‐18 [PMID:20599682]

Headrick JP et al. (2011) Adenosine and its receptors in the heart: regulation, retaliation and adaptation. Biochim. Biophys. Acta 1808: 1413‐28 [PMID:21094127]

Lasley RD. (2011) Adenosine receptors and membrane microdomains. Biochim. Biophys. Acta 1808: 1284‐9 [PMID:20888790]

Mundell S et al. (2011) Adenosine receptor desensitization and trafficking. Biochim. Biophys. Acta 1808: 1319‐28 [PMID:20550943]

Wei CJ et al. (2011) Normal and abnormal functions of adenosine receptors in the central nervous system revealed by genetic knockout studies. Biochim. Biophys. Acta 1808: 1358‐79 [PMID:21185258]

## Adhesion Class GPCRs

### Overview

Adhesion GPCRs are structurally identified on the basis of a large extracellular region, similar to the Class B GPCR, but which is linked to the 7TM region by a GPCR autoproteolysis‐inducing (GAIN) domain [56] containing a GPCR proteolytic site. The N‐terminus often shares structural homology with proteins such as lectins and immunoglobulins, leading to the term adhesion GPCR [571, 2187]. The nomenclature of these receptors was revised in 2015 as recommended by NC‐IUPHAR and the Adhesion GPCR Consortium [718].

**Table:** 

Nomenclature	ADGRA1	ADGRA2	ADGRA3	ADGRB1	ADGRB2	ADGRB3	CELSR1
HGNC, UniProt	ADGRA1, Q86SQ6	ADGRA2, Q96PE1	ADGRA3, Q8IWK6	ADGRB1, O14514	ADGRB2, O60241	ADGRB3, O60242	CELSR1, Q9NYQ6
Endogenous agonists	–	–	–	phosphatidylserine [1530]	–	–	–
Comments	–	–	–	ADGRB1 is reported to respond to phosphatidylserine [1530].	–	–	–

**Table:** 

Nomenclature	CELSR2	CELSR3	ADGRD1	ADGRD2	ADGRE1	ADGRE2	ADGRE3
HGNC, UniProt	CELSR2, Q9HCU4	CELSR3, Q9NYQ7	ADGRD1, Q6QNK2	ADGRD2, Q7Z7M1	ADGRE1, Q14246	ADGRE2, Q9UHX3	ADGRE3, Q9BY15
Comments	–	–	–	–	–	A mutation destabilizing the GAIN domain sensitizes mast cells to IgE‐independent vibration‐induced degranulation [202].	–

**Table:** 

Nomenclature	ADGRE4P	ADGRE5	ADGRF1	ADGRF2	ADGRF3	ADGRF4	ADGRF5
HGNC, UniProt	ADGRE4P, Q86SQ3	ADGRE5, P48960	ADGRF1, Q5T601	ADGRF2, Q8IZF7	ADGRF3, Q8IZF5	ADGRF4, Q8IZF3	ADGRF5, Q8IZF2

**Table:** 

Nomenclature	ADGRG1	ADGRG2	ADGRG3	ADGRG4	ADGRG5
HGNC, UniProt	ADGRG1, Q9Y653	ADGRG2, Q8IZP9	ADGRG3, Q86Y34	ADGRG4, Q8IZF6	ADGRG5, Q8IZF4
Comments	Reported to bind tissue transglutaminase 2 [2155] and collagen, which activates the G_12/13_ pathway [1220].	–	–	–	–

**Table:** 

Nomenclature	ADGRG6	ADGRG7	ADGRL1	ADGRL2	ADGRL3	ADGRL4	ADGRV1
HGNC, UniProt	ADGRG6, Q86SQ4	ADGRG7, Q96K78	ADGRL1, O94910	ADGRL2, O95490	ADGRL3, Q9HAR2	ADGRL4, Q9HBW9	ADGRV1, Q8WXG9
Comments	–	–	–	–	–	–	Loss‐of‐function mutations are associated with Usher syndrome, a sensory deficit disorder [885].

### Further reading on Adhesion Class GPCRs

Hamann J et al. (2015) International Union of Basic and Clinical Pharmacology. XCIV. Adhesion G protein‐coupled receptors. Pharmacol. Rev. 67: 338‐67 [PMID:25713288]

Yona S et al. (2008) Adhesion‐GPCRs: emerging roles for novel receptors. Trends Biochem. Sci. 33: 491‐500 [PMID:18789697]

## Adrenoceptors

### Overview

The nomenclature of the Adrenoceptors has been agreed by the NC‐IUPHAR Subcommittee on Adrenoceptors [256], see also [789].

Adrenoceptors, *α*_1_

*α*_1_‐Adrenoceptors are activated by the endogenous agonists (‐)‐adrenaline and (‐)‐noradrenaline. Phenylephrine, methoxa‐ mine and cirazoline are agonists and prazosin and cirazoline antagonists considered selective for *α*_1_‐ relative to *α*_2_‐adrenoceptors. [^3^ H]prazosin and [^125^ I]HEAT(BE2254) are relatively selective radioligands. S(+)‐niguldipine also has high affinity for L‐type Ca^2+^ channels. Fluorescent derivatives of prazosin(Bodipy PLprazosin‐ QAPB) are used to examine cellular localisation of *α*_1_‐adrenoceptors. Selective *α*_1_‐adrenoceptor agonists are used as nasal decongestants; antagonists to treat hypertension (doxazosin, prazosin) and benign prostatic hyperplasia (alfuzosin, tamsulosin). The *α*_1_‐ and *β*_2_‐adrenoceptor antagonist carvedilol is used to treat congestive heart failure, although the contribution of *α*_1_‐adrenoceptor blockade to the therapeutic effect is unclear. Several anti‐depressants and anti‐psychotic drugs are *α*_1_‐adrenoceptor antagonists contributing to side effects such as orthostatic hypotension and extrapyramidal effects.

**Table:** 

Nomenclature	*α*_1A_ ‐adrenoceptor	*α*_1B_ ‐adrenoceptor	*α*_1D_ ‐adrenoceptor
HGNC, UniProt	ADRA1A, P35348	ADRA1B, P35368	ADRA1D, P25100
Endogenous agonists	(‐)‐adrenaline [819, 1789], (‐)‐noradrenaline [819, 1789, 1936]	–	(‐)‐noradrenaline [819, 1789], (‐)‐adrenaline [819, 1789]
Agonists	oxymetazoline [819, 1486, 1789, 1936], phenylephrine [1936], methoxamine [1789, 1936]	phenylephrine [559, 1345]	–
Selective agonists	A61603 [559, 1017], dabuzalgron [165]	–	–
Antagonists	prazosin (Inverse agonist) (p*K*_i_ 9–9.9) [303, 405, 559, 1789, 2118], doxazosin (p*K*_i_ 9.3) [724], terazosin (p*K*_i_ 8.7) [1323], phentolamine (p*K*_i_ 8.6) [1789], alfuzosin (p*K*_i_ 8.1) [787]	prazosin (Inverse agonist) (p*K*_i_ 9.6–9.9) [559, 1789, 2118], tamsulosin (Inverse agonist) (p*K*_i_ 9.5–9.7) [559, 1789, 2118], doxazosin (p*K*_i_ 9.1) [724], alfuzosin (p*K*_i_ 8.6) [788], terazosin (p*K*_i_ 8.6) [1323], phentolamine (p*K*_i_ 7.5) [1789]	prazosin (Inverse agonist) (p*K*_i_ 9.5–10.2) [559, 1789, 2118], tamsulosin (p*K*_i_ 9.8–10.2) [559, 1789, 2118], doxazosin (p*K*_i_ 9.1) [724], terazosin (p*K*_i_ 9.1) [1323], alfuzosin (p*K*_i_ 8.4) [787], dapiprazole (p*K*_i_ 8.4) [71], phentolamine (Inverse agonist) (p*K*_i_ 8.2) [1789], RS‐100329 (p*K*_i_ 7.9) [2118], labetalol (p*K*_i_ 6.6) [71]
Selective antagonists	tamsulosin (p*K*_i_ 10–10.7) [303, 405, 559, 1789, 2118], silodosin (p*K*_i_ 10.4) [1789], S(+)‐niguldipine (p*K*_i_ 9.1–10) [559, 1789], RS‐100329 (p*K*_i_ 9.6) [2118], SNAP5089 (p*K*_i_ 8.8–9.4) [787, 1147, 2101], *ρ* ‐Da1a (p*K*_i_ 9.2–9.3) [1298, 1619], RS‐17053 (p*K*_i_ 9.2–9.3) [303, 405, 556, 559]	Rec 15/2615 (p*K*_i_ 9.5) [1942], L‐765314 (p*K*_i_ 7.7) [1537], AH 11110 (p*K*_i_ 7.5) [1724]	BMY‐7378 (p*K*_i_ 8.7–9.1) [280, 2192]

Adrenoceptors, *α*_2_

*α*_2_‐Adrenoceptors are activated by (‐)‐adrenaline and with lower potency by (‐)‐noradrenaline. Brimonidine and talipexole are agonists and rauwolscine and yohimbine antagonists selective for *α*_2_‐ relative to *α*_1_‐adrenoceptors. [^3^ H]rauwolscine, [^3^ H] brimonidine and [^3^ H]RX821002 are relatively selective radioligands. There is species variation in the pharmacology of the *α*_2A_‐adrenoceptor. Multiple mutations of *α*_2_‐adrenoceptors have been described, some associated with alterations in function. Presynaptic *α*_2_‐adrenoceptors regulate many functions in the nervous system. The *α*_2_‐adrenoceptor agonists clonidine, guanabenz and brimonidine affect central baroreflex control (hypotension and bradycardia), induce hypnotic effects and analgesia, and modulate seizure activity and platelet aggregation. Clonidine is an anti‐hypertensive and counteracts opioid withdrawal. Dexmedetomidine (also xylazine) is used as a sedative and analgesic in human and veterinary medicine with sympatholytic and anxiolytic properties. The *α*_2_‐adrenoceptor antagonist yohimbine has been used to treat erectile dysfunction and mirtazapine as an anti‐depressant. The *α*_2B_ subtype appears to be involved in neurotransmission in the spinal cord and *α*_2C_ in regulating catecholamine release from adrenal chromaffin cells.

**Table:** 

Nomenclature	*α*_2A_ ‐adrenoceptor	*α*_2B_ ‐adrenoceptor	*α*_2C_ ‐adrenoceptor
HGNC, UniProt	ADRA2A, P08913	ADRA2B, P18089	ADRA2C, P18825
Endogenous agonists	(‐)‐adrenaline [896, 1573], (‐)‐noradrenaline [896, 1573]	(‐)‐noradrenaline (Partial agonist) [896, 1573], (‐)‐adrenaline [896]	(‐)‐noradrenaline [896, 1573], (‐)‐adrenaline [896]
Agonists	dexmedetomidine (Partial agonist) [896, 1228, 1552, 1573], clonidine (Partial agonist) [896, 1552, 1573], brimonidine [896, 1228, 1552, 1573], apraclonidine [1399], guanabenz [71], guanfacine (Partial agonist) [896, 1231]	dexmedetomidine [896, 1228, 1552, 1573], clonidine (Partial agonist) [896, 1552, 1573], brimonidine (Partial agonist) [896, 1552, 1573], guanabenz [71], guanfacine [896]	dexmedetomidine [896, 1552, 1573], brimonidine (Partial agonist) [896, 1228, 1552, 1573], apraclonidine [1399], guanfacine (Partial agonist) [896], guanabenz [71]
Selective agonists	oxymetazoline (Partial agonist) [896, 1228, 1998]	–	–
Antagonists	yohimbine (p*K*_i_ 8.4–9.2) [255, 440, 1998]	yohimbine (p*K*_i_ 7.9–8.9) [255, 440, 1998], phenoxybenzamine (p*K*_i_ 8.5) [2088], tolazoline (p*K*_i_ 5.5) [896]	yohimbine (p*K*_i_ 8.5–9.5) [255, 440, 1998], WB 4101 (p*K*_i_ 8.4–9.4) [255, 440, 1998], spiroxatrine (p*K*_i_ 9) [1998], mirtazapine (p*K*_i_ 7.7) [539], tolazoline (p*K*_i_ 5.4) [896]
Selective antagonists	BRL 44408 (p*K*_i_ 8.2–8.8) [1998, 2194]	imiloxan (p*K*_i_ 7.3) [1329] – Rat	JP1302 (p*K*_B_ 7.8) [1704]
Labelled ligands	–	–	[^3^ H]MK‐912 (Antagonist) (p*K*_d_ 10.1) [1998]

Adrenoceptors, *β*

*β*‐Adrenoceptors are activated by the endogenous agonists (‐)‐adrenaline and (‐)‐noradrenaline. Isoprenaline is selective for *β*‐adrenoceptors relative to *α*_1_‐ and *α*_2_‐adrenoceptors, while propranolol(pK_i_ 8.2‐9.2) and cyanopindolol(pK_i_ 10.0‐11.0) are relatively *β*_1_ and *β*_2_ adrenoceptor‐selective antagonists. (‐)‐noradrenaline, xamoterol and (‐)‐Ro 363 show selectivity for *β*_1_‐ relative to *β*_2_‐adrenoceptors. Pharmacological differences exist between human and mouse *β*_3_‐adrenoceptors, and the 'rodent selective' agonists BRL 37344 and CL316243 have low efficacy at the human *β*_3_‐adrenoceptor whereas CGP 12177 and L 755507 activate human *β*_3_‐adrenoceptors [88]. *β*_3_‐Adrenoceptors are resistant to blockade by propranolol, but can be blocked by high concentrations of bupranolol. SR59230A has reasonably high affinity at *β*_3_‐adrenoceptors, but does not discriminate well between the three *β*‐ subtypes whereas L 755507 is more selective. [^125^I]‐cyanopindolol, [^125^I]‐hydroxy benzylpindolol and [^3^H]‐alprenolol are high affinity radioligands that label *β*_1_‐ and *β*_2_‐ adrenoceptors and *β*_3_‐adrenoceptors can be labelled with higher concentrations (nM) of [^125^I]‐cyanopindolol together with *β*_1_‐ and *β*_2_‐adrenoceptor antagonists. [^3^H]‐L‐748337 is a *β*_3_‐selective radioligand [2020]. Fluorescent ligands such as BODIPY‐TMR‐CGP12177 can be used to track *β*‐adrenoceptors at the cellular level [8]. Somewhat selective *β*_1_‐adrenoceptor agonists (denopamine, dobutamine) are used short term to treat cardiogenic shock but, chronically, reduce survival. *β*_1_‐Adrenoceptor‐preferring antagonists are used to treat hypertension (atenolol, betaxolol, bisoprolol, metoprolol and nebivolol), cardiac arrhythmias (atenolol, bisoprolol, esmolol) and cardiac failure (metoprolol, nebivolol). Cardiac failure is also treated with carvedilol that blocks *β*_1_‐ and *β*_2_‐adrenoceptors, as well as *α*_1_‐adrenoceptors. Short (salbutamol, terbutaline) and long (formoterol, salmeterol) acting *β*_2_‐adrenoceptor‐selective agonists are powerful bronchodilators used to treat respiratory disorders. Many first generation *β*‐adrenoceptor antagonists (propranolol) block both *β*_1_‐ and *β*_2_‐adrenoceptors and there are no *β*_2_‐adrenoceptor‐selective antagonists used therapeutically. The *β*_3_‐adrenoceptor agonist mirabegron is used to control overactive bladder syndrome.

**Table:** 

Nomenclature	*β*_1_ ‐adrenoceptor	*β*_2_ ‐adrenoceptor	*β*_3_ ‐adrenoceptor
HGNC, UniProt	ADRB1, P08588	ADRB2, P07550	ADRB3, P13945
Potency order of endogenous ligands	(‐)‐noradrenaline>(‐)‐adrenaline	(‐)‐adrenaline>(‐)‐noradrenaline	(‐)‐noradrenaline = (‐)‐adrenaline
Endogenous agonists	(‐)‐adrenaline [579, 806], (‐)‐noradrenaline [579, 806], noradrenaline [579]	(‐)‐adrenaline [579, 806, 893], (‐)‐noradrenaline [579, 806]	(‐)‐noradrenaline [806, 1589, 1880], (‐)‐adrenaline [806]
Agonists	pindolol (Partial agonist) [1058], isoprenaline [579, 1723], dobutamine (Partial agonist) [870]	pindolol (Partial agonist) [1058], arformoterol [37], isoprenaline [1723], dobutamine (Partial agonist) [1166], ephedrine (Partial agonist) [893]	carazolol [1318]
Selective agonists	(‐)‐Ro 363 [1355], xamoterol (Partial agonist) [870], denopamine (Partial agonist) [870, 1903]	formoterol [85], salmeterol [85], zinterol [85], vilanterol [1605], procaterol [85], indacaterol [114], fenoterol [59], salbutamol (Partial agonist) [87, 870], terbutaline (Partial agonist) [87], orciprenaline [1853]	L 755507 [85], L742791 [2086], mirabegron [1922], CGP 12177 (Partial agonist) [163, 1210, 1318, 1355], SB251023 [850] – Mouse, BRL 37344 [163, 456, 806, 1318], CL316243 [2168]
Antagonists	carvedilol (p*K*_i_ 9.5) [272], bupranolol (p*K*_i_ 7.3–9) [272, 1210], levobunolol (p*K*_i_ 8.4) [71], labetalol (p*K*_i_ 8.2) [71], metoprolol (p*K*_i_ 7–7.6) [87, 272, 806, 1210], esmolol (p*K*_i_ 6.9) [71], nadolol (p*K*_i_ 6.9) [272], practolol (p*K*_i_ 6.1–6.8) [87, 1210], propafenone (p*K*_i_ 6.7) [71], sotalol (p*K*_i_ 6.1) [71]	carvedilol (p*K*_i_ 9.4–9.9) [87, 272], timolol (p*K*_i_ 9.7) [87], propranolol (p*K*_i_ 9.1–9.5) [87, 90, 870, 1210], levobunolol (p*K*_i_ 9.3) [71], bupranolol (p*K*_i_ 8.3–9.1) [272, 1210], alprenolol (p*K*_i_ 9) [87], nadolol (p*K*_i_ 7–8.6) [87, 272], labetalol (p*K*_i_ 8) [71], propafenone (p*K*_i_ 7.4) [71], sotalol (p*K*_i_ 6.5) [71]	carvedilol (p*K*_i_ 9.4) [272], SR59230A (p*K*_i_ 6.9–8.4) [272, 430, 806], bupranolol (p*K*_i_ 6.8–7.3) [163, 272, 1210, 1318], propranolol (p*K*_i_ 6.3–7.2) [1210, 1589], levobunolol (p*K*_i_ 6.8) [1589]
Selective antagonists	CGP 20712A (p*K*_i_ 8.5–9.2) [87, 272, 1210], levobetaxolol (p*K*_i_ 9.1) [1785], betaxolol (p*K*_i_ 8.8) [1210], nebivolol (pIC_50_ 8.1–8.7) [1543] – Rabbit, atenolol (p*K*_i_ 6.7–7.6) [87, 928, 1210], acebutolol (p*K*_i_ 6.4) [71]	ICI 118551 (Inverse agonist) (p*K*_i_ 9.2–9.5) [87, 90, 1210]	L‐748337 (p*K*_i_ 8.4) [272], L748328 (p*K*_i_ 8.4) [272]
Labelled ligands	[^125^ I]ICYP (Selective Antagonist) (p*K*_d_ 10.4–11.3) [870, 1210, 1723]	[^125^ I]ICYP (Antagonist) (p*K*_d_ 11.1) [1210, 1723]	[^125^ I]ICYP (Agonist, Partial agonist) [1210, 1355, 1589, 1723, 1880]
Comments	The agonists indicated have less than two orders of magnitude selectivity [85].	–	Agonist SB251023 has a pEC_50_ of 6.9 for the splice variant of the mouse *β*_3_ receptor, *β*_3b_ [850].

### Comments

Adrenoceptors, *α*_1_

The *α*_1C_‐adrenoceptor corresponds to the pharmacologically defined *α*_1A_‐adrenoceptor [789]. Some tissues possess *α*_1A_‐adrenoceptors (*α*_1L_‐adrenoceptors [559, 1382]) that display relatively low affinity in functional and binding assays for prazosin indicative of different receptor states or locations. *α*_1A_‐adrenoceptor C‐terminal splice variants form homo‐ and heterodimers, but fail to generate a functional *α*_1L_‐adrenoceptor [1628]. *α*_1D_‐Adrenoceptors form heterodimers with *α*_1B_‐ or *β*_2_‐adrenoceptors that show increased cell‐surface expression [1993]. Recombinant *α*_1D_‐adrenoceptors have been shown in some heterologous systems to be mainly located intracellularly but cell‐surface localization is encouraged by truncation of the N‐terminus, or by co‐expression of *α*_1B_‐ or *β*_2_‐adrenoceptors [706, 1993]. In blood vessels all three *α*_1‐_adrenoceptor subtypes are located on the surface and intracellularly [1320, 1321]. Signalling is predominantly via G_q/11_ but *α*_1_‐adrenoceptors also couple to G_i/o_, G_s_ and G_12/13_. Several *α*_1A_‐adrenoceptor agonists display ligand directed signalling bias relative to noradrenaline [521]. There are also differences between subtypes in coupling efficiency to different pathways. In vascular smooth muscle, the potency of agonists is related to the predominant subtype, *α*_1D_‐ conveying greater agonist sensitivity than *α*_1A_‐adrenoceptors [553].

Adrenoceptors, *α*_2_

ARC‐239 and prazosin show selectivity for *α*_2B_‐ and *α*_2C_‐adrenoceptors over *α*_2A_‐adrenoceptors.Oxymetazoline is a reduced efficacy imidazoline agonist but also binds to non‐GPCR binding sites for imidazolines, classified as I_1_, I_2_ and I_3_sites [406]; catecholamines have a low affinity, while rilmenidine and moxonidine are selective ligands evoking hypotensive effects in vivo. I_1_‐imidazoline receptors cause central inhibition of sympathetic tone, I_2_‐imidazoline receptors are an allosteric binding site on monoamine oxidase B, and I_3_‐imidazoline receptors regulate insulin secretion from pancreatic *β*‐cells. *α*_2A_‐adrenoceptor stimulation reduces insulin secretion from *β*‐islets [2171], with a polymorphism in the 5'‐UTR of the ADRA2A gene being associated with increased receptor expression in *β*‐islets and heightened susceptibility to diabetes [1673]. *α*_2A_‐ and *α*_2C_‐adrenoceptors form homodimers [1829]. Heterodimers between *α*_2A_‐ and either the *α*_2c_‐adrenoceptor or *μ* opioid peptide receptor exhibit altered signalling and trafficking properties compared to the individual receptors [1829, 1931, 2036]. Signalling by *α*_2_‐adrenoceptors is primarily via G_i/o_, although the *α*_2A_‐adrenoceptor also couples to G_s_[487]. Imidazoline compounds display bias relative to each other at the *α*_2A_‐adrenoceptor [1544]. The noradrenaline reuptake inhibitor desipramine acts directly on the *α*_2A_‐adrenoceptor to promote internalisation via recruitment of arrestin [385].

Adrenoceptors, *β*

[^125^ I]ICYP can be used to define *β*_1_‐ or *β*_2_‐adrenoceptors when conducted in the presence of a *β*_1_‐ or *β*_2_‐adrenoceptor‐selective antagonist. A fluorescent analogue of CGP 12177 can be used to study *β*_2_‐adrenoceptors in living cells [88]. [^125^ I]ICYP at higher (nM) concentrations can be used to label *β*_3_‐adrenoceptors in systems with few if any other *β*‐adrenoceptor subtypes. The *β*_3_‐adrenoceptor has an intron in the coding region, but splice variants have only been described for the mouse [522], where the isoforms display different signalling characteristics [850]. There are 3 *β*‐adrenoceptors in turkey (termed the t*β*, t*β*3c and t*β*4c) that have a pharmacology that differs from the human *β*‐adrenoceptors [86]. Numerous polymorphisms have been described for the *β*‐adrenoceptors; some are associated with signalling and trafficking, altered susceptibility to disease and/or altered responses to pharmacotherapy [1169]. All *β*‐adrenoceptors couple to G_s_(activating adenylyl cyclase and elevating cAMP levels), but also activate G_i_ and *β*‐arrestin‐mediated signalling. Many *β*_1_‐ and *β*_2_‐adrenoceptor antagonists are agonists at *β*_3_‐adrenoceptors (CL316243, CGP 12177 and carazolol). Many ‘antagonists’ of cAMP accumulation, for example carvedilol and bucindolol, weakly activate MAP kinase pathways [89, 523, 589, 590, 1721, 1722] and thus display 'protean agonism'. Bupranolol acts as a neutral antagonist in most systems so far examined. Agonists also display biased signalling at the *β*_2_‐adrenoceptor via G_s_ or arrestins [470]. X‐ray crystal structures have been described of the agonist bound [2075] and antagonist bound forms of the *β*_1_‐ [2076], agonist‐bound [328] and antagonist‐bound forms of the *β*_2_‐adrenoceptor [1632, 1672], as well as a fully active agonist‐bound, G_s_ protein‐coupled *β*_2_‐adrenoceptor [1633]. Carvedilol and bucindolol bind to a site on the *β*_1_‐adrenoceptor involving contacts in TM2, 3, and 7 and extracellular loop 2 that may facilitate coupling to arrestins [2076]. Compounds displaying arrestin‐biased signalling at the *β*_2_‐adrenoceptor have a greater effect on the conformation of TM7, whereas full agonists for G_s_ coupling promote movement of TM5 and TM6 [1192]. Recent studies using NMR spectroscopy demonstrate significant conformational flexibility in the *β*_2_‐adrenoceptor that is stabilized by both agonist and G proteins highlighting the dynamic nature of interactions with both ligand and downstream signalling partners [992, 1260, 1479]. Such flexibility likely has consequences for our understanding of biased agonism, and for the future therapeutic exploitation of this phenomenon.

### Further reading on Adrenoceptors

Baker JG et al. (2011) Evolution of *β*‐blockers: from anti‐anginal drugs to ligand‐directed signalling. Trends Pharmacol. Sci. 32: 227‐34 [PMID:21429598]

Bylund DB et al. (1994) International Union of Pharmacology nomenclature of adrenoceptors. Pharmacol. Rev. 46: 121‐136 [PMID:7938162]

Evans BA et al. (2010) Ligand‐directed signalling at beta‐adrenoceptors. Br. J. Pharmacol. 159: 1022‐38 [PMID:20132209]

Jensen BC et al. (2011) Alpha‐1‐adrenergic receptors: targets for agonist drugs to treat heart failure. J. Mol. Cell. Cardiol. 51: 518‐28 [PMID:21118696]

Kobilka BK. (2011) Structural insights into adrenergic receptor function and pharmacology. Trends Pharmacol. Sci. 32: 213‐8 [PMID:21414670]

Langer SZ. (2015) *α*2‐Adrenoceptors in the treatment of major neuropsychiatric disorders. Trends Pharmacol. Sci. 36: 196‐202 [PMID:25771972]

Michel MC et al. (2015) Selectivity of pharmacological tools: implications for use in cell physiology. A review in the theme: Cell signaling: proteins, pathways and mechanisms. Am. J. Physiol., Cell Physiol. 308: C505‐20 [PMID:25631871]

## Angiotensin receptors

### Overview

The actions of angiotensin II (AGT, P01019) (Ang II) are mediated by AT_1_ and AT_2_ receptors (nomenclature as agreed by the NC‐IUPHAR Subcommittee on Angiotensin receptors [423, 950]), which have around 30% sequence similarity. The decapeptide angiotensin I (AGT, P01019), the octapeptide angiotensin II(AGT, P01019) and the heptapeptide angiotensin III(AGT, P01019) are endogenous ligands. Losartan, candesartan, telmisartan, etc. are clinically used AT_1_ receptor blockers.

**Table:** 

Nomenclature	AT_1_ receptor	AT_2_ receptor
HGNC, UniProt	AGTR1, P30556	AGTR2, P50052
Endogenous agonists	angiotensin II (AGT, P01019) [425, 2021], angiotensin III (AGT, P01019) [425]	angiotensin III (AGT, P01019) [394, 425, 2105], angiotensin II (AGT, P01019) [425, 1838, 2105], angiotensin‐(1‐7) (AGT, P01019) [194]
Selective agonists	L‐162,313 [1559]	CGP42112 [194], [p‐aminoPhe6]ang II [425, 1860] – Rat
Antagonists	telmisartan (pIC_50_ 8.4) [1303], olmesartan (pIC_50_ 8.1) [1027]	–
Selective antagonists	candesartan (pIC_50_ 9.5–9.7) [2021], EXP3174 (pIC_50_ 7.4–9.5) [1965, 2021], eprosartan (pIC_50_ 8.4–8.8) [492], irbesartan (pIC_50_ 8.7–8.8) [2021], losartan (pIC_50_ 7.4–8.7) [425, 1965], valsartan (pIC_50_ 8.6) [424], azilsartan (pIC_50_ 8.1–8.1) [1623, 1917]	PD123177 (pIC_50_ 8.5–9.5) [305, 336, 478] – Rat, EMA401 (pIC_50_ 8.5–9.3) [543, 1656, 1836], PD123319 (p*K*_d_ 8.7–9.2) [425, 477, 2115]
Labelled ligands	[^3^ H]A81988 (Antagonist) (p*K*_d_ 9.2) [725] – Rat, [^3^ H]L158809 (Antagonist) (p*K*_d_ 9.2) [320] – Rat, [^3^ H]eprosartan (Antagonist) (p*K*_d_ 9.1) [22] – Rat, [^3^ H]valsartan (Antagonist) (pIC_50_ 8.8–9) [2034], [^125^ I]EXP985 (Antagonist) (p*K*_d_ 8.8) [337] – Rat, [^3^ H]losartan (Antagonist) (p*K*_d_ 8.2) [309] – Rat	[^125^ I]CGP42112 (Agonist) [425, 2105, 2106]
Comments	telmisartan and candesartan are also reported to be agonists of PPAR*γ* [1877].	–

### Comments

AT_1_ receptors are predominantly coupled to G_q/11_, however they are also linked to arrestin recruitment and stimulate G protein‐independent arrestin signalling [1221]. Most species express a single AGTR1 gene, but two related agtr1a and agtr1b receptor genes are expressed in rodents. The AT_2_ receptor counteracts several of the growth responses initiated by the AT_1_ receptors. The AT_2_ receptor is much less abundant than the AT_1_ receptor in adult tissues and is upregulated in pathological conditions. AT_1_ receptor antagonists bearing substituted 4‐phenylquinoline moieties have been synthesized, which bind to AT_1_ receptors with nanomolar affinity and are slightly more potent than losartan in functional studies [275]. The antagonist activity of CGP42112 at the AT_2_ receptor has also been reported [1469]. The AT_1_ and bradykinin B_2_ receptors have been proposed to form a heterodimeric complex [3]. There is also evidence for an AT_4_ receptor that specifically binds angiotensin IV (AGT, P01019) and is located in the brain and kidney. An additional putative endogenous ligand for the AT_4_ receptor has been described (LVV‐hemorphin(HBB, P68871), a globin decapeptide) [1351].

### .1 Further reading on Angiotensin receptors

de Gasparo M et al. (2000) International Union of Pharmacology. XXIII. The angiotensin II receptors. Pharmacol. Rev. 52: 415‐472 [PMID:10977869]

Karnik SS et al. (2015) International Union of Basic and Clinical Pharmacology. XCIX. Angiotensin Receptors: Interpreters of Pathophysiological Angiotensinergic Stimuli [corrected]. Pharmacol. Rev. 67: 754‐819 [PMID:26315714]

Zhang H et al. (2015) Structural Basis for Ligand Recognition and Functional Selectivity at Angiotensin Receptor. J. Biol. Chem. 290: 29127‐39 [PMID:26420482]

Zhang H et al. (2015) Structure of the Angiotensin receptor revealed by serial femtosecond crystallography. Cell 161: 833‐44 [PMID:25913193]

## Apelin receptor

### Overview

The apelin receptor (nomenclature as agreed by the NC‐IUPHAR Subcommittee on the apelin receptor [1582]) responds to apelin, a 36 amino‐acid peptide derived initially from bovine stomach. Apelin‐36(APLN, Q9ULZ1), apelin‐13(APLN, Q9ULZ1) and [Pyr^1^ ]apelin‐13(APLN, Q9ULZ1) are the predominant endogenous ligands which are cleaved from a 77 amino‐acid precursor peptide (APLN, Q9ULZ1) by a so far unidentified enzymatic pathway [1938]. A second family of peptides discovered independently and named Elabela [338] or Toddler, that has little sequence similarity to apelin, has been proposed as a second endogenous apelin receptor ligand [1542]. Structure‐activity relationship Elabela analogues have been decsribed [1406].

**Table:** 

Nomenclature	apelin receptor
HGNC, UniProt	APLNR, P35414
Potency order of endogenous ligands	[Pyr^1^ ]apelin‐13 (APLN, Q9ULZ1) ≥apelin‐13 (APLN, Q9ULZ1) >apelin‐36 (APLN, Q9ULZ1) [529, 1938]
Endogenous agonists	apelin‐13 (APLN, Q9ULZ1) [529, 824, 1315], apelin receptor early endogenous ligand (APELA, P0DMC3) [436], apelin‐17 (APLN, Q9ULZ1) [496, 1315], [Pyr^1^]apelin‐13 (APLN, Q9ULZ1) [961, 1315], Elabela/Toddler‐21 (APELA, P0DMC3) [2174], Elabela/Toddler‐32 (APELA, P0DMC3) [2174], apelin‐36 (APLN, Q9ULZ1) [529, 824, 961, 1315], Elabela/Toddler‐11 (APELA, P0DMC3) [2174]
Selective agonists	CMF‐019 (Biased agonist) [1639], MM07 (Biased agonist) [209]
Antagonists	MM54 (p*K*_i_ 8.2) [1227]
Labelled ligands	[^125^I][Nle^75^,Tyr^77^ ]apelin‐36 (human) (Agonist) [961], [^125^I][Glp^65^Nle^75^,Tyr^77^ ]apelin‐13 (Agonist) [824], [^125^I](Pyr^1^)apelin‐13 (Agonist) [955], [^125^ I]apelin‐13 (Agonist) [529], [^3^H](Pyr^1^)[Met(0)11]‐apelin‐13 (Agonist) [1315]

### Comments

Potency order determined for heterologously expressed human apelin receptor (pD_2_ values range from 9.5 to 8.6). The apelin receptor may also act as a co‐receptor with CD4 for isolates of human immunodeficiency virus, with apelin blocking this function [293]. A modified apelin‐13 peptide, apelin‐13(F13A) was reported to block the hypotensive response to apelin in rat in vivo [1132], however, this peptide exhibits agonist activity in HEK293 cells stably expressing the recombinant apelin receptor [529].

### Further reading on Apelin receptor

Cheng B et al. (2012) Neuroprotection of apelin and its signaling pathway. Peptides 37: 171‐3 [PMID:22820556]

Langelaan DN et al. (2009) Structural insight into G‐protein coupled receptor binding by apelin. Biochemistry 48: 537‐48 [PMID:19123778]

O'Carroll AM et al. (2013) The apelin receptor APJ: journey from an orphan to a multifaceted regulator of homeostasis. J. Endocrinol. 219: R13‐35 [PMID:23943882]

Pitkin SL et al. (2010) International Union of Basic and Clinical Pharmacology. LXXIV. Apelin receptor nomenclature, distribution, pharmacology, and function. Pharmacol. Rev. 62: 331‐42 [PMID:20605969]

Yang P et al. (2015) Apelin, Elabela/Toddler, and biased agonists as novel therapeutic agents in the cardiovascular system. Trends Pharmacol. Sci. [PMID:26143239]

## Bile acid receptor

### Overview

The bile acid receptor (GPBA) responds to bile acids produced during the liver metabolism of cholesterol. Selective agonists are promising drugs for the treatment of metabolic disorders, such as type II diabetes, obesity and atherosclerosis.

**Table:** 

Nomenclature	GPBA receptor
HGNC, UniProt	GPBAR1, Q8TDU6
Potency order of endogenous ligands	lithocholic acid>deoxycholic acid>chenodeoxycholic acid, cholic acid [960, 1278]
Selective agonists	S‐EMCA [1550] – Mouse, betulinic acid [621], oleanolic acid [1720]

### Comments

The triterpenoid natural product betulinic acid has also been reported to inhibit inflammatory signalling through the NF*κ*B pathway [1916]. Disruption of GPBA expression is reported to protect from cholesterol gallstone formation [2031]. A new series of 5‐phenoxy‐1,3‐dimethyl‐1H‐pyrazole‐4‐carboxamides have been reported as highly potent agonists [1204].

### Further reading on Bile acid receptor

Duboc H et al. (2014) The bile acid TGR5 membrane receptor: from basic research to clinical application. Dig Liver Dis 46: 302‐12 [PMID:24411485]

Lefebvre P et al. (2009) Role of bile acids and bile acid receptors in metabolic regulation. Physiol. Rev. 89: 147‐91 [PMID:19126757]

Lieu T et al. (2014) GPBA: a GPCR for bile acids and an emerging therapeutic target for disorders of digestion and sensation. Br. J. Pharmacol. 171: 1156‐66 [PMID:24111923]

van Nierop FS et al. (2016) Clinical relevance of the bile acid receptor TGR5 in metabolism. Lancet Diabetes Endocrinol [PMID:27639537]

## Bombesin receptors

### Overview

Mammalian bombesin (Bn) receptors comprise 3 subtypes: BB_1_, BB_2_, BB_3_(nomenclature recommended by the NC‐IUPHAR Subcommittee on bombesin receptors, [900]). BB_1_ and BB_2_ are activated by the endogenous ligands gastrin‐releasing peptide(GRP, P07492) (GRP), neuromedin B(NMB, P08949) (NMB) and GRP‐(18‐27)(GRP, P07492) (previously named neuromedin C). Bombesin is a tetradecapeptide, originally derived from amphibians. The three Bn receptor subtypes couple primarily to the G_q/11_ and G_12/13_ family of G proteins [900] (but see also [908, 1995]). Each of these receptors is widely distributed in the CNS and peripheral tissues [659, 900, 1590, 1626, 1626, 1715, 1715, 2208]. Activation of BB_1_ and BB_2_ receptors causes a wide range of physiological actions, including the stimulation of normal and neoplastic tissue growth, smooth‐muscle contraction, appetite and feeding behavior, secretion and many central nervous system effects [900, 901, 902, 1248, 1371, 1626, 1626]. A physiological role for the BB_3_ receptor has yet to be fully defined although recently studies using receptor knockout mice and newly described agonists/antagonists suggest an important role in glucose and insulin regulation, metabolic homeostasis, feeding, regulation of body temperature and other CNS behaviors, obesity, diabetes mellitus and growth of normal/neoplastic tissues [659, 1249, 1249, 1496, 1496, 2145].

**Table:** 

Nomenclature	BB_1_ receptor	BB_2_ receptor	BB_3_ receptor
HGNC, UniProt	NMBR, P28336	GRPR, P30550	BRS3, P32247
Endogenous agonists	neuromedin B (NMB, P08949) [900, 1626, 1995]	neuromedin C [1995], gastrin releasing peptide(14‐27) (human) [1995]	–
Selective agonists	–	–	compound 8a [1194], compound 9g [1284], MK‐7725 [339], MK‐5046 [1375, 1759], [D‐Tyr^6^,Apa‐4Cl^11^,Phe^13^,Nle^14^]bombesin‐(6‐14) [1263], compound 17c [1283], compound 9f [1284], bag‐1 [692], compound 22e [761], bag‐2 [692]
Antagonists	D‐Nal‐Cys‐Tyr‐D‐Trp‐Lys‐Val‐Cys‐Nal‐NH_2_ (pIC_50_ 6.2–6.6) [658]	–	–
Selective antagonists	PD 176252 (pIC_50_ 9.3–9.8) [658], PD 168368 (pIC_50_ 9.3–9.6) [658], dNal‐cyc(Cys‐Tyr‐dTrp‐Orn‐Val)‐Nal‐NH_2_	[D‐Phe^6^, Leu^13^, Cpa^14^,*ψ* 13‐14]bombesin‐(6‐14) (p*K*_i_ 9.8) [658], JMV641 (pIC_50_ 9.3) [1970] – Mouse, [(3‐Ph‐Pr ^6^), His^7^,D‐Ala^11^,D‐Pro^13^,*ψ*13‐14),Phe^14^ ]bombesin‐(6‐14) (pIC_50_ 9.2) [658, 1125], JMV594 (pIC_50_ 8.9) [1199, 1970] – Mouse, [D‐Tpi^6^, Leu^13^ *ψ*(CH_2_NH)‐Leu^14^]bombesin‐(6‐14) (pIC_50_ 8.9) [658], Ac‐GRP‐(20‐26)‐methylester (pIC_50_ 8.7) [658]	bantag‐1 (pIC_50_ 8.6–8.7) [692, 1375], ML‐18 (pIC_50_ 5.3) [1370]
Labelled ligands	[^125^ I]BH‐NMB (human, mouse, rat) (Agonist), [^125^I][Tyr^4^ ]bombesin (Agonist)	[^125^I][D‐Tyr^6^ ]bombesin‐(6‐13)‐methyl ester (Selective Antagonist) (p*K*_d_ 9.3) [1262] – Mouse, [^125^I][Tyr^4^ ]bombesin (Agonist) [135], [^125^ I]GRP (human) (Agonist)	[^3^ H]bag‐2 (Agonist) [692] – Mouse, [^125^I][D‐Tyr^6^,*β*‐Ala^11^,Phe^13^,Nle^14^ ]bombesin‐(6‐14) (Agonist) [1264, 1375]

### Comments

All three human subtypes may be activated by [D‐Phe^6^,*β*‐Ala^11^,Phe^13^,Nle^14^ ]bombesin‐(6‐14)[1264]. [D‐Tyr^6^,Apa‐4Cl^11^,Phe^13^,Nle^14^ ]bombesin‐(6‐14) has more than 200‐fold selectivity for BB_3_ receptors over BB_1_ and BB_2_[1263, 1264, 1626, 1627].

### Further reading on Bombesin receptors

Ferreira CA et al. (2017) Radiolabeled bombesin derivatives for preclinical oncological imaging. Biomed. Pharmacother. 87: 58‐72 [PMID:28040598]

González N et al. (2015) Bombesin receptor subtype 3 as a potential target for obesity and diabetes. Expert Opin. Ther. Targets 1‐18 [PMID:26066663]

Jensen RT et al. (2008) International Union of Pharmacology. LXVIII. Mammalian bombesin receptors: nomenclature, distribution, pharmacology, signaling, and functions in normal and disease states. Pharmacol. Rev. 60: 1‐42 [PMID:18055507]

Jensen RT et al. (2013) Bombesin Peptides (Cancer). In Handbook of Biologically Active Peptides. 2nd Revised edition. Edited by Kastin AJ: Elsevier: 506‐511 [ISBN: 9780123850959]

Ramos‐Álvarez I et al. (2015) Insights into bombesin receptors and ligands: Highlighting recent advances. Peptides [PMID:25976083]

Sayegh AI. (2013) The role of bombesin and bombesin‐related peptides in the short‐term control of food intake. Prog Mol Biol Transl Sci 114: 343‐70 [PMID:23317790]

## Bradykinin receptors

### Overview

Bradykinin (or kinin) receptors (nomenclature as agreed by the NC‐IUPHAR subcommittee on Bradykinin (kinin) Receptors [1136]) are activated by the endogenous peptides bradykinin(KNG1, P01042) (BK), [des‐Arg ^9^]bradykinin(KNG1, P01042), Lys‐BK (kallidin(KNG1, P01042)), [des‐Arg ^10^]kallidin(KNG1, P01042), T‐kinin(KNG1, P01042) (Ile‐Ser‐BK), [Hyp ^3^]bradykinin(KNG1, P01042) and Lys‐[Hyp ^3^]‐bradykinin(KNG1, P01042). The variation in affinity or inactivity of B_2_ receptor antagonists could reflect the existence of species homologues of B_2_ receptors.

**Table:** 

Nomenclature	B_1_ receptor	B_2_ receptor
HGNC, UniProt	BDKRB1, P46663	BDKRB2, P30411
Potency order of endogenous ligands	[des‐Arg ^10^]kallidin (KNG1, P01042) >[des‐Arg ^9^]bradykinin (KNG1, P01042) = kallidin (KNG1, P01042) >bradykinin (KNG1, P01042)	kallidin (KNG1, P01042) >bradykinin (KNG1, P01042) ≫[des‐Arg ^9^]bradykinin (KNG1, P01042), [des‐Arg ^10^]kallidin (KNG1, P01042)
Endogenous agonists	[des‐Arg ^10^]kallidin (KNG1, P01042) [72, 110, 919]	–
Selective agonists	[Sar,D‐Phe ^8^,des‐Arg^9^]bradykinin [919]	[Hyp ^3^,Tyr(Me)^8^]BK, [Phe ^8^,*ψ*(CH_2_‐NH)Arg^9^]BK
Antagonists	[Leu ^9^,des‐Arg^10^]kallidin (p*K*_i_ 9.1–9.3) [72, 110]	–
Selective antagonists	B‐9958 (p*K*_i_ 9.2–10.3) [630, 1642], R‐914 (pA_2_ 8.6) [650], R‐715 (pA_2_ 8.5) [651]	icatibant (p*K*_i_ 10.2) [39], FR173657 (pA_2_ 8.2) [1666], anatibant (p*K*_i_ 8.2) [1608]
Labelled ligands	[^125^ I]Hpp‐desArg ^10^HOE140 (p*K*_d_ 10), [^3^ H]Lys‐[des‐Arg ^9^]BK (Agonist), [^3^ H]Lys‐[Leu ^8^][des‐Arg^9^]BK (Antagonist)	[^3^ H]BK (human, mouse, rat) (Agonist) [2123] – Mouse, [^3^ H]NPC17731 (Antagonist) (p*K*_d_ 9.1–9.4) [2211, 2212], [^125^ I][Tyr ^8^]bradykinin (Agonist)

### Further reading on Bradykinin receptors

Campos MM et al. (2006) Non‐peptide antagonists for kinin B1 receptors: new insights into their therapeutic potential for the management of inflammation and pain. Trends Pharmacol. Sci. 27: 646‐51 [PMID:17056130]

Duchene J et al. (2009) The kinin B(1) receptor and inflammation: new therapeutic target for cardiovascular disease. Curr Opin Pharmacol 9: 125‐31 [PMID:19124274]

Marceau F et al. (2004) Bradykinin receptor ligands: therapeutic perspectives. Nat Rev Drug Discov 3: 845‐52 [PMID:15459675]

Paquet JL et al. (1999) Pharmacological characterization of the bradykinin B2 receptor: inter‐species variability and dissociation between binding and functional responses. Br. J. Pharmacol. 126: 1083‐90 [PMID:10204994]

Thornton E et al. (2010) Kinin receptor antagonists as potential neuroprotective agents in central nervous system injury. Molecules 15: 6598‐618 [PMID:20877247]

## Calcitonin receptors

### Overview

This receptor family comprises a group of receptors for the calcitonin/CGRP family of peptides. The calcitonin (CT), amylin (AMY), calcitonin gene‐related peptide (CGRP) and adrenomedullin (AM) receptors (nomenclature as agreed by the NC‐IUPHAR Subcommittee on CGRP, AM, AMY, and CT receptors [755, 1600]) are generated by the genes CALCR (which codes for the CT receptor) and CALCRL(which codes for the calcitonin receptor‐like receptor, CLR, previously known as CRLR). Their function and pharmacology are altered in the presence of RAMPs (receptor activity‐modifying proteins), which are single TM domain proteins of ca. 130 amino acids, identified as a family of three members; RAMP1, RAMP2 and RAMP3. There are splice variants of the CT receptor; these in turn produce variants of the AMY receptor [1600], some of which can be potently activated by CGRP. The endogenous agonists are the peptides calcitonin(CALCA, P01258), *α* ‐CGRP(CALCA, P06881) (formerly known as CGRP‐I), *β* ‐CGRP(CALCB, P10092) (formerly known as CGRP‐II), amylin(IAPP, P10997) (occasionally called islet‐amyloid polypeptide, diabetes‐associated polypeptide), adrenomedullin(ADM, P35318) and adrenomedullin 2/intermedin (ADM2, Q7Z4H4). There are species differences in peptide sequences, particularly for the CTs. CTR‐stimulating peptide {Pig} (CRSP) is another member of the family with selectivity for the CT receptor but it is not expressed in humans [952]. Olcegepant (also known as BIBN4096BS, pKi ˜ 10.5) and telcagepant (also known as MK0974, pKi ˜ 9) are the most selective antagonists available, showing selectivity for CGRP receptors, with a particular preference for those of primate origin.

CLR (calcitonin receptor‐like receptor) by itself binds no known endogenous ligand, but in the presence of RAMPs it gives receptors for CGRP, adrenomedullin and adrenomedullin 2/intermedin.

**Table:** 

Nomenclature	CT receptor	AMY_1_ receptor	AMY_2_ receptor	AMY_3_ receptor
HGNC, UniProt	CALCR, P30988	–	–	–
Subunits	–	CT receptor, RAMP1 (Accessory protein)	CT receptor, RAMP2 (Accessory protein)	CT receptor, RAMP3 (Accessory protein)
Potency order of endogenous ligands	calcitonin (salmon)≥calcitonin (CALCA, P01258) ≥amylin (IAPP, P10997), *α* ‐CGRP (CALCA, P06881) >adrenomedullin (ADM, P35318), adrenomedullin 2/intermedin (ADM2, Q7Z4H4)	calcitonin (salmon)≥amylin (IAPP, P10997) ≥*α* ‐CGRP (CALCA, P06881) >adrenomedullin 2/intermedin (ADM2, Q7Z4H4) ≥calcitonin (CALCA, P01258) >adrenomedullin (ADM, P35318)	Poorly defined	calcitonin (salmon)≥amylin (IAPP, P10997) >*α* ‐CGRP (CALCA, P06881) ≥adrenomedullin 2/intermedin (ADM2, Q7Z4H4) ≥calcitonin (CALCA, P01258) >adrenomedullin (ADM, P35318)
Endogenous agonists	calcitonin (CALCA, P01258) [32, 62, 752, 1080, 1153, 1396]	*α* ‐CGRP (CALCA, P06881) [752, 1079, 1080, 1153, 2057], amylin (IAPP, P10997) [643]	amylin (IAPP, P10997) [643]	amylin (IAPP, P10997) [643]
Sub/family‐selective agonists	pramlintide [643]	pramlintide [643]	–	pramlintide [643]
Sub/family‐selective antagonists	CT‐(8‐32) (salmon) (p*K*_d_ 9) [793], AC187 (p*K*_i_ 7.2) [752]	AC187 (p*K*_i_ 8) [752], CT‐(8‐32) (salmon) (p*K*_i_ 7.8) [752], olcegepant (p*K*_d_ 7.2) [2057]	–	CT‐(8‐32) (salmon) (p*K*_i_ 7.9) [752], AC187 (p*K*_i_ 7.7) [752]
Labelled ligands	[^125^ I]CT (human) (Agonist), [^125^ I]CT (salmon) (Agonist)	[^125^I]*α* CGRP (human) (Agonist), [^125^ I]BH‐AMY (rat, mouse) (Agonist)	[^125^ I]BH‐AMY (rat, mouse) (Agonist)	[^125^ I]BH‐AMY (rat, mouse) (Agonist)

**Table:** 

Nomenclature	calcitonin receptor‐like receptor	CGRP receptor	AM_1_ receptor	AM_2_ receptor
HGNC, UniProt	CALCRL, Q16602	–	–	–
Subunits	–	calcitonin receptor‐like receptor, RAMP1 (Accessory protein)	calcitonin receptor‐like receptor, RAMP2 (Accessory protein)	calcitonin receptor‐like receptor, RAMP3 (Accessory protein)
Potency order of endogenous ligands	–	*α* ‐CGRP (CALCA, P06881) >adrenomedullin (ADM, P35318) ≥adrenomedullin 2/intermedin (ADM2, Q7Z4H4) >amylin (IAPP, P10997) ≥calcitonin (salmon)	adrenomedullin (ADM, P35318) >adrenomedullin 2/intermedin (ADM2, Q7Z4H4) >*α* ‐CGRP (CALCA, P06881), amylin (IAPP, P10997) >calcitonin (salmon)	adrenomedullin (ADM, P35318) ≥adrenomedullin 2/intermedin (ADM2, Q7Z4H4) ≥*α* ‐CGRP (CALCA, P06881) >amylin (IAPP, P10997) >calcitonin (salmon)
Endogenous agonists	–	*β* ‐CGRP (CALCB, P10092) [21, 1313], *α* ‐CGRP (CALCA, P06881) [21, 1313]	adrenomedullin (ADM, P35318) [21, 1313]	adrenomedullin (ADM, P35318) [21, 568]
Antagonists	–	olcegepant (p*K*_i_ 10.7–11) [462, 753, 754, 929, 1256], telcagepant (p*K*_i_ 9.1) [1706]	–	–
Sub/family‐selective antagonists	–	–	AM‐(22‐52) (human) (p*K*_i_ 7–7.8) [754]	–
Labelled ligands	–	[^125^I]*α* CGRP (human) (Agonist), [^125^I]*α* CGRP (mouse, rat) (Agonist)	[^125^ I]AM (rat) (Agonist)	[^125^ I]AM (rat) (Agonist)

### Comments

It is important to note that a complication with the interpretation of pharmacological studies with AMY receptors in transfected cells is that most of this work has likely used a mixed population of receptors, encompassing RAMP‐coupled CTR as well as CTR alone. This means that although in binding assays human calcitonin(CALCA, P01258) has low affinity for ^125^I‐AMY binding sites, cells transfected with CTR and RAMPs can display potent CT functional responses. Transfection of human CTR with any RAMP can generate receptors with a high affinity for both salmon CT and AMY and varying affinity for different antagonists [353, 752, 753]. The major human CTR splice variant (hCT_(a)_, which does not contain an insert) with RAMP1 (i.e. the AMY_1(a)_ receptor) has a high affinity for CGRP [2057], unlike hCT_(a)_‐RAMP3 (i.e. AMY_3(a)_ receptor) [353, 752]. Actions of CGRP at AMY (and the AM2) receptors led to proposals for a CGRP2 receptor in early literature [755]. However, the AMY receptor phenotype is RAMP‐type, splice variant and cell‐line‐dependent [1376, 1614, 1964].

The ligands described have limited selectivity. Adrenomedullin has appreciable affinity for CGRP receptors. CGRP can show significant cross‐reactivity at AMY receptors and AM_2_ receptors. Adrenomedullin 2/intermedin also has high affinity for the AM_2_ receptor [818]. CGRP‐(8‐37) acts as an antagonist of CGRP (pK_i_˜ 8) and inhibits some AM and AMY responses (pK_i_˜ 6‐7). It is weak at CT receptors. Human AM‐(22‐52) has some selectivity towards AM receptors, but with modest potency (pK_i_˜ 7), limiting its use [754]. Olcegepant shows the greatest selectivity between receptors but still has significant affinity for AMY_1_ receptors [2057].

G_s_ is a prominent route for effector coupling for CLR and CTR but other pathways (e.g. Ca^2+^, ERK, Akt), and G proteins can be activated [2056]. There is evidence that CGRP‐RCP (a 148 amino‐acid hydrophilic protein, ASL(P04424) is important for the coupling of CLR to adenylyl cyclase [524].

[^125^I]‐Salmon CT is the most common radioligand for CT receptors but it has high affinity for AMY receptors and is also poorly reversible. [^125^I]‐Tyr^0^‐CGRP is widely used as a radioligand for CGRP receptors.

### Further reading on Calcitonin receptors

Booe JM et al. (2015) Structural Basis for Receptor Activity‐Modifying Protein‐Dependent Selective Peptide Recognition by a G Protein‐Coupled Receptor. Mol. Cell 58: 1040‐52 [PMID:25982113]

Hay DL et al. (2015) Amylin: Pharmacology, Physiology, and Clinical Potential. Pharmacol. Rev. 67: 564‐600 [PMID:26071095]

Hay DL et al. (2016) Receptor Activity‐Modifying Proteins (RAMPs): New Insights and Roles. Annu. Rev. Pharmacol. Toxicol. 56: 469‐87 [PMID:26514202]

Kato J et al. (2015) Bench‐to‐bedside pharmacology of adrenomedullin. Eur. J. Pharmacol. 764: 140‐8 [PMID:26144371]

Russell FA et al. (2014) Calcitonin gene‐related peptide: physiology and pathophysiology. Physiol. Rev. 94: 1099‐142 [PMID:25287861]

Russo AF. (2015) Calcitonin gene‐related peptide (CGRP): a new target for migraine. Annu. Rev. Pharmacol. Toxicol. 55: 533‐52 [PMID:25340934]

## Calcium‐sensing receptor

### Overview

The calcium‐sensing receptor (CaS, provisional nomenclature as recommended by NC‐IUPHAR[557]) responds to multiple endogenous ligands, including extracellular calcium and other divalent/trivalent cations, polyamines and polycationic peptides, L‐amino acids (particularly L‐Trp and L‐Phe), glutathione and various peptide analogues, ionic strength and extracellular pH (reviewed in [1122]). While divalent/trivalent cations, polyamines and polycations are CaS receptor agonists [234, 1618], L‐amino acids, glutamyl peptides, ionic strength and pH are allosteric modulators of agonist function [375, 557, 803, 1616, 1617]. Indeed, L‐amino acids have been identified as "co‐agonists", with both concomitant calcium and L‐amino acid binding required for full receptor activation [623, 2205]. The sensitivity of the CaS receptor to primary agonists is increased by elevated extracellular pH [270] or decreased extracellular ionic strength [1617]. This receptor bears no sequence or structural relation to the plant calcium receptor, also called CaS.

**Table:** 

Nomenclature	CaS receptor
HGNC, UniProt	CASR, P41180
Amino‐acid rank order of potency	L‐phenylalanine, L‐tryptophan, L‐histidine>L‐alanine>L‐serine, L‐proline, L‐glutamic acid>L‐aspartic acid (not L‐lysine, L‐arginine, L‐leucine and L‐isoleucine) [375]
Cation rank order of potency	Gd ^3+^>Ca ^2+^>Mg ^2+^ [234]
Glutamyl peptide rank order of potency	S‐methylglutathione ≈*γ*Glu‐Val‐Gly > glutathione >*γ*Glu‐Cys [226, 1498, 2068]
Polyamine rank order of potency	spermine>spermidine>putrescine [1618]
Allosteric modulators	ATF 936 (Negative) (pIC_50_ 8.9) [2109], encaleret (Negative) (pIC_50_ 7.9) [1795], SB‐423562 (Negative) (pIC_50_ 7.1) [1074], ronacaleret (Negative) (pIC_50_ 6.5–6.8) [92], NPS 2143 (Negative) (p*K*_B_ 6.2–6.7) [418, 1120, 1123], cinacalcet (Positive) (p*K*_B_ 5.9–6.6) [378, 418, 1120, 1123], tecalcet (Positive) (p*K*_B_ 6.2–6.6) [378, 418], AC265347 (Positive) (p*K*_B_ 6.3–6.4) [378, 1120], calhex 231 (Negative) (pIC_50_ 6.4) [1569], calindol (Positive) (p*K*_B_ 6.3) [378]

### Comments

The CaS receptor has a number of physiological functions, but it is best known for its central role in parathyroid and renal regulation of extracellular calcium homeostasis [728]. This is seen most clearly in patients with loss‐of‐function CaS receptor mutations who develop familial hypocalciuric hypercalcaemia (heterozygous mutations) or neonatal severe hyperparathyroidism (heterozygous, compound heterozygous or homozygous mutations) [728] and in Casr null mice [307, 803], which exhibit similar increases in PTH secretion and blood calcium levels. Gain‐of‐function CaS mutations are associated with autosomal dominant hypocalcaemia and Bartter syndrome type V [728].

The CaS receptor primarily couples to G_q/11_, G_12/13_ and G_i/o_[418, 634, 836, 1954], but in some cell types can couple to G_s_[1258]. However, the CaS receptor can form heteromers with Class C GABAB [308, 327] and mGlu1/5 receptors [595], which may introduce further complexity in its signalling capabilities.

Multiple other small molecule chemotypes are positive and negative allosteric modulators of the CaS receptor [980, 1441]. Further, etelcalcetide is a novel peptide agonist of the receptor [2059]. Agonists and positive allosteric modulators of the CaS receptor are termed Type I and II calcimimetics, respectively, and can suppress parathyroid hormone (PTH (PTH, P01270)) secretion [1443]. Negative allosteric modulators are called calcilytics and can act to increase PTH (PTH, P01270) secretion [1442].

Where functional pK_B_ values are provided for allosteric modulators, this refers to ligand affinity determined in an assay that measures a functional readout of receptor activity (i.e. a receptor signalling assay), as opposed to affinity determined in a radioligand binding assay. The functional pK_B_ may differ depending on the signalling pathway studied. Consult the 'More detailed page' for the assay description, as well as other functional readouts.

### Further reading on Calcium‐sensing receptor

Breitwieser GE. (2012) Minireview: the intimate link between calcium sensing receptor trafficking and signaling: implications for disorders of calcium homeostasis. Mol. Endocrinol. 26: 1482‐95 [PMID:22745192]

Brown EM. (2013) Role of the calcium‐sensing receptor in extracellular calcium homeostasis. Best Pract. Res. Clin. Endocrinol. Metab. 27: 333‐43 [PMID:23856263]

Conigrave AD et al. (2013) Calcium‐sensing receptor (CaSR): pharmacological properties and signaling pathways. Best Pract. Res. Clin. Endocrinol. Metab. 27: 315‐31 [PMID:23856262]

Nemeth EF et al. (2013) Calcimimetic and calcilytic drugs for treating bone and mineral‐related disorders. Best Pract. Res. Clin. Endocrinol. Metab. 27: 373‐84 [PMID:23856266]

## Cannabinoid receptors

### Overview

Cannabinoid receptors (nomenclature as agreed by the NC‐IUPHAR Subcommittee on Cannabinoid Receptors [1564]) are activated by endogenous ligands that include N‐arachidonoylethanolamine (anandamide), N‐homo‐ *γ*‐linolenoylethanolamine, N‐docosatetra‐7,10,13,16‐enoylethanolamine and 2‐arachidonoylglycerol. Potency determinations of endogenous agonists at these receptors are complicated by the possibility of differential susceptibility of endogenous ligands to enzymatic conversion [35].

There are currently three licenced cannabinoid medicines each of which contains a compound that can activate CB_1_ and CB_2_ receptors [1562]. Two of these medicines were developed to suppress nausea and vomiting produced by chemotherapy. These are nabilone (Cesamet®), a synthetic CB_1_/CB_2_ receptor agonist, and synthetic Δ^9^ ‐tetrahydrocannabinol(Marinol®; dronabinol), which can also be used as an appetite stimulant. The third medicine, Sativex®, contains mainly Δ^9^ ‐tetrahydrocannabinol and cannabidiol, both extracted from cannabis, and is used to treat multiple sclerosis and cancer pain.

**Table:** 

Nomenclature	CB_1_ receptor	CB_2_ receptor
HGNC, UniProt	CNR1, P21554	CNR2, P34972
Agonists	cannabinol (Partial agonist) [535, 1801]	–
Sub/family‐selective agonists	HU‐210 [535, 1801], CP55940 [535, 1676, 1801], WIN55212‐2 [535, 1798, 1801], Δ^9^ ‐tetrahydrocannabinol (Partial agonist) [535, 1801]	HU‐210 [535, 1653, 1801], WIN55212‐2 [535, 1798, 1801], CP55940 [535, 1676, 1801], Δ^9^ ‐tetrahydrocannabinol (Partial agonist) [113, 535, 1653, 1801]
Selective agonists	arachidonyl‐2‐chloroethylamide [791] – Rat, arachidonylcyclopropylamide [791] – Rat, O‐1812 [443] – Rat, R‐(+)‐methanandamide [976] – Rat	JWH‐133 [844, 1563], L‐759,633 [607, 1676], AM1241 [2175], L‐759,656 [607, 1676], HU‐308 [734]
Selective antagonists	rimonabant (p*K*_i_ 7.9–8.7) [534, 535, 1660, 1687, 1801], AM251 (p*K*_i_ 8.1) [1094] – Rat, AM281 (p*K*_i_ 7.9) [1093] – Rat, LY320135 (p*K*_i_ 6.9) [534]	SR144528 (p*K*_i_ 8.3–9.2) [1661, 1676], AM‐630 (p*K*_i_ 7.5) [1676]
Allosteric modulators	GAT100 (Negative) (pEC_50_ 7.7) [1070], ZCZ011 (Positive) (pEC_50_ 6.3) [857] – Mouse, cannabidiol (Negative) [1100]	–
Labelled ligands	[^3^ H]rimonabant (Antagonist) (p*K*_d_ 8.9–10) [211, 799, 932, 1568, 1662, 1811, 1948] – Rat	–

### Comments

Both CB_1_ and CB_2_ receptors may be labelled with [^3^ H]CP55940(0.5 nM; [1801]) and [^3^ H]WIN55212‐2(2–2.4 nM; [1826, 1852]). Anandamide is also an agonist at vanilloid receptors (TRPV1) and PPARs[1484]. There is evidence for an allosteric site on the CB_1_ receptor [1603]. All of the compounds listed as antagonists behave as inverse agonists in some bioassay systems [1564]. For some cannabinoid receptor ligands, additional pharmacological targets that include GPR55 and GPR119 have been identified [1564]. Moreover, GPR18, GPR55 and GPR119, although showing little structural similarity to CB_1_ and CB_2_receptors, respond to endogenous agents that are structurally similar to the endogenous cannabinoid ligands [1564].

### Further reading on Cannabinoid receptors

Howlett AC et al. (2002) International Union of Pharmacology. XXVII. Classification of cannabinoid receptors. Pharmacol. Rev. 54: 161‐202 [PMID:12037135]

Pertwee RG. (2010) Receptors and channels targeted by synthetic cannabinoid receptor agonists and antagonists. Curr. Med. Chem. 17: 1360‐81 [PMID:20166927]

Pertwee RG et al. (2010) International Union of Basic and Clinical Pharmacology. LXXIX. Cannabinoid receptors and their ligands: beyond CB1 and CB2. Pharmacol. Rev. 62: 588‐631 [PMID:21079038]

## Chemerin receptor

### Overview

The chemerin receptor (nomenclature as recommended by NC‐IUPHAR[414]) is activated by the lipid‐derived, anti‐inflammatory ligand resolvin E1 (RvE1), which is the result of sequential metabolism of EPA by aspirin‐modified cyclooxygenase and lipoxygenase [60, 61]. In addition, two GPCRs for resolvin D1 (RvD1) have been identified, FPR2/ALX, the lipoxin A_4_ receptor, and GPR32, an orphan receptor [1052].

**Table:** 

Nomenclature	chemerin receptor
HGNC, UniProt	CMKLR1, Q99788
Potency order of endogenous ligands	resolvin E1>chemerin C‐terminal peptide>18R‐HEPE>EPA [60]
Selective agonists	resolvin E1
Labelled ligands	[^3^ H]resolvin E1 (Agonist) [60, 61]

### Comments

CCX832 (structure not disclosed) is a selective antagonist, pK_i_=9.2 [969].

## Chemokine receptors

### Overview

Chemokine receptors (nomenclature as agreed by the NC‐IUPHAR Subcommittee on Chemokine Receptors [81, 1402, 1403]) comprise a large subfamily of 7TM proteins that bind one or more chemokines, a large family of small cytokines typically possessing chemotactic activity for leukocytes. Chemokine receptors can be divided by function into two main groups: G protein‐coupled chemokine receptors, which mediate leukocyte trafficking, and "Atypical chemokine receptors", which may signal through non‐G protein‐coupled mechanisms and act as chemokine scavengers to downregulate inflammation or shape chemokine gradients [81].

Chemokines in turn can be divided by structure into four subclasses by the number and arrangement of conserved cysteines. CC (also known as *β*‐chemokines; n= 28), CXC (also known as *α*‐chemokines; n= 17) and CX3C (n= 1) chemokines all have four conserved cysteines, with zero, one and three amino acids separating the first two cysteines respectively. C chemokines (n= 2) have only the second and fourth cysteines found in other chemokines. Chemokines can also be classified by function into homeostatic and inflammatory subgroups. Most chemokine receptors are able to bind multiple high‐affinity chemokine ligands, but the ligands for a given receptor are almost always restricted to the same structural subclass. Most chemokines bind to more than one receptor subtype. Receptors for inflammatory chemokines are typically highly promiscuous with regard to ligand specificity, and may lack a selective endogenous ligand. G protein‐coupled chemokine receptors are named acccording to the class of chemokines bound, whereas ACKR is the root acronym for atypical chemokine receptors [82]. Listed are those human agonists with EC_50_ values <50nM in either Ca^2+^ flux or chemotaxis assays at human recombinant G protein‐coupled chemokine receptors expressed in mammalian cell lines. There can be substantial cross‐species differences in the sequences of both chemokines and chemokine receptors, and in the pharmacology and biology of chemokine receptors. Endogenous and microbial non‐chemokine ligands have also been identified for chemokine receptors. Many chemokine receptors function as HIV co‐receptors, but CCR5 is the only one demonstrated to play an essential role in HIV/AIDS pathogenesis. The tables include both standard chemokine receptor names [2191] and aliases. Numerical data quoted are typically pK_i_ or pIC_50_ values from radioligand binding to heterologously expressed receptors.

**Table:** 

Nomenclature	CCR1	CCR2	CCR3
HGNC, UniProt	CCR1, P32246	CCR2, P41597	CCR3, P51677
Endogenous agonists	CCL3 (CCL3, P10147) [342, 370, 783, 2228], CCL23 (CCL23, P55773) [342], CCL5 (CCL5, P13501) [370, 783], CCL7 (CCL7, P80098) [342, 703], CCL15 (CCL15, Q16663) [387], CCL14 (CCL14, Q16627) [342], CCL13 (CCL13, Q99616), CCL8 (CCL8, P80075)	CCL2 (CCL2, P13500) [387, 1224, 1347, 1533, 1996], CCL13 (CCL13, Q99616) [1224, 1996], CCL7 (CCL7, P80098) [387, 1224, 1996], CCL11 (CCL11, P51671) (Partial agonist) [1224, 1533], CCL16 (CCL16, O15467)	CCL13 (CCL13, Q99616) [1385, 1996], CCL24 (CCL24, O00175) [1385, 1533], CCL5 (CCL5, P13501) [409], CCL7 (CCL7, P80098) [409], CCL11 (CCL11, P51671) [480, 1009, 1385, 1700, 1996], CCL26 (CCL26, Q9Y258) [1009, 1385, 1533], CCL15 (CCL15, Q16663) [387], CCL28 (CCL28, Q9NRJ3), CCL8 (CCL8, P80075)
Agonists	–	–	CCL11 {Mouse} [409]
Endogenous antagonists	CCL4 (CCL4, P13236) (p*K*_i_ 7.1–7.8) [342, 370]	CCL26 (CCL26, Q9Y258) (pIC_50_ 8.5) [1533]	CXCL10 (CXCL10, P02778), CXCL11 (CXCL11, O14625), CXCL9 (CXCL9, Q07325)
Selective antagonists	BX 471 (p*K*_i_ 8.2–9) [1164], compound 2b‐1 (pIC_50_ 8.7) [1429], UCB35625 (pIC_50_ 8) [1700], CP‐481,715 (p*K*_d_ 8) [646]	GSK Compound 34 (p*K*_i_ 7.6)	banyu (I) (Inverse agonist) (p*K*_i_ 8.5) [2063], SB328437 (p*K*_i_ 8.4), BMS compound 87b (p*K*_i_ 8.1) [2048]
Labelled ligands	[^125^ I]CCL7 (human) (Agonist) [131], [^125^ I]CCL3 (human) (Agonist) [131, 656, 1719], [^125^ I]CCL5 (human) (Agonist) [1719]	[^125^ I]CCL2 (human) (Agonist), [^125^ I]CCL7 (human) (Agonist)	[^125^ I]CCL11 (human) (Antagonist) (p*K*_d_ 8.3) [2063], [^125^ I]CCL5 (human) (Agonist), [^125^ I]CCL7 (human) (Agonist)

**Table:** 

Nomenclature	CCR4	CCR5	CCR6	CCR7	CCR8	CCR9	CCR10
HGNC, UniProt	CCR4, P51679	CCR5, P51681	CCR6, P51684	CCR7, P32248	CCR8, P51685	CCR9, P51686	CCR10, P46092
Endogenous agonists	CCL22 (CCL22, O00626) [862], CCL17 (CCL17, Q92583) [862]	CCL5 (CCL5, P13501) [78, 1424, 1685], CCL4 (CCL4, P13236) [1424, 1685], CCL8 (CCL8, P80075) [1685], CCL3 (CCL3, P10147) [1424, 1685, 2228], CCL11 (CCL11, P51671) [161], CCL2 (CCL2, P13500) [1424], CCL14 (CCL14, Q16627) [1424], CCL16 (CCL16, O15467)	CCL20 (CCL20, P78556) [20, 77, 1598], beta‐defensin 4A (DEFB4A DEFB4B, O15263) [2169]	CCL21 (CCL21, O00585) [2189], CCL19 (CCL19, Q99731) [1517, 2188, 2189]	CCL1 (CCL1, P22362) [403, 745, 863], CCL8 {Mouse} – Mouse	CCL25 (CCL25, O15444)	CCL27 (CCL27, Q9Y4X3) [816], CCL28 (CCL28, Q9NRJ3)
Agonists	vMIP‐III	R5‐HIV‐1 gp120	–	–	vMIP‐I [403, 863]	–	–
Endogenous antagonists	–	CCL7 (CCL7, P80098) (p*K*_i_ 7.5) [1424]	–	–	–	–	–
Antagonists	–	vicriviroc (p*K*_i_ 9.1) [1879], ancriviroc (p*K*_i_ 7.8–8.7) [1237, 1523, 1879]	–	–	–	–	–
Selective antagonists	compound 8ic (pIC_50_ 7.7) [2186], plerixafor (pIC_50_ 6.2) [577]	E913 (pIC_50_ 8.7) [1238], aplaviroc (p*K*_i_ 8.5) [1237], maraviroc (pIC_50_ 8.1) [1424], TAK‐779 (p*K*_i_ 7.5) [1237], MRK‐1 [1073] – Rat	–	–	vMCC‐I (pIC_50_ 9.4) [403]	–	–
Selective allosteric modulators	–	–	–	–	–	vercirnon (Antagonist) (pIC_50_ 8.2) [2060]	–
Antibodies	mogamulizumab (Inhibition) [54, 1799]	–	–	–	–	–	–
Labelled ligands	[^125^ I]CCL17 (human) (Agonist), [^125^ I]CCL27 (human) (Agonist)	[^125^ I]CCL4 (human) (Agonist) [1424], [^125^ I]CCL3 (human) (Agonist), [^125^ I]CCL5 (human) (Agonist), [^125^ I]CCL8 (human) (Agonist)	[^125^ I]CCL20 (human) (Agonist) [675]	[^125^ I]CCL19 (human) (Agonist), [^125^ I]CCL21 (human) (Agonist) [899]	[^125^ I]CCL1 (human) (Agonist) [863, 1671]	[^125^ I]CCL25 (human) (Agonist)	–

**Table:** 

Nomenclature	CXCR1	CXCR2	CXCR3	CXCR4	CXCR5	CXCR6	CX_3_CR1
HGNC, UniProt	CXCR1, P25024	CXCR2, P25025	CXCR3, P49682	CXCR4, P61073	CXCR5, P32302	CXCR6, O00574	CX3CR1, P49238
Endogenous agonists	CXCL8 (CXCL8, P10145) [145, 711, 1133, 2121, 2137], CXCL6 (CXCL6, P80162) [2141]	CXCL1 (CXCL1, P09341) [711, 1133, 2137], CXCL8 (CXCL8, P10145) [145, 711, 1133, 2121, 2137], CXCL7 (PPBP, P02775) [18], CXCL3 (CXCL3, P19876) [18], CXCL2 (CXCL2, P19875) [18], CXCL5 (CXCL5, P42830) [18], CXCL6 (CXCL6, P80162) [2141]	CXCL11 (CXCL11, O14625) [768], CXCL10 (CXCL10, P02778) [768, 2093], CXCL9 (CXCL9, Q07325) [768, 2093]	CXCL12 *α* (CXCL12, P48061) [782, 1202], CXCL12 *β* (CXCL12, P48061) [782]	CXCL13 (CXCL13, O43927) [103]	CXCL16 (CXCL16, Q9H2A7) [2116]	CX_3_CL1 (CX3CL1, P78423) [608]
Agonists	vCXCL1 [1223], HIV‐1 matrix protein p17 [637]	vCXCL1 [1223], HIV‐1 matrix protein p17 [637]	–	–	–	–	–
Selective agonists	–	–	–	ALX40‐4C (Partial agonist) [2213], X4‐HIV‐1 gp120	–	–	–
Endogenous antagonists	–	–	CCL11 (CCL11, P51671) (p*K*_i_ 7.2) [2093], CCL7 (CCL7, P80098) (p*K*_i_ 6.6) [2093]	–	–	–	–
Antagonists	–	–	–	plerixafor (p*K*_i_ 7) [2213]	–	–	–
Selective antagonists	–	navarixin (pIC_50_ 10.3) [81, 484], danirixin (pIC_50_ 7.9) [1343], SB 225002 (pIC_50_ 7.7) [2103], elubirixin (pIC_50_ 7.7) [81], SX‐517 (pIC_50_ 7.2) [1236]	–	T134 (pIC_50_ 8.4) [1929], X4P‐001 (pIC_50_ 7.9) [1819], HIV‐Tat	–	–	–
Allosteric modulators	reparixin (Negative) (pIC_50_ 9) [145]	reparixin (Negative) (pIC_50_ 6.4) [145]	–	–	–	–	–
Labelled ligands	[^125^ I]CXCL8 (human) (Agonist) [711, 1658]	[^125^ I]CXCL8 (human) (Agonist) [711, 1658], [^125^ I]CXCL1 (human) (Agonist), [^125^ I]CXCL5 (human) (Agonist), [^125^ I]CXCL7 (human) (Agonist)	[^125^ I]CXCL10 (human) (Agonist), [^125^ I]CXCL11 (human) (Agonist)	[^125^ I]CXCL12 *α* (human) (Agonist) [444, 782]	[^125^ I]CXCL13 (mouse) (Agonist) [227] – Mouse	[^125^ I]CXCL16 (human) (Agonist)	[^125^ I]CX_3_CL1(human) (Agonist)

**Table:** 

Nomenclature	XCR1	ACKR1	ACKR2	ACKR3	ACKR4	CCRL2
HGNC, UniProt	XCR1, P46094	ACKR1, Q16570	ACKR2, O00590	ACKR3, P25106	ACKR4, Q9NPB9	CCRL2, O00421
Endogenous ligands	–	CXCL5 (CXCL5, P42830), CXCL6 (CXCL6, P80162), CXCL8 (CXCL8, P10145), CXCL11 (CXCL11, O14625), CCL2 (CCL2, P13500), CCL5 (CCL5, P13501), CCL7 (CCL7, P80098), CCL11 (CCL11, P51671), CCL14 (CCL14, Q16627), CCL17 (CCL17, Q92583)	–	–	–	chemerin C‐terminal peptide, CCL19 (CCL19, Q99731) [101]
Endogenous agonists	XCL1 (XCL1, P47992) [564], XCL2 (XCL2, Q9UBD3) [564]	–	CCL2 (CCL2, P13500), CCL3 (CCL3, P10147), CCL4 (CCL4, P13236), CCL5 (CCL5, P13501), CCL7 (CCL7, P80098), CCL8 (CCL8, P80075), CCL11 (CCL11, P51671), CCL13 (CCL13, Q99616), CCL14 (CCL14, Q16627), CCL17 (CCL17, Q92583), CCL22 (CCL22, O00626)	CXCL12 *α* (CXCL12, P48061) [674, 1854], CXCL11 (CXCL11, O14625)	CCL19 (CCL19, Q99731) [2085], CCL25 (CCL25, O15444) [2085], CCL21 (CCL21, O00585) [2085]	–
Comments	XCL1 cannot be iodinated, but a secreted alkaline phophatase (SEAP)‐XCL1 fusion peptide can be used as a probe at XCR1.	ACKR1 is used by Plasmodium vivax and Plasmodium knowlsei for entering erythrocytes.	–	Several lines of evidence have suggested that adrenomedullin is a ligand for ACKR3; however, classical direct binding to the receptor has not yet been convincingly demonstrated.	–	–

### Comments

Specific chemokine receptors facilitate cell entry by microbes, such as ACKR1 for Plasmodium vivax, and CCR5 and CXCR4 for HIV‐1. Virally encoded chemokine receptors are known (e.g. US28, a homologue of CCR1 from human cytomegalovirus and ORF74, which encodes a homolog of CXCR2 in Herpesvirus saimiri and gamma‐Herpesvirus‐68), but their role in viral life cycles is not established. Viruses can exploit or subvert the chemokine system by producing chemokine antagonists and scavengers. Two chemokine receptor antagonists have now been approved by the FDA: the CCR5 antagonist maraviroc (Pfizer) for treatment of HIV/AIDS in patients with CCR5‐using strains; and the CXCR4 antagonist plerixafor (Sanofi) for hematopoietic stem cell mobilization with G‐CSF(CSF3, P09919) in patients undergoing transplantation in the context of chemotherapy for Hodgkins' Disease and multiple myeloma.

### Further reading on Chemokine receptors

Bachelerie F et al. (2015) An atypical addition to the chemokine receptor nomenclature: IUPHAR Review "15". Br. J. Pharmacol. [PMID:25958743]

Koelink PJ et al. (2012) Targeting chemokine receptors in chronic inflammatory diseases: an extensive review. Pharmacol. Ther. 133: 1‐18 [PMID:21839114]

Murphy PM. (2002) International Union of Pharmacology. XXX. Update on chemokine receptor nomenclature. Pharmacol. Rev. 54: 227‐9 [PMID:12037138]

Murphy PM et al. (2000) International Union of Pharmacology. XXII. Nomenclature for chemokine receptors. Pharmacol. Rev. 52: 145‐176 [PMID:10699158]

Scholten DJ et al. (2012) Pharmacological modulation of chemokine receptor function. Br. J. Pharmacol. 165: 1617‐43 [PMID:21699506]

## Cholecystokinin receptors

### Overview

Cholecystokinin receptors (nomenclature as agreed by the NC‐IUPHAR Subcommittee on CCK receptors [1471]) are activated by the endogenous peptides cholecystokinin‐8 (CCK‐8(CCK, P06307)), CCK‐33(CCK, P06307), CCK‐58(CCK, P06307) and gastrin (gastrin‐17(GAST, P01350)). There are only two distinct subtypes of CCK receptors, CCK_1_ and CCK_2_ receptors [1038, 2073], with some alternatively spliced forms most often identified in neoplastic cells. The CCK receptor subtypes are distinguished by their peptide selectivity, with the CCK_1_ receptor requiring the carboxyl‐terminal heptapeptide‐amide that includes a sulfated tyrosine for high affinity and potency, while the CCK_2_ receptor requires only the carboxyl‐terminal tetrapeptide shared by each CCK and gastrin peptides. These receptors have characteristic and distinct distributions, with both present in both the central nervous system and peripheral tissues.

**Table:** 

Nomenclature	CCK_1_ receptor	CCK_2_ receptor
HGNC, UniProt	CCKAR, P32238	CCKBR, P32239
Potency order of endogenous ligands	CCK‐8 (CCK, P06307) ≫gastrin‐17 (GAST, P01350), desulfated cholecystokinin‐8>CCK‐4 (CCK, P06307)	CCK‐8 (CCK, P06307) ≥gastrin‐17 (GAST, P01350), desulfated cholecystokinin‐8, CCK‐4 (CCK, P06307)
Endogenous agonists	–	desulfated cholecystokinin‐8 [1135], gastrin‐17 (GAST, P01350) [845] – Mouse, CCK‐4 (CCK, P06307) [871], desulfated gastrin‐14 (GAST, P01350), desulfated gastrin‐17 (GAST, P01350), desulfated gastrin‐34 (GAST, P01350), desulfated gastrin‐71 (GAST, P01350), gastrin‐14 (GAST, P01350), gastrin‐34 (GAST, P01350), gastrin‐71 (GAST, P01350)
Selective agonists	A‐71623 [67] – Rat, JMV180 [971], GW‐5823 [772]	RB‐400 [129] – Rat, PBC‐264 [886] – Rat
Antagonists	lintitript (pIC_50_ 8.3) [667]	–
Selective antagonists	devazepide (pIC_50_ 9.7) [845] – Rat, T‐0632 (pIC_50_ 9.6) [1935] – Rat, PD‐140548 (pIC_50_ 8.6) [1817] – Rat, lorglumide (pIC_50_ 6.7–8.2) [845, 875] – Rat	YF‐476 (pIC_50_ 9.7) [201, 1927], GV150013 (pIC_50_ 9.4) [2006], L‐740093 (pIC_50_ 9.2) [1464], YM‐022 (pIC_50_ 9.2) [1464], JNJ‐26070109 (pIC_50_ 8.5) [1390], L‐365260 (pIC_50_ 8.4) [1135], RP73870 (pIC_50_ 8) [1181] – Rat, LY262691 (pIC_50_ 7.5) [1632] – Rat
Labelled ligands	[^3^ H]devazepide (Antagonist) (p*K*_d_ 9.7) [306], [^125^ I]DTyr‐Gly‐[(Nle28,31)CCK‐26‐33 (Agonist) [1599]	[^3^ H]PD140376 (Antagonist) (p*K*_i_ 9.7–10) [849] – Guinea pig, [^125^ I]PD142308 (Antagonist) (p*K*_d_ 9.6) [820] – Guinea pig, [^125^ I]DTyr‐Gly‐[(Nle28,31)CCK‐26‐33 (Agonist) [1599], [^125^ I]gastrin (Agonist), [^3^ H]gastrin (Agonist), [^3^ H]L365260 (Antagonist) (p*K*_d_ 8.2–8.5) [1464], [^125^ I]‐BDZ_2_ (Antagonist) (p*K*_i_ 8.4) [25]

### Comments

While a cancer‐specific CCK receptor has been postulated to exist, which also might be responsive to incompletely processed forms of CCK (Gly‐extended forms), this has never been isolated. An alternatively spliced form of the CCK_2_ receptor in which intron 4 is retained, adding 69 amino acids to the intracellular loop 3 (ICL3) region, has been described to be present particularly in certain neoplasms where mRNA mis‐splicing has been commonly observed [1833], but it is not clear that this receptor splice form plays a special role in carcinogenesis. Another alternative splicing event for the CCK_2_ receptor was reported [1850], with alternative donor sites in exon 4 resulting in long (452 amino acids) and short (447 amino acids) forms of the receptor differing by five residues in ICL3, however, no clear functional differences have been observed.

### Further reading on Cholecystokinin receptors

Cawston EE et al. (2010) Therapeutic potential for novel drugs targeting the type 1 cholecystokinin receptor. Br. J. Pharmacol. 159: 1009‐21 [PMID:19922535]

Dockray GJ. (2009) Cholecystokinin and gut‐brain signalling. Regul. Pept. 155: 6‐10 [PMID:19345244]

Dufresne M et al. (2006) Cholecystokinin and gastrin receptors. Physiol. Rev. 86: 805‐47 [PMID:16816139]

Miller LJ et al. (2008) Structural basis of cholecystokinin receptor binding and regulation. Pharmacol. Ther. 119: 83‐95 [PMID:18558433]

## Class Frizzled GPCRs

### Overview

Receptors of the Class Frizzled (FZD, nomenclature as agreed by the NC‐IUPHAR subcommittee on the Class Frizzled GPCRs [1747]), are GPCRs originally identified in Drosophila [300], which are highly conserved across species. While SMO shows structural resemblance to the 10 FZDs, it is functionally separated as it mediates effects in the Hedgehog signaling pathway [1747]. FZDs are activated by WNTs, which are cysteine‐rich lipoglycoproteins with fundamental functions in ontogeny and tissue homeostasis. FZD signalling was initially divided into two pathways, being either dependent on the accumulation of the transcription regulator *β* ‐catenin(CTNNB1, P35222) or being *β*‐catenin‐independent (often referred to as canonical vs. non‐canonical WNT/FZD signalling, respectively). WNT stimulation of FZDs can, in cooperation with the low density lipoprotein receptors LRP5 (O75197) and LRP6(O75581), lead to the inhibition of a constitutively active destruction complex, which results in the accumulation of *β*‐catenin and subsequently its translocation to the nucleus. *β*‐Catenin, in turn, modifies gene transcription by interacting with TCF/LEF transcription factors. *β*‐Catenin‐independent FZD signalling is far more complex with regard to the diversity of the activated pathways. WNT/FZD signalling can lead to the activation of heterotrimeric G proteins [447], the elevation of intracellular calcium [1828], activation of cGMP‐specific PDE6 [19] and elevation of cAMP as well as RAC‐1, JNK, Rho and Rho kinase signalling [730]. Furthermore, the phosphoprotein Dishevelled constitutes a key player in WNT/FZD signalling. As with other GPCRs, members of the Frizzled family are functionally dependent on the arrestin scaffolding protein for internalization [321], as well as for *β*‐catenin‐dependent [242] and ‐independent [243, 986] signalling. The pattern of cell signalling is complicated by the presence of additional ligands, which can enhance or inhibit FZD signalling (secreted Frizzled‐related proteins (sFRP), Wnt‐inhibitory factor(WIF1, Q9Y5W5) (WIF), sclerostin(SOST, Q9BQB4) or Dickkopf (DKK)), as well as modulatory (co)‐receptors with Ryk, ROR1, ROR2 and Kremen, which may also function as independent signalling proteins.

**Table:** 

Nomenclature	FZD_1_	FZD_2_	FZD_3_	FZD_4_	FZD_5_	FZD_6_	FZD_7_
HGNC, UniProt	FZD1, Q9UP38	FZD2, Q14332	FZD3, Q9NPG1	FZD4, Q9ULV1	FZD5, Q13467	FZD6, O60353	FZD7, O75084

**Table:** 

Nomenclature	FZD_8_	FZD_9_	FZD_10_	SMO
HGNC, UniProt	FZD8, Q9H461	FZD9, O00144	FZD10, Q9ULW2	SMO, Q99835
Antagonists	–	–	–	saridegib (pIC_50_ 8.9) [1981], glasdegib (pIC_50_ 8.3) [1398], sonidegib (p*K*_i_ 8.2) [2065]
Selective antagonists	–	–	–	vismodegib (p*K*_i_ 7.8) [2065]

### Comments

There is limited knowledge about WNT/FZD specificity and which molecular entities determine the signalling outcome of a specific WNT/FZD pair. Understanding of the coupling to G proteins is incomplete (see [447]). There is also a scarcity of information on basic pharmacological characteristics of FZDs, such as binding constants, ligand specificity or concentration‐response relationships [984].

Ligands associated with FZD signalling

WNTs: Wnt‐1(WNT1, P04628), Wnt‐2(WNT2, P09544) (also known as Int‐1‐related protein), Wnt‐2b(WNT2B, Q93097) (also known as WNT‐13), Wnt‐3(WNT3, P56703) , Wnt‐3a(WNT3A, P56704), Wnt‐4(WNT4, P56705), Wnt‐5a(WNT5A, P41221) , Wnt‐5b(WNT5B, Q9H1J7), Wnt‐6(WNT6, Q9Y6F9), Wnt‐7a(WNT7A, O00755), Wnt‐7b(WNT7B, P56706), Wnt‐8a(WNT8A, Q9H1J5), Wnt‐8b(WNT8B, Q93098), Wnt‐9a(WNT9A, O14904) (also known as WNT‐14), Wnt‐9b(WNT9B, O14905) (also known as WNT‐15 or WNT‐14b), Wnt‐10a(WNT10A, Q9GZT5), Wnt‐10b(WNT10B, O00744) (also known as WNT‐12), Wnt‐11(WNT11, O96014) and Wnt‐16(WNT16, Q9UBV4).

Extracellular proteins that interact with FZDs: norrin(NDP, Q00604), R‐spondin‐1(RSPO1, Q2MKA7), R‐spondin‐2(RSPO2, Q6UXX9) , R‐spondin‐3(RSPO3, Q9BXY4), R‐spondin‐4(RSPO4, Q2I0M5), sFRP‐1(SFRP1, Q8N474), sFRP‐2(SFRP2, Q96HF1), sFRP‐3(FRZB, Q92765), sFRP‐4(SFRP4, Q6FHJ7), sFRP‐5(SFRP5, Q6FHJ7).

Extracellular proteins that interact with WNTs or LRPs: Dickkopf 1(DKK1, O94907), WIF1 (Q9Y5W5), sclerostin(SOST, Q9BQB4), kremen 1(KREMEN1, Q96MU8) and kremen 2 (KREMEN2, Q8NCW0)

Small exogenous ligands: Foxy‐5 [1910], Box‐5, UM206 [1086], and XWnt8 (P28026) also known as mini‐Wnt8.

### Further reading on Class Frizzled GPCRs

Angers S et al. (2009) Proximal events in Wnt signal transduction. Nat. Rev. Mol. Cell Biol. 10: 468‐77 [PMID:19536106]

Schulte G. (2015) Frizzleds and WNT/*β*‐catenin signaling–The black box of ligand‐receptor selectivity, complex stoichiometry and activation kinetics. Eur. J. Pharmacol. 763: 191‐5 [PMID:26003275]

van Amerongen R. (2012) Alternative Wnt pathways and receptors. Cold Spring Harb Perspect Biol 4: [PMID:22935904]

Wang Y et al. (2016) Frizzled Receptors in Development and Disease. Curr. Top. Dev. Biol. 117: 113‐39 [PMID:26969975]

## Complement peptide receptors

### Overview

Complement peptide receptors (nomenclature as agreed by the NC‐IUPHAR subcommittee on Complement peptide receptors [1015]) are activated by the endogenous ˜ 75 amino‐acid anaphylatoxin polypeptides C3a(C3, P01024) and C5a(C5, P01031), generated upon stimulation of the complement cascade.

**Table:** 

Nomenclature	C3a receptor	C5a_1_ receptor	C5a_2_ receptor
HGNC, UniProt	C3AR1, Q16581	C5AR1, P21730	C5AR2, Q9P296
Potency order of endogenous ligands	C3a (C3, P01024) >C5a (C5, P01031) [41]	C5a (C5, P01031), C5a des‐Arg (C5) >C3a (C3, P01024) [41]	–
Endogenous agonists	–	ribosomal protein S19 (RPS19, P39019) [2160]	–
Agonists	E7 [43], compound 17 [1644], compound 21 [1643], Ac‐RHYPLWR [707]	N‐methyl‐Phe‐Lys‐Pro‐D‐Cha‐Cha‐D‐Arg‐CO_2_H [959, 1035]	–
Selective agonists	–	–	P59 (Biased agonist) [396], P32 (Biased agonist) [396]
Antagonists	SB290157 (pIC_50_ 7.6) [40], compound 4 (pIC_50_ 5.9) [1643]	avacopan (pIC_50_ 9.7) [125], W54011 (p*K*_i_ 8.7) [1893], DF2593A (pIC_50_ 8.3) [1380], AcPhe‐Orn‐Pro‐D‐Cha‐Trp‐Arg (pIC_50_ 7.9) [2128], N‐methyl‐Phe‐Lys‐Pro‐D‐Cha‐Trp‐D‐Arg‐CO_2_H (pIC_50_ 7.2) [1035]	–
Labelled ligands	[^125^ I]C3a (human) (Agonist) [310]	[^125^ I]C5a (human) (Agonist) [843]	[^125^ I]C5a (human) (Agonist)

### Comments

SB290157 has also been reported to have agonist properties at the C3a receptor [1282]. The putative chemoattractant receptor termed C5a_2_ (also known as GPR77, C5L2) binds [^125^I]C5a with no clear signalling function, but has a putative role opposing inflammatory responses [267, 599, 616]. Binding to this site may be displaced with the rank order C5a des‐Arg(C5)>C5a(C5, P01031) [267, 1508] while there is controversy over the ability of C3a(C3, P01024) and C3a des Arg(C3, P01024) to compete [817, 936, 937, 1508]. C5a_2_ appears to lack G protein signalling and has been termed a decoy receptor [1753]. However, C5a_2_ does recruit arrestin after ligand binding, which might provide a signaling pathway for this receptor [94, 2015], and forms heteromers with C5a_1_. C5a, but not C5a‐des Arg, induces upregulation of heteromer formation between complement C5a receptors C5a_1_ and C5a_2_[395]. There are also reports of pro‐inflammatory activity of C5a_2_, mediated by HMGB1, but the signaling pathway that underlies this is currently unclear (reviewed in [1161]). More recently, work in T cells has shown that C5a_1_ and C5a_2_ act in opposition to each other and that altering the equilibrium between the two receptors, by differential expression or production of C5a‐des Arg (which favours C5a_2_), can affect the final cellular response [57].

### Further reading on Complement peptide receptors

Arbore G et al. (2016) A novel "complement‐metabolism‐inflammasome axis" as a key regulator of immune cell effector function. Eur. J. Immunol. 46: 1563‐73 [PMID:27184294]

Klos A et al. (2013) International Union of Pharmacology. LXXXVII. Complement peptide C5a, C4a, and C3a receptors. Pharmacol. Rev. 65: 500‐43 [PMID:23383423]

Li R et al. (2013) C5L2: a controversial receptor of complement anaphylatoxin, C5a. FASEB J. 27: 855‐64 [PMID:23239822]

Monk PN et al. (2007) Function, structure and therapeutic potential of complement C5a receptors. Br. J. Pharmacol. 152: 429‐48 [PMID:17603557]

## Corticotropin‐releasing factor receptors

### Overview

Corticotropin‐releasing factor (CRF, nomenclature as agreed by the NC‐IUPHAR subcommittee on Corticotropin‐releasing Factor Receptors [750]) receptors are activated by the endogenous peptides corticotrophin‐releasing hormone (CRH, P06850), a 41 amino‐acid peptide, urocortin 1(UCN, P55089), 40 amino‐acids, urocortin 2 (UCN2, Q96RP3), 38 amino‐acids and urocortin 3 (UCN3, Q969E3), 38 amino‐acids. CRF_1_ and CRF_2_ receptors are activated non‐selectively by corticotrophin‐releasing hormone (CRH, P06850) and urocortin 1 (UCN, P55089). Binding to CRF receptors can be conducted using [^125^I]Tyr^0^‐CRF or [^125^ I]Tyr ^0^‐sauvagine with K_d_ values of 0.1‐0.4 nM. CRF_1_ and CRF_2_ receptors are non‐selectively antagonized by *α* ‐helical CRF, D‐Phe‐CRF‐(12‐41) and astressin.

**Table:** 

Nomenclature	CRF_1_ receptor	CRF_2_ receptor
HGNC, UniProt	CRHR1, P34998	CRHR2, Q13324
Endogenous agonists	–	urocortin 2 (UCN2, Q96RP3) [410], urocortin 3 (UCN3, Q969E3) [410]
Antagonists	SSR125543A (p*K*_i_ 8.7) [698]	–
Selective antagonists	CP 154,526 (pIC_50_ 9.3–10.4) [1218] – Rat, DMP696 (p*K*_i_ 8.3–9) [760], NBI27914 (p*K*_i_ 8.3–9) [314], R121919 (p*K*_i_ 8.3–9) [2227], antalarmin (p*K*_i_ 8.3–9) [2087], CP376395 (pIC_50_ 8.3) [322] – Rat, CRA1000 (pIC_50_ 6.4–7.1) [298]	antisauvagine (p*K*_d_ 8.8–9.6) [412], K41498 (p*K*_i_ 9.2) [1105], K31440 (p*K*_i_ 8.7–8.8) [1697]

### Comments

A CRF binding protein has been identified (CRHBP, P24387) to which both corticotrophin‐releasing hormone(CRH, P06850) and urocortin 1(UCN, P55089) bind with high affinities, which has been suggested to bind and inactivate circulating corticotrophin‐releasing hormone(CRH, P06850) [1558].

### Further reading on Corticotropin‐releasing factor receptors

Grammatopoulos DK. (2012) Insights into mechanisms of corticotropin‐releasing hormone receptor signal transduction. Br. J. Pharmacol. 166: 85‐97 [PMID:21883143]

Gysling K. (2012) Relevance of both type‐1 and type‐2 corticotropin releasing factor receptors in stress‐induced relapse to cocaine seeking behaviour. Biochem. Pharmacol. 83: 1‐5 [PMID:21843515]

Hauger RL et al. (2003) International Union of Pharmacology. XXXVI. Current status of the nomenclature for receptors for corticotropin‐releasing factor and their ligands. Pharmacol Rev. 55: 21‐26 [PMID:12615952]

Valentino RJ et al. (2013) Sex‐biased stress signaling: the corticotropin‐releasing factor receptor as a model. Mol. Pharmacol. 83: 737‐45 [PMID:23239826]

Zhu H et al. (2011) Corticotropin‐releasing factor family and its receptors: pro‐inflammatory or anti‐inflammatory targets in the periphery? Inflamm. Res. 60: 715‐21 [PMID:21476084]

## Dopamine receptors

### Overview

Dopamine receptors (nomenclature as agreed by the NC‐IUPHAR Subcommittee on Dopamine Receptors [1748]) are commonly divided into D_1_‐like (D_1_ and D_5_) and D_2_‐like (D_2_, D_3_ and D_4_) families, where the endogenous agonist is dopamine.

**Table:** 

Nomenclature	D_1_ receptor	D_2_ receptor
HGNC, UniProt	DRD1, P21728	DRD2, P14416
Sub/family‐selective labelled ligands	[^125^ I]SCH23982 (Antagonist) (p*K*_d_ 9.5) [433], [^3^ H]SCH‐23390 (Antagonist) (p*K*_d_ 9.5) [2221]	[^3^ H]spiperone (Antagonist) (p*K*_d_ 10.2) [246, 805, 2219] – Rat
Endogenous agonists	dopamine [1897, 1962]	dopamine [252, 573, 1725]
Agonists	fenoldopam [1962]	rotigotine [448], cabergoline (Partial agonist) [1337], aripiprazole (Partial agonist) [2199], bromocriptine [573, 1337, 1725], MLS1547 (Biased agonist) [572], ropinirole [766], apomorphine (Partial agonist) [252, 573, 1337, 1725, 1844], pramipexole [1332, 1725], benzquinamide [677]
Sub/family‐selective agonists	A68930 [1445], SKF‐38393 (Partial agonist) [1897, 1962]	quinpirole [252, 1332, 1539, 1844, 1846, 2019]
Selective agonists	SKF‐83959 (Biased agonist) [377], SKF‐81297 [47] – Rat	sumanirole [1301]
Antagonists	flupentixol (p*K*_i_ 7–8.4) [1897, 1962]	blonanserin (p*K*_i_ 9.9) [1487], pipotiazine (p*K*_i_ 9.7) [1845], perphenazine (p*K*_i_ 8.9–9.6) [1055, 1761], risperidone (p*K*_i_ 9.4) [64], perospirone (p*K*_i_ 9.2) [1762], trifluoperazine (p*K*_i_ 8.9–9) [1055, 1763]
Sub/family‐selective antagonists	SCH‐23390 (p*K*_i_ 7.4–9.5) [1897, 1962], SKF‐83566 (p*K*_i_ 9.5) [1897], ecopipam (p*K*_i_ 8.3) [1963]	haloperidol (p*K*_i_ 7.4–8.8) [573, 1230, 1332, 1844, 1963]
Selective antagonists	–	L‐741,626 (p*K*_i_ 7.9–8.5) [688, 1069], domperidone (p*K*_i_ 7.9–8.4) [573, 1844], raclopride (p*K*_i_ 8) [1339], ML321 (p*K*_i_ 7) [2147, 2148]
Labelled ligands	–	[^3^ H]raclopride (Antagonist) (p*K*_d_ 8.9) [1081] – Rat

**Table:** 

Nomenclature	D_3_ receptor	D_4_ receptor	D_5_ receptor
HGNC, UniProt	DRD3, P35462	DRD4, P21917	DRD5, P21918
Sub/family‐selective labelled ligands	–	[^3^ H]spiperone (Antagonist) (p*K*_d_ 9.5) [786, 2019]	[^3^ H]SCH‐23390 (Antagonist) (p*K*_d_ 9.2) [1654]
Endogenous agonists	dopamine [252, 573, 1725, 1846]	dopamine [2019]	dopamine [1897]
Agonists	pramipexole [1332, 1725], bromocriptine (Partial agonist) [573, 1337, 1725], ropinirole [766], apomorphine (Partial agonist) [252, 573, 1337, 1725, 1844]	apomorphine (Partial agonist) [1337]	–
Sub/family‐selective agonists	quinpirole [252, 1332, 1339, 1539, 1725, 1844, 1846, 2019]	quinpirole [1337, 1539, 2019]	A68930 [1445]
Selective agonists	PD 128907 [1610, 1725]	PD168,077 (Partial agonist) [1040] – Rat, A412997 [1373] – Rat, A412997 [1373]	–
Antagonists	perospirone (p*K*_i_ 9.6) [1844], sertindole (p*K*_i_ 8–8.8) [64, 1746, 1761], prochlorperazine (p*K*_i_ 8.4) [71], (‐)‐sulpiride (p*K*_i_ 6.7–7.7) [573, 1844, 1934], loxapine (p*K*_i_ 7.7) [1761], domperidone (p*K*_i_ 7.1–7.6) [573, 1844], promazine (p*K*_i_ 6.8) [253]	perospirone (p*K*_i_ 10.1) [1764], sertindole (p*K*_i_ 7.8–9.1) [253, 1761, 1763, 1764], sonepiprazole (p*K*_i_ 8.9) [1739], loxapine (p*K*_i_ 8.1) [1763]	–
Sub/family‐selective antagonists	haloperidol (p*K*_i_ 7.5–8.6) [573, 1782, 1844, 1963]	haloperidol (p*K*_i_ 8.7–8.8) [1088, 1782, 1963]	SCH‐23390 (p*K*_i_ 7.5–9.5) [1897], SKF‐83566 (p*K*_i_ 9.4) [1897], ecopipam (p*K*_i_ 8.3) [1897]
Selective antagonists	S33084 (p*K*_i_ 9.6) [1336], nafadotride (p*K*_i_ 9.5) [1726], PG01037 (p*K*_i_ 9.2) [689], NGB 2904 (p*K*_i_ 8.8) [2143], SB 277011‐A (p*K*_i_ 8) [1641], (+)‐S‐14297 (p*K*_i_ 6.9–7.9) [1334, 1339]	L745870 (p*K*_i_ 9.4) [1069], A‐381393 (p*K*_i_ 8.8) [1420], L741742 (p*K*_i_ 8.5) [1683], ML398 (p*K*_i_ 7.4) [142]	–
Selective allosteric modulators	SB269652 (Negative) (p*K*_i_∼9) [588]	–	–
Labelled ligands	[^3^ H]spiperone (Antagonist) (p*K*_d_ 9.9) [805, 2219] – Rat, [^3^ H]7‐OH‐DPAT (Agonist) [1655], [^3^ H]PD128907 (Agonist) [27]	[^125^ I]L750667 (Antagonist) (p*K*_d_ 9.8) [1539], [^3^ H]NGD941 (Antagonist) (p*K*_d_ 8.3) [1604]	[^125^ I]SCH23982 (Antagonist) (p*K*_d_ 9.1)

### Comments

The selectivity of many of these agents is less than two orders of magnitude. [^3^ H]raclopride exhibits similar high affinity for D_2_ and D_3_ receptors (low affinity for D_4_), but has been used to label D_2_ receptors in the presence of a D_3_‐selective antagonist. [^3^ H]7‐OH‐DPAT has similar affinity for D_2_ and D_3_ receptors, but labels only D_3_ receptors in the absence of divalent cations. The pharmacological profile of the D_5_ receptor is similar to, yet distinct from, that of the D_1_ receptor. The splice variants of the D_2_ receptor are commonly termed D_2S_ and D_2L_ (short and long). The DRD4 gene encoding the D_4_ receptor is highly polymorphic in humans, with allelic variations of the protein from amino acid 387 to 515.

### Further reading on Dopamine receptors

Beaulieu JM et al. (2015) Dopamine receptors ‐ IUPHAR Review 13. Br. J. Pharmacol. 172: 1‐23 [PMID:25671228]

Beaulieu JM et al. (2011) The physiology, signaling, and pharmacology of dopamine receptors. Pharmacol. Rev. 63: 182‐217 [PMID:21303898]

Cumming P. (2011) Absolute abundances and affinity states of dopamine receptors in mammalian brain: A review. Synapse 65: 892‐909 [PMID:21308799]

Maggio R et al. (2010) Dopamine D2‐D3 receptor heteromers: pharmacological properties and therapeutic significance. Curr Opin Pharmacol 10: 100‐7 [PMID:19896900]

Ptácek R et al. (2011) Dopamine D4 receptor gene DRD4 and its association with psychiatric disorders. Med. Sci. Monit. 17: RA215‐20 [PMID:21873960]

Schwartz J‐C et al.. (1998) Dopamine Receptors. In The IUPHAR Compendium of Receptor Characterization and Classification Edited by Girdlestone D: IUPHAR Media: 141‐151

Undieh AS. (2010) Pharmacology of signaling induced by dopamine D(1)‐like receptor activation. Pharmacol. Ther. 128: 37‐60 [PMID:20547182]

## Endothelin receptors

### Overview

Endothelin receptors (nomenclature as agreed by the NC‐IUPHAR Subcommittee on Endothelin Receptors [413]) are activated by the endogenous 21 amino‐acid peptides endothelins 1‐3 (endothelin‐1(EDN1, P05305), endothelin‐2(EDN2, P20800) and endothelin‐3(EDN3, P14138)).

**Table:** 

Nomenclature	ET_A_ receptor	ET_B_ receptor
HGNC, UniProt	EDNRA, P25101	EDNRB, P24530
Family selective agonists	–	endothelin‐1 (EDN1, P05305) = endothelin‐2 (EDN2, P20800), endothelin‐3 (EDN3, P14138)
Potency order of endogenous ligands	endothelin‐1 (EDN1, P05305) = endothelin‐2 (EDN2, P20800) >endothelin‐3 (EDN3, P14138) [1242]	–
Selective agonists	–	sarafotoxin S6c [1062, 1690], BQ 3020 [1650], [Ala ^1,3,11,15^]ET‐1 [1354], IRL 1620 [2078]
Sub/family‐selective antagonists	SB209670 (p*K*_B_ 9.4) [502] – Rat, TAK 044 (pA_2_ 8.4) [2081] – Rat, bosentan (pA_2_ 7.2) [367] – Rat	SB209670 (p*K*_B_ 9.4) [502] – Rat, TAK 044 (pA_2_ 8.4) [2081] – Rat, bosentan (p*K*_i_ 7.1) [1405]
Selective antagonists	macitentan (pIC_50_ 9.3) [177], sitaxsentan (pA_2_ 8) [2135], FR139317 (Inverse agonist) (pIC_50_ 7.3–7.9) [1242], BQ123 (pA_2_ 6.9–7.4) [1242], ambrisentan (pA_2_ 7.1) [178]	A192621 (p*K*_d_ 8.1) [2043], BQ788 (p*K*_d_ 7.9–8) [1690], IRL 2500 (p*K*_d_ 7.2) [1690], Ro 46‐8443 (pIC_50_ 7.2) [215]
Labelled ligands	[^125^ I]PD164333 (Antagonist) (p*K*_d_ 9.6–9.8) [416], [^3^ H]S0139 (Antagonist) (p*K*_d_ 9.2), [^125^ I]PD151242 (Antagonist) (p*K*_d_ 9–9.1) [417], [^3^ H]BQ123 (Antagonist) (p*K*_d_ 8.5) [858]	[^125^ I]IRL1620 (Agonist) [1421], [^125^ I]BQ3020 (Agonist) [737, 1354, 1565], [^125^ I][Ala ^1,3,11,15^]ET‐1 (Agonist) [1354]

### Comments

Splice variants of the ET_A_ receptor have been identified in rat pituitary cells; one of these, ET_A_R‐C13, appeared to show loss of function with comparable plasma membrane expression to wild type receptor [748]. Subtypes of the ET_B_ receptor have been proposed, although gene disruption studies in mice suggest that only a single gene product exists [1350].

### Further reading on Endothelin receptors

Clozel M et al. (2013) Endothelin receptor antagonists. Handb Exp Pharmacol 218: 199‐227 [PMID:24092342]

Davenport AP. (2002) International Union of Pharmacology. XXIX. Update on endothelin receptor nomenclature. Pharmacol. Rev. 54: 219‐26 [PMID:12037137]

Davenport AP et al. (2016) Endothelin. Pharmacol. Rev. 68: 357‐418 [PMID:26956245]

Maguire JJ et al. (2014) Endothelin@25 ‐ new agonists, antagonists, inhibitors and emerging research frontiers: IUPHAR Review 12. Br. J. Pharmacol. 171: 5555‐72 [PMID:25131455]

## G protein‐coupled estrogen receptor

### Overview

The G protein‐coupled estrogen receptor (GPER, nomenclature as agreed by the NC‐IUPHAR Subcommittee on the G protein‐coupled estrogen receptor [1607]) was identified following observations of estrogen‐evoked cyclic AMP signalling in breast cancer cells [65], which mirrored the differential expression of an orphan 7‐transmembrane receptor GPR30 [276]. There are observations of both cell‐surface and intracellular expression of the GPER receptor [1647, 1953].

**Table:** 

Nomenclature	GPER
HGNC, UniProt	GPER1, Q99527
Agonists	raloxifene [1570]
Selective agonists	G1 [179]
Selective antagonists	G36 (pIC_50_ 6.8–6.9) [438], G15 (pIC_50_ 6.7) [437]
Labelled ligands	[^3^ H]17 *β*‐estradiol (Agonist) [1953]

### Comments

Antagonists at the nuclear estrogen receptor, such as fulvestrant, tamoxifen[540] and raloxifene[1570], as well as the flavonoid ‘phytoestrogens’ genistein and quercetin[1241], are agonists at GPER receptors. A complete review of GPER pharmacology has been recently published [1607].

### Further reading on G protein‐coupled estrogen receptor

Prossnitz ER et al. (2015) International Union of Basic and Clinical Pharmacology. XCVII. G Protein‐Coupled Estrogen Receptor and Its Pharmacologic Modulators. Pharmacol. Rev. 67: 505‐40 [PMID:26023144]

Prossnitz ER et al. (2015) What have we learned about GPER function in physiology and disease from knockout mice? J. Steroid Biochem. Mol. Biol. 153: 114‐26 [PMID:26189910]

## Formylpeptide receptors

### Overview

The formylpeptide receptors (nomenclature agreed by the NC‐IUPHAR Subcommittee on the formylpeptide receptor family [2180]) respond to exogenous ligands such as the bacterial product fMet‐Leu‐Phe (fMLP) and endogenous ligands such as annexin I(ANXA1, P04083) , cathepsin G(CTSG, P08311), amyloid *β*42, serum amyloid A and spinorphin, derived from *β* ‐haemoglobin(HBB, P68871).

**Table:** 

Nomenclature	FPR1	FPR2/ALX	FPR3
HGNC, UniProt	FPR1, P21462	FPR2, P25090	FPR3, P25089
Potency order of endogenous ligands	fMet‐Leu‐Phe>cathepsin G (CTSG, P08311) >annexin I (ANXA1, P04083) [1118, 1895]	LXA_4_ = aspirin triggered lipoxin A4 = ATLa2 = resolvin D1>LTC_4_ = LTD_4_≫15‐deoxy‐LXA4≫fMet‐Leu‐Phe [365, 544, 546, 684, 1919]	–
Endogenous agonists	–	LXA_4_ [1052], resolvin D1 [1052], aspirin‐triggered resolvin D1 [1051], aspirin triggered lipoxin A4	F2L (HEBP1, Q9NRV9) [1333]
Agonists	fMet‐Leu‐Phe [575, 1802]	–	–
Selective agonists	–	ATLa2 [697]	–
Endogenous antagonists	spinorphin (pIC_50_ 4.3) [1165, 1404]	–	–
Antagonists	t‐Boc‐FLFLF (p*K*_i_ 6–6.5) [2095]	–	–
Selective antagonists	cyclosporin H (p*K*_i_ 6.1–7.1) [2095, 2167]	WRWWWW (pIC_50_ 6.6) [83], t‐Boc‐FLFLF (pIC_50_ 4.3–6) [574, 1867, 2061]	–
Labelled ligands	[^3^ H]fMet‐Leu‐Phe (Agonist) [1036]	[^3^ H]LXA_4_ (Agonist) [544, 545]	–
Comments	A FITC‐conjugated fMLP analogue has been used for binding to the mouse recombinant receptor [758].	–	–

### Comments

Note that the data for FPR2/ALX are also reproduced on the leukotriene receptor page.

### Further reading on Formylpeptide receptors

Dorward DA et al. (2015) The Role of Formylated Peptides and Formyl Peptide Receptor 1 in Governing Neutrophil Function during Acute Inflammation. Am. J. Pathol. 185: 1172‐1184 [PMID:25791526]

Dufton N et al. (2010) Therapeutic anti‐inflammatory potential of formyl‐peptide receptor agonists. Pharmacol. Ther. 127: 175‐88 [PMID:20546777]

Liu M et al. (2012) G protein‐coupled receptor FPR1 as a pharmacologic target in inflammation and human glioblastoma. Int. Immunopharmacol. 14: 283‐8 [PMID:22863814]

Rabiet MJ et al. (2011) N‐formyl peptide receptor 3 (FPR3) departs from the homologous FPR2/ALX receptor with regard to the major processes governing chemoattractant receptor regulation, expression at the cell surface, and phosphorylation. J. Biol. Chem. 286: 26718‐31 [PMID:21543323]

Yazid S et al. (2012) Anti‐inflammatory drugs, eicosanoids and the annexin A1/FPR2 anti‐inflammatory system. Prostaglandins Other Lipid Mediat. 98: 94‐100 [PMID:22123264]

Ye RD et al. (2009) International Union of Basic and Clinical Pharmacology. LXXIII. Nomenclature for the formyl peptide receptor (FPR) family. Pharmacol. Rev. 61: 119‐61 [PMID:19498085]

## Free fatty acid receptors

### Overview

Free fatty acid receptors (FFA, nomenclature as agreed by the NC‐IUPHAR Subcommittee on free fatty acid receptors [414, 1876]) are activated by free fatty acids. Long‐chain saturated and unsaturated fatty acids (C14.0 (myristic acid), C16:0 (palmitic acid), C18:1 (oleic acid), C18:2 (linoleic acid), C18:3, (*α* ‐linolenic acid), C20:4 (arachidonic acid), C20:5,n‐3 (EPA) and C22:6,n‐3 (docosahexaenoic acid)) activate FFA1 [223, 872, 1043] and FFA4 receptors [795, 852, 1494], while short chain fatty acids (C2 (acetic acid), C3 (propanoic acid), C4 (butyric acid) and C5 (pentanoic acid)) activate FFA2 [231, 1117, 1465] and FFA3 [231, 1117] receptors. The crystal structure for agonist bound FFA1 has been described [1862].

**Table:** 

Nomenclature	FFA1 receptor	FFA2 receptor
HGNC, UniProt	FFAR1, O14842	FFAR2, O15552
Endogenous agonists	docosahexaenoic acid [223, 872], *α* ‐linolenic acid [223, 872, 1043], oleic acid [223, 872, 1043], myristic acid [223, 872, 1043]	propanoic acid [231, 1117, 1465, 1741], acetic acid [231, 1117, 1465, 1741], butyric acid [231, 1117, 1465, 1741], trans‐2‐methylcrotonic acid [1741], 1‐methylcyclopropanecarboxylic acid [1741]
Selective agonists	AMG‐837 [1176], compound 4 [347], TUG‐770 [346], TUG‐905 [345], GW9508 (Partial agonist) [222], fasiglifam [935, 1434, 1862, 1985]	compound 1 [840] – Rat
Selective antagonists	GW1100 (pIC_50_ 6) [222, 1875]	GLPG0974 (pIC_50_ 8.1) [1423, 1584], CATPB (pIC_50_ 6.5) [841]
Comments	Antagonist GW1100 is also an oxytocin receptor antagonist [222]. Fasiglifam, TUG‐770 and GW9508 are approximately 100 fold selective for FFA1 over FFA4 [222, 346, 1434]. AMG‐837 and the related analogue AM6331 have been suggested to have an allosteric mechanism of action at FFA1, with respect to the orthosteric fatty acid binding site [1176, 2153].	–

**Table:** 

Nomenclature	FFA3 receptor	FFA4 receptor	GPR42
HGNC, UniProt	FFAR3, O14843	FFAR4, Q5NUL3	GPR42, O15529
Endogenous agonists	propanoic acid [231, 1117, 1741, 2152], butyric acid [231, 1117, 1741, 2152], 1‐methylcyclopropanecarboxylic acid [1741]	*α* ‐linolenic acid [1794], myristic acid [2084], *α* ‐linolenic acid [1932] – Rat, oleic acid [2084]	–
Agonists	acetic acid [231, 1117, 1741, 2152]	–	–
Selective agonists	–	compound A [1493], TUG‐891 [1794], NCG21 [1902]	–
Comments	Beta‐hydroxybutyrate has been reported to antagonise FFA3 responses to short chain fatty acids [997]. A range of FFA3 selective molecules with agonist and antagonist properties, but which bind at sites distinct from the short chain fatty acid binding site (i.e. allosteric modulators), have recently been described [180, 839, 1226].	A wide range of both saturated and unsaturated fatty acids containing from 6 to 22 carbons have been shown to act as agonists at FFA4 [348] with a small subset listed above. Compound A [PMID 24997608] exhibits more than 1000 fold selectivity [1493], and TUG‐891 50‐1000 fold selectivity for FFA4 over FFA1 [1794], dependent on the assay. NGC21 exhibits approximately 15 fold selectivity for FFA4 over FFA1 [1894].	–

### Comments

Short (361 amino acids) and long (377 amino acids) splice variants of human FFA4 have been reported [1372], which differ by a 16 amino acid insertion in intracellular loop 3, and exhibit differences in intracellular signalling properties in recombinant systems [2084]. The long FFA4 splice variant has not been identified in other primates or rodents to date [795, 1372].

GPR42 was originally described as a pseudogene within the family (ENSFM00250000002583), but the discovery of several polymorphisms suggests that some versions of GPR42 may be functional [1167]. GPR84 is a structurally‐unrelated G protein‐coupled receptor which has been found to respond to medium chain fatty acids [2067].

### Further reading on Free fatty acid receptors

Bolognini D et al. (2016) The Pharmacology and Function of Receptors for Short‐Chain Fatty Acids. Mol. Pharmacol. 89: 388‐98 [PMID:26719580]

Mancini AD et al. (2013) The fatty acid receptor FFA1/GPR40 a decade later: how much do we know? Trends Endocrinol. Metab. 24: 398‐407 [PMID:23631851]

Moniri NH. (2016) Free‐fatty acid receptor‐4 (GPR120): Cellular and molecular function and its role in metabolic disorders. Biochem. Pharmacol. 110‐111: 1‐15 [PMID:26827942]

Stoddart LA et al. (2008) International Union of Pharmacology. LXXI. Free fatty acid receptors FFA1, ‐2, and ‐3: pharmacology and pathophysiological functions. Pharmacol. Rev. 60: 405‐17 [PMID:19047536]

Talukdar S et al. (2011) Targeting GPR120 and other fatty acid‐sensing GPCRs ameliorates insulin resistance and inflammatory diseases. Trends Pharmacol. Sci. 32: 543‐50 [PMID:21663979]

Watterson KR et al. (2014) Treatment of type 2 diabetes by free Fatty Acid receptor agonists. Front Endocrinol (Lausanne) 5: 137 [PMID:25221541]

## GABA_B_ receptors

### Overview

Functional GABA_B_ receptors (nomenclature as agreed by the NC‐IUPHAR Subcommittee on GABA_B_ receptors [199, 1579]) are formed from the heterodimerization of two similar 7TM subunits termed GABA_B1_ and GABA_B2_[199, 506, 1578, 1579, 2002]. GABA_B_ receptors are widespread in the CNS and regulate both pre‐ and postsynaptic activity. The GABA_B1_ subunit, when expressed alone, binds both antagonists and agonists, but the affinity of the latter is generally 10‐100‐fold less than for the native receptor. Co‐expression of GABA_B1_ and GABA_B2_ subunits allows transport of GABA_B1_ to the cell surface and generates a functional receptor that can couple to signal transduction pathways such as high‐voltage‐activated Ca^2+^ channels (Ca_v_2.1, Ca_v_2.2), or inwardly rectifying potassium channels (Kir3) [147, 199, 200]. The GABA_B1_ subunit harbours the GABA (orthosteric)‐binding site within an extracellular domain (ECD) venus flytrap module (VTM), whereas the GABA_B2_ subunit mediates G protein‐coupled signalling [199, 622, 624, 1578]. The two subunits interact by direct allosteric coupling [1367], such that GABA_B2_ increases the affinity of GABA_B1_ for agonists and reciprocally GABA_B1_ facilitates the coupling of GABA_B2_ to G proteins [622, 1060, 1578]. GABA_B1_ and GABA_B2_ subunits assemble in a 1:1 stoichiometry by means of a coiled‐coil interaction between *α*‐helices within their carboxy‐termini that masks an endoplasmic reticulum retention motif (RXRR) within the GABA_B1_ subunit but other domains of the proteins also contribute to their heteromerization [147, 250, 1578]. Recent evidence indicates that higher order assemblies of GABA_B_receptor comprising dimers of heterodimers occur in recombinant expression systems and in vivo and that such complexes exhibit negative functional cooperativity between heterodimers [373, 1577]. Adding further complexity, KCTD (potassium channel tetramerization proteins) 8, 12, 12b and 16 associate as tetramers with the carboxy terminus of the GABA_B2_ subunit to impart altered signalling kinetics and agonist potency to the receptor complex [108, 1751, 1990] and are reviewed by [1580]. The molecular complexity of GABAB receptors is further increased through association with trafficking and effector proteins [Schwenk et al., 2016, Nature Neuroscience 19(2): 233‐42] and reviewed by [1576]. Four isoforms of the human GABA_B1_ subunit have been cloned. The predominant GABA_B1a_ and GABA_B1b_ isoforms, which are most prevalent in neonatal and adult brain tissue respectively, differ in their ECD sequences as a result of the use of alternative transcription initiation sites. GABA_B1a_‐containing heterodimers localise to distal axons and mediate inhibition of glutamate release in the CA3‐CA1 terminals, and GABA release onto the layer 5 pyramidal neurons, whereas GABA_B1b_‐containing receptors occur within dendritic spines and mediate slow postsynaptic inhibition [1613, 2035]. Only the 1a and 1b variants are identified as components of native receptors [199]. Additional GABA_B1_ subunit isoforms have been described in rodents and humans [1130] and reviewed by [147].

**Table:** 

Nomenclature	GABA_B_ receptor
Subunits	kctd12b (Accessory protein), KCTD16 (Accessory protein), KCTD12 (Accessory protein), GABA_B2_, GABA_B1_, KCTD8 (Accessory protein)
Agonists	CGP 44532 [581] – Rat, (‐)‐baclofen [581] – Rat, 3‐APPA [800], baclofen [800, 2130], 3‐APMPA [2130]
Antagonists	CGP 62349 (p*K*_i_ 8.5–8.9) [800, 2130], CGP 55845 (p*K*_i_ 7.8) [2130], SCH 50911 (p*K*_i_ 5.5–6) [800, 2130], CGP 35348 (p*K*_i_ 4.4) [2130], 2‐hydroxy‐saclofen (pIC_50_ 4.1) [957] – Rat
Labelled ligands	[^3^ H]CGP 54626 (Antagonist) (p*K*_i_ 9.1) [922] – Rat, [^3^ H]CGP 62349 (Antagonist) (p*K*_d_ 9.1) [964] – Rat, [^125^ I]CGP 64213 (Antagonist) (p*K*_d_ 9) [594] – Rat, [^125^ I]CGP 71872 (Antagonist) (p*K*_d_ 9) [957] – Rat, [^3^ H](R)‐(‐)‐baclofen (Agonist)

### Subunits

**Table:** 

Nomenclature	GABA_B1_	GABA_B2_
HGNC, UniProt	GABBR1, Q9UBS5	GABBR2, O75899

### Comments

Potencies of agonists and antagonists listed in the table, quantified as IC_50_ values for the inhibition of [^3^ H]CGP27492 binding to rat cerebral cortex membranes, are from [199, 580, 581]. Radioligand K_D_ values relate to binding to rat brain membranes. CGP 71872 is a photoaffinity ligand for the GABA_B1_ subunit [128]. CGP27492 (3‐APPA), CGP35024 (3‐APMPA) and CGP 44532 act as antagonists at human GABA_A_ *ρ*1 receptors, with potencies in the low micromolar range [580]. In addition to the ligands listed in the table, Ca ^2+^ binds to the VTM of the GABA_B1_ subunit to act as a positive allosteric modulator of GABA[594]. Synthetic positive allosteric modulators with low, or no, intrinsic activity include CGP7930, GS39783, BHF‐177[2040] and (+)‐BHFF[9, 147, 154, 580]. The site of action of CGP7930 and GS39783 appears to be on the heptahelical domain of the GABA_B2_ subunit [483, 1578]. In the presence of CGP7930 or GS39783, CGP 35348 and 2‐hydroxy‐saclofen behave as partial agonists [580]. A negative allosteric modulator of GABA_B_ activity has been reported [318]. Knock‐out of the GABA_B1_ subunit in C57B mice causes the development of severe tonic‐clonic convulsions that prove fatal within a month of birth, whereas GABA_B1_^‐/‐^ BALB/c mice, although also displaying spontaneous epileptiform activity, are viable. The phenotype of the latter animals additionally includes hyperalgesia, hyperlocomotion (in a novel, but not familiar, environment), hyperdopaminergia, memory impairment and behaviours indicative of anxiety [510, 2008]. A similar phenotype has been found for GABA_B2_^‐/‐^ BALB/c mice [613].

### Further reading on GABA_B_ receptors

Bowery NG et al. (2002) International Union of Pharmacology. XXXIII. Mammalian gamma‐aminobutyricacid(B) receptors: structure and function. Pharmacol Rev. 54: 247‐264 [PMID:12037141]

Froestl W. (2011) An historical perspective on GABAergic drugs. Future Med Chem 3: 163‐75 [PMID:21428811]

Gassmann M et al. (2012) Regulation of neuronal GABA(B) receptor functions by subunit composition. Nat. Rev. Neurosci. 13: 380‐94 [PMID:22595784]

Pin JP et al. (2016) Organization and functions of mGlu and GABAB receptor complexes. Nature 540: 60‐68 [PMID:27905440]

## Galanin receptors

### Overview

Galanin receptors (provisional nomenclature as recommended by NC‐IUPHAR[557]) are activated by the endogenous peptides galanin(GAL, P22466) and galanin‐like peptide(GALP, Q9UBC7). Human galanin(GAL, P22466) is a 30 amino‐acid non‐amidated peptide [525]; in other species, it is 29 amino acids long and C‐terminally amidated. Amino acids 1–14 of galanin are highly conserved in mammals, birds, reptiles, amphibia and fish. Shorter peptide species (e.g. human galanin‐1–19 [143] and porcine galanin‐5–29 [1809]) and N‐terminally extended forms (e.g. N‐terminally seven and nine residue elongated forms of porcine galanin [144, 1809]) have been reported.

**Table:** 

Nomenclature	GAL_1_ receptor	GAL_2_ receptor	GAL_3_ receptor
HGNC, UniProt	GALR1, P47211	GALR2, O43603	GALR3, O60755
Potency order of endogenous ligands	galanin (GAL, P22466) >galanin‐like peptide (GALP, Q9UBC7) [1500]	galanin‐like peptide (GALP, Q9UBC7) ≥galanin (GAL, P22466) [1500]	galanin‐like peptide (GALP, Q9UBC7) >galanin (GAL, P22466) [1095]
Agonists	–	galanin(2‐29) (rat/mouse) [1526, 2069, 2070, 2071] – Rat	–
Selective agonists	–	[D‐Trp ^2^]galanin‐(1‐29) [1834] – Rat	–
Selective antagonists	2,3‐dihydro‐1,4‐dithiin‐1,1,4,4‐tetroxide (pIC_50_ 5.6) [1758]	M871 (p*K*_i_ 7.9) [1848]	SNAP 398299 (p*K*_i_ 8.3) [1033, 1034, 1906], SNAP 37889 (p*K*_i_ 7.8–7.8) [1033, 1034, 1906]
Selective allosteric modulators	–	CYM2503 (Positive) (pEC_50_ 9.2) [1213] – Rat	–
Labelled ligands	[^125^ I][Tyr ^26^]galanin (human) (Agonist) [552], [^125^I][Tyr^26^ ]galanin (human) (Agonist) [552]	[^125^ I][Tyr ^26^]galanin (human) (Agonist) [2070] – Rat	[^125^ I][Tyr ^26^]galanin (pig) (Agonist) [191, 1835]
Comments	–	The CYM2503 PAM potentiates the anticonvulsant activity of endogenous galanin in mouse seizure models [1213].	–

### Comments

galanin‐(1‐11) is a high‐affinity agonist at GAL_1_/GAL_2_(pK_i_ 9), and galanin(2‐11) is selective for GAL_2_ and GAL_3_ compared with GAL_1_[1212]. [^125^I]‐[Tyr^26^]galanin binds to all three subtypes with K_d_ values generally reported to range from 0.05 to 1 nM, depending on the assay conditions used [552, 1821, 1834, 1835, 2070]. Porcine galanin‐(3‐29) does not bind to cloned GAL_1_, GAL_2_ or GAL_3_ receptors, but a receptor that is functionally activated by porcine galanin‐(3–29) has been reported in pituitary and gastric smooth muscle cells [691, 2142]. Additional galanin receptor subtypes are also suggested from studies with chimeric peptides (e.g. M15, M35 and M40), which act as antagonists in functional assays in the cardiovascular system [2000], spinal cord [2114], locus coeruleus, hippocampus [106] and hypothalamus [107, 1142], but exhibit agonist activity at some peripheral sites [107, 691]. The chimeric peptides M15, M32, M35, M40 and C7 are agonists at GAL_1_ receptors expressed endogenously in Bowes human melanoma cells [1500], and at heterologously expressed recombinant GAL_1_, GAL_2_ and GAL_3_ receptors [552, 1834, 1835]. Recent studies have described the synthesis of a series of novel, systemically‐active, galanin analogues, with modest preferential binding at the GAL_2_ receptor. Specific chemical modifications to the galanin backbone increased brain levels of these peptides after i.v. injection and several of these peptides exerted a potent antidepressant‐like effect in mouse models of depression [1698].

### Further reading on Galanin receptors

Foord SM et al. (2005) International Union of Pharmacology. XLVI. G protein‐coupled receptor list. Pharmacol Rev 57: 279‐288 [PMID:15914470]

Lang R et al. (2015) Physiology, signaling, and pharmacology of galanin peptides and receptors: three decades of emerging diversity. Pharmacol. Rev. 67: 118‐75 [PMID:25428932]

Lang R et al. (2011) The galanin peptide family in inflammation. Neuropeptides 45: 1‐8 [PMID:21087790]

Lawrence C et al. (2011) Galanin‐like peptide (GALP) is a hypothalamic regulator of energy homeostasis and reproduction. Front Neuroendocrinol 32: 1‐9 [PMID:20558195]

Webling KE et al. (2012) Galanin receptors and ligands. Front Endocrinol (Lausanne) 3: 146 [PMID:23233848]

## Ghrelin receptor

### Overview

The ghrelin receptor (nomenclature as agreed by the NC‐IUPHAR Subcommittee for the Ghrelin receptor [415]) is activated by a 28 amino‐acid peptide originally isolated from rat stomach, where it is cleaved from a 117 amino‐acid precursor (GHRL, Q9UBU3). The human gene encoding the precursor peptide has 83% sequence homology to rat prepro‐ghrelin, although the mature peptides from rat and human differ by only two amino acids [1285]. Alternative splicing results in the formation of a second peptide, [des‐Gln ^14^]ghrelin(GHRL, Q9UBU3) with equipotent biological activity [822]. A unique post‐translational modification (octanoylation of Ser^3^, catalysed by ghrelin *O*‐acyltransferase (MBOAT4, Q96T53) [2170] occurs in both peptides, essential for full activity in binding to ghrelin receptors in the hypothalamus and pituitary, and for the release of growth hormone from the pituitary [1029]. Structure activity studies showed the first five N‐terminal amino acids to be the minimum required for binding [122], and receptor mutagenesis has indicated overlap of the ghrelin binding site with those for small molecule agonists and allosteric modulators of ghrelin(GHRL, Q9UBU3) function [814]. In cell systems, the ghrelin receptor is constitutively active [815], but this is abolished by a naturally occurring mutation (A204E) that results in decreased cell surface receptor expression and is associated with familial short stature [1527].

**Table:** 

Nomenclature	ghrelin receptor
HGNC, UniProt	GHSR, Q92847
Potency order of endogenous ligands	ghrelin (GHRL, Q9UBU3) = [des‐Gln ^14^]ghrelin (GHRL, Q9UBU3) [121, 1285]
Selective antagonists	GSK1614343 (pIC_50_ 8.4) [1699], GSK1614343 (p*K*_B_ 8) [1556] – Rat
Labelled ligands	[^125^ I][His ^9^]ghrelin (human) (Agonist) [956], [^125^ I][Tyr ^4^]ghrelin (human) (Agonist) [1394]

### Comments

[des‐octanoyl]ghrelin(GHRL, Q9UBU3) has been shown to bind (as [^125^I]Tyr^4^ ‐des‐octanoyl‐ghrelin) and have effects in the cardiovascular system [121], which raises the possible existence of different receptor subtypes in peripheral tissues and the central nervous system. A potent inverse agonist has been identified ([D‐Arg ^1^,D‐Phe^5^,D‐Trp^7,9^,Leu^11^]substance P, pD_2_ 8.3; [812]). Ulimorelin, described as a ghrelin receptor agonist (pK_i_ 7.8 and pD_2_ 7.5 at human recombinant ghrelin receptors), has been shown to stimulate ghrelin receptor mediated food intake and gastric emptying but not elicit release of growth hormone, or modify ghrelin stimulated growth hormone release, thus pharmacologically discriminating the orexigenic and gastrointestinal actions of ghrelin(GHRL, Q9UBU3) from the release of growth hormone [567]. A number of selective antagonists have been reported, including peptidomimetic [1393] and non‐peptide small molecules including GSK1614343[1556, 1699].

### Further reading on Ghrelin receptor

Andrews ZB. (2011) The extra‐hypothalamic actions of ghrelin on neuronal function. Trends Neurosci. 34: 31‐40 [PMID:21035199]

Angelidis G et al. (2010) Current and potential roles of ghrelin in clinical practice. J. Endocrinol. Invest. 33: 823‐38 [PMID:21293171]

Briggs DI et al. (2011) Metabolic status regulates ghrelin function on energy homeostasis. Neuroendocrinology 93: 48‐57 [PMID:21124019]

Callaghan B et al. (2014) Novel and conventional receptors for ghrelin, desacyl‐ghrelin, and pharmacologically related compounds. Pharmacol. Rev. 66: 984‐1001 [PMID:25107984]

Davenport AP et al. (2005) International Union of Pharmacology. LVI. Ghrelin receptor nomenclature, distribution, and function. Pharmacol. Rev. 57: 541‐6 [PMID:16382107]

## Glucagon receptor family

### Overview

The glucagon family of receptors (nomenclature as agreed by the NC‐IUPHAR Subcommittee on the Glucagon receptor family [1296]) are activated by the endogenous peptide (27‐44 aa) hormones glucagon(GCG, P01275), glucagon‐like peptide 1(GCG, P01275), glucagon‐like peptide 2 (GCG, P01275), glucose‐dependent insulinotropic polypeptide (also known as gastric inhibitory polypeptide(GIP, P09681)), GHRH(GHRH, P01286) and secretin(SCT, P09683). One common precursor (GCG) generates glucagon(GCG, P01275), glucagon‐like peptide 1(GCG, P01275) and glucagon‐like peptide 2(GCG, P01275) peptides [866].

**Table:** 

Nomenclature	GHRH receptor	GIP receptor	GLP‐1 receptor
HGNC, UniProt	GHRHR, Q02643	GIPR, P48546	GLP1R, P43220
Endogenous agonists	–	gastric inhibitory polypeptide (GIP, P09681) [2042]	glucagon‐like peptide 1‐(7‐36) amide (GCG, P01275) [927], glucagon‐like peptide 1‐(7‐37) (GCG, P01275) [449]
Agonists	JI‐38 [265], sermorelin	–	liraglutide [1020], lixisenatide [2097], WB4‐24 [528]
Selective agonists	BIM28011 [393], tesamorelin	–	exendin‐4 [1346], exendin‐4 [927], exendin‐3 (P20394) [1635]
Selective antagonists	JV‐1‐36 (p*K*_i_ 10.1–10.4) [1733, 2026, 2027] – Rat, JV‐1‐38 (p*K*_i_ 10.1) [1733, 2026, 2027] – Rat	[Pro ^3^]GIP [615] – Mouse	exendin‐(9‐39) (p*K*_i_ 8.1) [927], GLP‐1‐(9‐36) (pIC_50_ 6.9) [1368] – Rat, T‐0632 (pIC_50_ 4.7) [1961]
Labelled ligands	[^125^ I]GHRH (human) (Agonist) [196] – Rat	[^125^ I]GIP (human) (Agonist) [593] – Rat	[^125^ I]GLP‐1‐(7‐36)‐amide (Agonist) [927], [^125^ I]exendin‐(9‐39) (Antagonist) (p*K*_d_ 8.3) [927], [^125^ I]GLP‐1‐(7‐37) (human) (Agonist)

**Table:** 

Nomenclature	GLP‐2 receptor	glucagon receptor	secretin receptor
HGNC, UniProt	GLP2R, O95838	GCGR, P47871	SCTR, P47872
Endogenous agonists	glucagon‐like peptide 2 (GCG, P01275) [1958]	glucagon (GCG, P01275) [1587]	secretin (SCT, P09683) [343]
Agonists	teduglutide [1310]	–	–
Selective antagonists	–	L‐168,049 (pIC_50_ 8.4) [282], adomeglivant (p*K*_i_ 8.2) [963, 967], des‐His ^1^‐[Glu^9^]glucagon‐NH_2_ (pA_2_ 7.2) [2004, 2005] – Rat, NNC 92‐1687 (p*K*_i_ 5) [1234], BAY27‐9955 [1566]	[(CH_2_NH)^4,5^]secretin (p*K*_i_ 5.3) [704]
Labelled ligands	–	[^125^ I]glucagon (human, mouse, rat) (Agonist)	[^125^ I](Tyr ^10^)secretin‐27 (rat) (Agonist) [2001] – Rat

### Comments

The glucagon receptor has been reported to interact with receptor activity modifying proteins (RAMPs), specifically RAMP2, in heterologous expression systems [349], although the physiological significance of this has yet to be established.

### Further reading on Glucagon receptor family

Ahrén B. (2015) Glucagon–Early breakthroughs and recent discoveries. Peptides 67: 74‐81 [PMID:25814364]

Campbell JE et al. (2013) Pharmacology, physiology, and mechanisms of incretin hormone action. Cell Metab. 17: 819‐37 [PMID:23684623]

Donnelly D. (2012) The structure and function of the glucagon‐like peptide‐1 receptor and its ligands. Br. J. Pharmacol. 166: 27‐41 [PMID:21950636]

Kazda CM et al. (2016) Evaluation of Efficacy and Safety of the Glucagon Receptor Antagonist LY2409021 in Patients With Type 2 Diabetes: 12‐ and 24‐Week Phase 2 Studies. Diabetes Care 39: 1241‐9 [PMID:26681715]

Mayo KE et al. (2003) International Union of Pharmacology. XXXV. The glucagon receptor family. Pharmacol. Rev. 55: 167‐94 [PMID:12615957]

Trujillo JM et al. (2014) GLP‐1 receptor agonists for type 2 diabetes mellitus: recent developments and emerging agents. Pharmacotherapy 34: 1174‐86 [PMID:25382096]

## Glycoprotein hormone receptors

### Overview

Glycoprotein hormone receptors (provisional nomenclature [557]) are activated by a non‐covalent heterodimeric glycoprotein made up of a common *α* chain (glycoprotein hormone common alpha subunit (CGA, P01215) CGA, P01215), with a unique *β* chain that confers the biological specificity to FSH(CGA FSHB, P01215 P01225), LH(CGA LHB, P01215 P01229), hCG(CGA CGB3, P01215 P01233) or TSH(CGA TSHB, P01215 P01222). There is binding cross‐reactivity across the endogenous agonists for each of the glycoprotein hormone receptors. The deglycosylated hormones appear to exhibit reduced efficacy at these receptors [1701].

**Table:** 

Nomenclature	FSH receptor	LH receptor	TSH receptor
HGNC, UniProt	FSHR, P23945	LHCGR, P22888	TSHR, P16473
Potency order of endogenous ligands	FSH (CGA FSHB, P01215 P01225)	LH (CGA LHB, P01215 P01229), hCG (CGA CGB3, P01215 P01233)	TSH (CGA TSHB, P01215 P01222)
Endogenous agonists	FSH (CGA FSHB, P01215 P01225)	hCG (CGA CGB3, P01215 P01233) [907, 1411], LH (CGA LHB, P01215 P01229) [907, 1411]	TSH (CGA TSHB, P01215 P01222)
Labelled ligands	[^125^ I]FSH (human) (Agonist)	[^125^ I]LH (Agonist), [^125^ I]chorionic gonadotropin (human) (Agonist)	[^125^ I]TSH (human) (Agonist)

### Further reading on Glycoprotein hormone receptors

Jiang X et al. (2012) Structure of follicle‐stimulating hormone in complex with the entire ectodomain of its receptor. Proc. Natl. Acad. Sci. U.S.A. 109: 12491‐6 [PMID:22802634]

Kleinau G et al. TSH receptor mutations and disease. Accessed on 2017‐02‐23. Thyroid Disease Manager.

Tao YX et al. (2009) Follicle stimulating hormone receptor mutations and reproductive disorders. Prog Mol Biol Transl Sci 89: 115‐31 [PMID:20374735]

Troppmann B et al. (2013) Structural and functional plasticity of the luteinizing hormone/choriogonadotrophin receptor. Hum. Reprod. Update 19: 583‐602 [PMID:23686864]

## Gonadotrophin‐releasing hormone receptors

### Overview

GnRH_1_ and GnRH_2_ receptors (provisonal nomenclature [557], also called Type I and Type II GnRH receptor, respectively [1342]) have been cloned from numerous species, most of which express two or three types of GnRH receptor [1341, 1342, 1810]. GnRH I (GNRH1, P01148) (p‐Glu‐His‐Trp‐Ser‐Tyr‐Gly‐Leu‐Arg‐Pro‐Gly‐NH2) is a hypothalamic decapeptide also known as luteinizing hormone‐releasing hormone, gonadoliberin, luliberin, gonadorelin or simply as GnRH. It is a member of a family of similar peptides found in many species [1341, 1342, 1810] including GnRH II(GNRH2, O43555) (pGlu‐His‐Trp‐Ser‐His‐Gly‐Trp‐Tyr‐Pro‐Gly‐NH_2_(which is also known as chicken GnRH‐II). Receptors for three forms of GnRH exist in some species but only GnRH I and GnRH II and their cognate receptors have been found in mammals [1341, 1342, 1810]. GnRH_1_ receptors are expressed by pituitary gonadotrophs, where they mediate the effects of GnRH on gonadotropin hormone synthesis and secretion that underpin central control of mammalian reproduction. GnRH analogues are used in assisted reproduction and to treat steroid hormone‐dependent conditions [981]. Notably, agonists cause desensitization of GnRH‐stimulated gonadotropin secretion and the consequent reduction in circulating sex steroids is exploited to treat hormone‐dependent cancers of the breast, ovary and prostate [981]. GnRH1 receptors are selectively activated by GnRH I and all lack the COOH‐terminal tails found in other GPCRs. GnRH_2_ receptors do have COOH‐terminal tails and (where tested) are selective for GnRH II over GnRH I. GnRH2 receptors are expressed by some primates but not by humans [1377]. Phylogenetic classifications divide GnRH receptors into three [1342] or five groups [2117] and highlight examples of gene loss through evolution, with humans retaining only one ancient gene.

**Table:** 

Nomenclature	GnRH_1_ receptor	GnRH_2_ receptor
HGNC, UniProt	GNRHR, P30968	GNRHR2, Q96P88
Potency order of endogenous ligands	GnRH I (GNRH1, P01148) >GnRH II (GNRH2, O43555) [1342]	GnRH II (GNRH2, O43555) >GnRH I (GNRH1, P01148) (Monkey) [1340]
Endogenous agonists	GnRH I (GNRH1, P01148) [1214], GnRH II (GNRH2, O43555) [550, 1214, 1869]	GnRH II (GNRH2, O43555) [1340] – Monkey, GnRH I (GNRH1, P01148) [1340] – Monkey
Selective agonists	buserelin [1432], buserelin [1431], buserelin [1431], triptorelin [118], leuprolide [1881], goserelin, histrelin, nafarelin	–
Antagonists	iturelix (p*K*_i_ 9.5) [1664]	–
Selective antagonists	cetrorelix (p*K*_i_ 9.3–10) [119, 120, 1881], abarelix (p*K*_i_ 9.1–9.5) [1881], degarelix (p*K*_i_ 8.8) [2017], ganirelix	trptorelix‐1 [1246] – Monkey
Labelled ligands	[^125^ I]cetrorelix (Antagonist) (p*K*_d_ 9.7) [807], [^125^ I]triptorelin (Agonist) [435] – Rat, [^125^ I]buserelin (Agonist) [1076] – Rat, [^125^ I]GnRH I (human, mouse, rat) (Agonist)	–

### Comments

GnRH_1_ and GnRH_2_ receptors couple primarily to G_q/11_[686] but coupling to G_s_ and G_i_ is evident in some systems [1056, 1076]. GnRH_2_ receptors may also mediate (heterotrimeric) G protein‐independent signalling to protein kinases [289]. There is increasing evidence for expression of GnRH receptors on hormone‐dependent cancer cells where they can exert antiproliferative and/or proapoptotic effects and mediate effects of cytotoxins conjugated to GnRH analogues [324, 741, 1174, 1732]. In some human cancer cell models GnRH II(GNRH2, O43555) is more potent than GnRH I (GNRH1, P01148), implying mediation by GnRH_2_ receptors [690], but GnRH_2_ receptors are not expressed by humans because the human GNRHR2 gene contains a frame shift and internal stop codon [1377]. The possibility remains that this gene generates GnRH_2_ receptor'related proteins (other than the full‐length receptor) that mediate responses to GnRH II(GNRH2, O43555) (see [1436]). Alternatively, evidence for multiple active GnRH receptor conformations [289, 290, 541, 1293, 1342] raises the possibility that GnRH_1_ receptor‐mediated proliferation inhibition in hormone‐dependent cancer cells is dependent upon different conformations than effects on G_q/11_ in pituitary cells [290, 1293]. Loss‐of‐function mutations in the GnRH_1_ receptor and deficiency of GnRH I (GNRH1, P01148) are associated with hypogonadotropic hypogonadism although some 'loss of function' mutations may actually prevent trafficking of 'functional' GnRH_1_ receptors to the cell surface, as evidenced by recovery of function by nonpeptide antagonists [1124]. Human GnRH_1_ receptors are poorly expressed at the cell surface because of failure to meet structural quality control criteria for endoplasmic reticulum exit [542, 1124], and this increase susceptibility to point mutations that further impair trafficking [542, 1124]. GnRH receptor signalling may require receptor oligomerisation [376, 1054].

### Further reading on Gonadotrophin‐releasing hormone receptors

Limonta P et al. (2012) GnRH receptors in cancer: from cell biology to novel targeted therapeutic strategies. Endocr. Rev. 33: 784‐811 [PMID:22778172]

McArdle CA and Roberson MS.. (2015) Gonadotropes and gonadotropin‐releasing hormone signaling. In Knobil and Neill's Physiology of Reproduction (4th edition). Edited by Plant TM and Zeleznik AJ.: Elsevier Inc.: [ISBN: 9780123971753]

Millar RP et al. (2004) Gonadotropin‐releasing hormone receptors. Endocr Rev 25: 235‐275 [PMID:15082521]

Tao YX et al. (2014) Chaperoning G protein‐coupled receptors: from cell biology to therapeutics. Endocr. Rev. 35: 602‐47 [PMID:24661201]

## GPR18, GPR55 and GPR119

### Overview

GPR18, GPR55 and GPR119 (provisional nomenclature), although showing little structural similarity to CB_1_ and CB_2_ cannabinoid receptors, respond to endogenous agents analogous to the endogenous cannabinoid ligands, as well as some natural/synthetic cannabinoid receptor ligands [1564]. Although there are multiple reports to indicate that GPR18, GPR55 and GPR119 can be activated in vitro by N‐arachidonoylglycine, lysophosphatidylinositol and N‐oleoylethanolamide, respectively, there is a lack of evidence for activation by these lipid messengers in vivo. As such, therefore, these receptors retain their orphan status.

**Table:** 

Nomenclature	GPR18	GPR55	GPR119
HGNC, UniProt	GPR18, Q14330	GPR55, Q9Y2T6	GPR119, Q8TDV5
Potency order of endogenous ligands	–	–	N‐oleoylethanolamide, N‐palmitoylethanolamine>SEA (anandamide is ineffective) [1520]
Endogenous agonists	N‐arachidonoylglycine [1026]	lysophosphatidylinositol [773, 1502, 1854], 2‐arachidonoylglycerolphosphoinositol [1504]	N‐oleoylethanolamide [354, 1520, 1854], N‐palmitoylethanolamine, SEA
Selective agonists	–	AM251 [773, 948, 1695]	AS1269574 [2190], PSN632408 [1520], PSN375963 [1520]
Selective antagonists	–	CID16020046 (pA_2_ 7.3) [949]	–
Comments	The pairing of N‐arachidonoylglycine with GPR18 was not replicated in two studies based on arrestin assays [1854, 2182]. See [414] for discussion.	See reviews [414] and [1800].	In addition to those shown above, further small molecule agonists have been reported [722].

### Comments

GPR18 failed to respond to a variety of lipid‐derived agents in an in vitro screen [2182], but has been reported to be activated by Δ^9^ ‐tetrahydrocannabinol[1308]. GPR55 responds to AM251 and rimonabant at micromolar concentrations, compared to their nanomolar affinity as CB_1_ receptor antagonists/inverse agonists [1564]. It has been reported that lysophosphatidylinositol acts at other sites in addition to GPR55 [2164]. N‐Arachidonoylserine has been suggested to act as a low efficacy agonist/antagonist at GPR18 in vitro [1306]. It has also been suggested oleoyl‐lysophosphatidylcholine acts, at least in part, through GPR119 [1466]. Although PSN375963 and PSN632408 produce GPR119‐dependent responses in heterologous expression systems, comparison with N‐oleoylethanolamide‐mediated responses suggests additional mechanisms of action [1466].

### Further reading on GPR18, GPR55 and GPR119

Davenport AP et al. (2013) International Union of Basic and Clinical Pharmacology. LXXXVIII. G protein‐coupled receptor list: recommendations for new pairings with cognate ligands. Pharmacol. Rev. 65: 967‐86 [PMID:23686350]

Hassing HA et al. (2016) Biased signaling of lipids and allosteric actions of synthetic molecules for GPR119. Biochem Pharmacol 119: 66‐75 [PMID:27569424]

Liu B et al. (2015) GPR55: from orphan to metabolic regulator? Pharmacol Ther 145: 35‐42 [PMID:24972076]

Pertwee RG et al. (2010) International Union of Basic and Clinical Pharmacology. LXXIX. Cannabinoid receptors and their ligands: beyond CB_1 and CB_2. Pharmacol. Rev. 62: 588‐631 [PMID:21079038]

## Histamine receptors

### Overview

Histamine receptors (nomenclature as agreed by the NC‐IUPHAR Subcommittee on Histamine Receptors [790, 1528]) are activated by the endogenous ligand histamine. Marked species differences exist between histamine receptor orthologues [790]. The human and rat H_3_ receptor genes are subject to significant splice variance [91]. The potency order of histamine at histamine receptor subtypes is H_3_ = H_4_> H_2_> H_1_[1528]. Some agonists at the human H_3_ receptor display significant ligand bias [1659]. Antagonists of all 4 histamine receptors have clinical uses: H_1_ antagonists for allergies (e.g. cetirizine), H_2_ antagonists for acid‐reflux diseases (e.g. ranitidine), H_3_ antagonists for narcolepsy (e.g. pitolisant/WAKIX; Registered) and H_4_ antagonists for atopic dermatitis (e.g. ZPL‐3893787; Phase IIa) [1528].

**Table:** 

Nomenclature	H_1_ receptor	H_2_ receptor	H_3_ receptor	H_4_ receptor
HGNC, UniProt	HRH1, P35367	HRH2, P25021	HRH3, Q9Y5N1	HRH4, Q9H3N8
Selective agonists	methylhistaprodifen [1766], histaprodifen [1173]	amthamine [1048]	immethridine [1011], methimepip [1010], MK‐0249 (Inverse agonist) [1413]	clobenpropit (Partial agonist) [517, 1173, 1188, 1189, 1389], 4‐methylhistamine [617, 1173], ST‐1006 [1528], VUF 8430 [1172]
Antagonists	cyproheptadine (p*K*_i_ 10.2) [1352], promethazine (p*K*_i_ 9.6) [636], pyrilamine (Inverse agonist) (p*K*_i_ 8.7–9) [188, 1634], cetirizine (Inverse agonist) (p*K*_i_ 8.2) [1352], diphenhydramine (p*K*_i_ 7.9) [188]	–	iodophenpropit (p*K*_i_ 8.2–8.7) [2112, 2139]	–
Sub/family‐selective antagonists	–	–	thioperamide (Selective for H_3_/H_4_ compared to H_1_ and H_3_.) (p*K*_i_ 7.1–7.7) [368, 516, 517, 1170, 1211, 2112, 2139]	thioperamide (Selective for H_3_/H_4_ compared to H_1_ and H_3_.) (p*K*_i_ 6.3–7.6) [516, 517, 1188, 1189, 1389, 2226]
Selective antagonists	clemastine (p*K*_i_ 10.3) [71], desloratadine (p*K*_i_ 9) [1156], triprolidine (p*K*_i_ 8.5–9) [188, 1352], azelastine (p*K*_i_ 8.9) [1606], astemizole (p*K*_i_ 8.5) [1547]	tiotidine (p*K*_i_ 7.5) [149] – Rat, ranitidine (p*K*_i_ 7.1) [1152], cimetidine (p*K*_i_ 6.8) [274]	pitolisant (p*K*_i_ 8.1–8.6) [1528, 2254], A331440 (p*K*_i_ 8.5) [723], conessine (p*K*_i_ 8.3) [1528], MK‐0249 (p*K*_i_ 8.2) [1528], ciproxifan (p*K*_i_ 6.7–7.3) [368, 516, 517, 1170, 1528, 2139]	ZPL‐3893787 (p*K*_i_ 8.3) [1528], INCB‐38579 (p*K*_i_ 8.3) [1528], JNJ 7777120 (p*K*_i_ 7.8–8.3) [1173, 1839, 1959], JNJ‐39758979 (p*K*_i_ 7.9) [1528, 1727]
Labelled ligands	[^3^ H]pyrilamine (Antagonist, Inverse agonist) (p*K*_d_ 8.4–9.1) [422, 1352, 1746, 1766], [^11^ C]doxepin (Antagonist) (p*K*_d_ 9) [869], [^11^ C]pyrilamine (Antagonist, Inverse agonist)	[^125^ I]iodoaminopotentidine (Antagonist) (p*K*_d_ 8.7) [1082] – Rat, [^3^ H]tiotidine (Antagonist) (p*K*_d_ 7.7–8.7) [1363]	[^123^ I]iodoproxyfan (Antagonist) (p*K*_d_ 10.2) [1170], [^125^ I]iodophenpropit (Antagonist) (p*K*_d_ 9.2) [891] – Rat, [^3^ H](R)‐α‐methylhistamine (Agonist) [1188], N‐[^3^H]*α* ‐methylhistamine (Agonist) [317] – Mouse	[^3^ H]JNJ 7777120 (Antagonist) (p*K*_d_ 8.4) [1959]

### Comments

histaprodifen and methylhistaprodifen are reduced efficacy agonists. The H_4_ receptor appears to exhibit broadly similar pharmacology to the H_3_ receptor for imidazole‐containing ligands, although (R)‐*α* ‐methylhistamine and N‐*α* ‐methylhistamine are less potent, while clobenpropit acts as a reduced efficacy agonist at the H_4_ receptor and an antagonist at the H_3_ receptor [1188, 1419, 1455, 1489, 2226]. Moreover, 4‐methylhistamine is identified as a high affinity, full agonist for the human H_4_ receptor [1173]. [^3^ H]histamine has been used to label the H_4_ receptor in heterologous expression systems.

### Further reading on Histamine receptors

Gbahou F et al. (2012) The histamine autoreceptor is a short isoform of the H_3 receptor. Br. J. Pharmacol. 166: 1860‐71 [PMID:22356432]

Nieto‐Alamilla G et al. (2016) The Histamine H3 Receptor: Structure, Pharmacology, and Function. Mol. Pharmacol. 90: 649‐673 [PMID:27563055]

Panula P et al. (2015) International Union of Basic and Clinical Pharmacology. XCVIII. Histamine Receptors. Pharmacol. Rev. 67: 601‐55 [PMID:26084539]

van Rijn RM et al. (2008) Cloning and characterization of dominant negative splice variants of the human histamine H4 receptor. Biochem. J. 414: 121‐31 [PMID:18452403]

## Hydroxycarboxylic acid receptors

### Overview

The hydroxycarboxylic acid family of receptors (ENSFM00500000271913, nomenclature as agreed by the NC‐IUPHAR Subcommittee on Hydroxycarboxylic acid receptors [414, 1491]) respond to organic acids, including the endogenous hydroxy carboxylic acids 3‐hydroxy butyric acid and L‐lactic acid, as well as the lipid lowering agents nicotinic acid (niacin), acipimox and acifran[1842, 1989, 2125]. These receptors were provisionally described as nicotinic acid receptors, although nicotinic acid shows submicromolar potency at HCA_2_receptors only and is unlikely to be the natural ligand [1989, 2125].

**Table:** 

Nomenclature	HCA_1_ receptor	HCA_2_ receptor	HCA_3_ receptor
HGNC, UniProt	HCAR1, Q9BXC0	HCAR2, Q8TDS4	HCAR3, P49019
Potency order of endogenous ligands	–	*β* ‐D‐hydroxybutyric acid>butyric acid	–
Endogenous agonists	L‐lactic acid [16, 266, 1190, 1854]	*β* ‐D‐hydroxybutyric acid [1912], butyric acid	3‐hydroxyoctanoic acid [15]
Agonists	3,5‐dihydroxybenzoic acid [1187]	SCH 900271 [1522], GSK256073 [1861]	–
Selective agonists	–	MK 6892 [1788], MK 1903 [166], nicotinic acid [1842, 1989, 2125], acipimox [1842, 2125], monomethylfumarate [1933]	compound 6o [1820], IBC 293 [1769]
Labelled ligands	–	[^3^ H]nicotinic acid (Agonist) [1842, 1989, 2125]	–

### Comments

Further closely‐related GPCRs include the 5‐oxoeicosanoid receptor (OXER1, Q8TDS5) and GPR31(O00270). Lactate activates HCA_1_ on adipocytes in an autocrine manner. It inhibits lipolysis and thereby promotes anabolic effects. HCA_2_ and HCA_3_ regulate adipocyte lipolysis and immune functions under conditions of increased FFA formation through lipolysis (e.g., during fasting). HCA_2_ agonists acting mainly through the receptor on immune cells exert antiatherogenic and anti‐inflammatory effects. HCA_2_ is also a receptor for butyrate and mediates some of the beneficial effects of short‐chain fatty acids produced by gut microbiota.

### Further reading on Hydroxycarboxylic acid receptors

Boatman PD et al. (2008) Nicotinic acid receptor agonists. J. Med. Chem. 51: 7653‐62 [PMID:18983141]

Graff EC et al. (2016) Anti‐inflammatory effects of the hydroxycarboxylic acid receptor 2. Metab. Clin. Exp. 65: 102‐13 [PMID:26773933]

Kamanna VS et al. (2013) Recent advances in niacin and lipid metabolism. Curr. Opin. Lipidol. 24: 239‐45 [PMID:23619367]

Offermanns S. (2017) Hydroxy‐Carboxylic Acid Receptor Actions in Metabolism. Trends Endocrinol. Metab. [PMID:28087125]

Offermanns S et al. (2011) International Union of Basic and Clinical Pharmacology. LXXXII: Nomenclature and Classification of Hydroxy‐carboxylic Acid Receptors (GPR81, GPR109A, and GPR109B). Pharmacol. Rev. 63: 269‐90 [PMID:21454438]

Offermanns S et al. (2015) Nutritional or pharmacological activation of HCA(2) ameliorates neuroinflammation. Trends Mol Med 21: 245‐55 [PMID:25766751]

## Kisspeptin receptor

### Overview

The kisspeptin receptor (nomenclature as agreed by the NC‐IUPHAR Subcommittee on the kisspeptin receptor [1004]), like neuropeptide FF (NPFF), prolactin‐releasing peptide (PrP) and QRFP receptors (provisional nomenclature) responds to endogenous peptides with an arginine‐phenylalanine‐amide (RFamide) motif. Kisspeptin‐54(KISS1, Q15726) (KP54, originally named metastin), kisspeptin‐13(KISS1, Q15726) (KP13) and kisspeptin‐10(KISS1) (KP10) are biologically‐active peptides cleaved from the KISS1(Q15726) gene product. Kisspeptins have roles in, for example, cancer metastasis, fertility/puberty regulation and glucose homeostasis.

**Table:** 

Nomenclature	kisspeptin receptor
HGNC, UniProt	KISS1R, Q969F8
Endogenous agonists	kisspeptin‐10 (KISS1) [1041, 1501], kisspeptin‐54 (KISS1, Q15726) [1041, 1501], kisspeptin‐14 (KISS1, Q15726) [1041], kisspeptin‐13 (KISS1, Q15726) [1041]
Selective agonists	4‐fluorobenzoyl‐FGLRW‐NH2 [1973], [dY]^1^ KP‐10 [401] – Mouse
Selective antagonists	peptide 234 [1674]
Labelled ligands	[^125^I]Tyr^45^ ‐kisspeptin‐15 (Agonist) [1501], [^125^ I]kisspeptin‐13 (human) (Agonist) [1314], [^125^ I]kisspeptin‐10 (human) (Agonist) [1041], [^125^ I]kisspeptin‐14 (human) (Agonist) [1314]

### Further reading on Kisspeptin receptor

Kanda S et al. (2013) Structure, synthesis, and phylogeny of kisspeptin and its receptor. Adv. Exp. Med. Biol. 784: 9‐26 [PMID:23550000]

Kirby HR et al. (2010) International Union of Basic and Clinical Pharmacology. LXXVII. Kisspeptin receptor nomenclature, distribution, and function. Pharmacol. Rev. 62: 565‐78 [PMID:21079036]

Millar RP et al. (2010) Kisspeptin antagonists: unraveling the role of kisspeptin in reproductive physiology. Brain Res. 1364: 81‐9 [PMID:20858467]

Oakley AE et al. (2009) Kisspeptin signaling in the brain. Endocr. Rev. 30: 713‐43 [PMID:19770291]

Pasquier J et al. (2014) Molecular evolution of GPCRs: Kisspeptin/kisspeptin receptors. J. Mol. Endocrinol. 52: T101‐17 [PMID:24577719]

## Leukotriene receptors

### Overview

The leukotriene receptors (nomenclature as agreed by the NC‐IUPHAR subcommittee on Leukotriene Receptors [257, 258]) are activated by the endogenous ligands leukotrienes (LT), synthesized from lipoxygenase metabolism of arachidonic acid. The human BLT_1_ receptor is the high affinity LTB_4_ receptor whereas the BLT_2_ receptor in addition to being a low‐affinity LTB_4_ receptor also binds several other lipoxygenase‐products, such as 12S‐HETE, 12S‐HPETE, 15S‐HETE, and the thromboxane synthase product 12‐hydroxyheptadecatrienoic acid. The BLT receptors mediate chemotaxis and immunomodulation in several leukocyte populations and are in addition expressed on non‐myeloid cells, such as vascular smooth muscle and endothelial cells. In addition to BLT receptors, LTB_4_ has been reported to bind to the peroxisome proliferator activated receptor (PPAR) *α*[1178] and the vanilloid TRPV1 ligand‐gated nonselective cation channel [1307]. The receptors for the cysteinyl‐leukotrienes (i.e. LTC_4_, LTD_4_ and LTE_4_) are termed CysLT_1_ and CysLT_2_ and exhibit distinct expression patterns in human tissues, mediating for example smooth muscle cell contraction, regulation of vascular permeability, and leukocyte activation. There is also evidence in the literature for additional CysLT receptor subtypes, derived from functional in vitro studies, radioligand binding and in mice lacking both CysLT_1_ and CysLT_2_ receptors [258]. Cysteinyl‐leukotrienes have also been suggested to signal through the P2Y_12_ receptor [570, 1473, 1534], GPR17 [359] and GPR99 [943].

**Table:** 

Nomenclature	BLT_1_ receptor	BLT_2_ receptor	CysLT_1_ receptor	CysLT_2_ receptor
HGNC, UniProt	LTB4R, Q15722	LTB4R2, Q9NPC1	CYSLTR1, Q9Y271	CYSLTR2, Q9NS75
Potency order of endogenous ligands	LTB_4_>20‐hydroxy‐LTB_4_≫12R‐HETE [2185]	12‐hydroxyheptadecatrienoic acid>LTB_4_>12S‐HETE = 12S‐HPETE>15S‐HETE>12R‐HETE>20‐hydroxy‐LTB_4_ [1510, 2185]	LTD_4_>LTC_4_>LTE_4_ [1222, 1716]	LTC_4_≥LTD_4_≫LTE_4_ [767, 1477, 1920]
Endogenous agonists	–	12S‐HETE (Partial agonist) [2185]	–	–
Antagonists	–	–	ICI198615 (p*K*_i_ 9.7) [591] – Guinea pig	BAYu9773 (pA_2_ 6.8–7.7) [1988] – Rat
Selective antagonists	BIIL 260 (p*K*_i_ 8.8) [156, 485], CP105696 (pIC_50_ 8.1) [1803], U75302 (p*K*_i_ 6.4) [174]	LY255283 (pIC_50_ 6–7.1) [780, 2185]	zafirlukast (pIC_50_ 8.6–8.7) [1222, 1716], SR2640 (p*K*_i_ 8.7), montelukast (pIC_50_ 8.3–8.6) [1222, 1716], sulukast (p*K*_i_ 8.3), pobilukast (pIC_50_ 7.5) [1716]	BayCysLT_2_ (pIC_50_ 6.6–7.3) [1456]
Labelled ligands	[^3^ H]LTB_4_ (Agonist) [2184], [^3^ H]CGS23131 (Antagonist) (p*K*_d_ 7.9) [877]	[^3^ H]LTB_4_ (p*K*_d_ 7.6–9.7)	[^3^ H]LTD_4_ (Agonist), [^3^ H]ICI‐198615 (Antagonist) (p*K*_d_ 10.6) [1682]	[^3^ H]LTD_4_ (Agonist) [767]

**Table:** 

Nomenclature	OXE receptor	FPR2/ALX
HGNC, UniProt	OXER1, Q8TDS5	FPR2, P25090
Potency order of endogenous ligands	5‐oxo‐ETE, 5‐oxo‐C20:3, 5‐oxo‐ODE>5‐oxo‐15‐HETE>5S‐HPETE>5S‐HETE [823, 920, 1538]	LXA_4_ = aspirin triggered lipoxin A4 = ATLa2 = resolvin D1>LTC_4_ = LTD_4_≫15‐deoxy‐LXA4≫fMet‐Leu‐Phe [365, 544, 546, 684, 1919]
Endogenous agonists	5‐oxo‐ETE [672, 1483, 1538, 1597, 1752]	LXA_4_ [1052], resolvin D1 [1052], aspirin‐triggered resolvin D1 [1051], aspirin triggered lipoxin A4
Selective agonists	–	ATLa2 [697]
Endogenous antagonists	5‐oxo‐12‐HETE (pIC_50_ 6.3) [1596]	–
Selective antagonists	–	WRWWWW (pIC_50_ 6.6) [83], t‐Boc‐FLFLF (pIC_50_ 4.3–6) [574, 1867, 2061]
Labelled ligands	[^3^ H]5‐oxo‐ETE (Agonist) [1483]	[^3^ H]LXA_4_ (Agonist) [544, 545]

### Comments

The FPR2/ALX receptor (nomenclature as agreed by the NC‐IUPHAR subcommittee on Leukotriene and Lipoxin Receptors [258]) is activated by the endogenous lipid‐derived, anti‐inflammatory ligands lipoxin A_4_(LXA_4_) and 15‐epi‐LXA_4_(aspirin triggered lipoxin A4, ATL). The FPR2/ALX receptor also interacts with endogenous peptide and protein ligands, such as MHC binding peptide [330] as well as annexin I (ANXA1, P04083) (ANXA1) and its N‐terminal peptides [379, 1560]. In addition, a soluble hydrolytic product of protease action on the urokinase‐type plasminogen activator receptor has been reported to activate the FPR2/ALX receptor [1646]. Furthermore, FPR2/ALX has been suggested to act as a receptor mediating the proinflammatory actions of the acute‐phase reactant, serum amyloid A [1840, 1883]. The agonist activity of the lipid mediators described has been questioned [732, 1585], which may derive from batch‐to‐batch differences, partial agonism or biased agonism. Recent results from Cooray et al. (2013) [379] have addressed this issue and the role of homodimers and heterodimers in intracellular signaling. A receptor selective for LXB_4_ has been suggested from functional studies [58, 1232, 1670]. Note that the data for FPR2/ALX are also reproduced on the Formylpeptide receptor pages.

Oxoeicosanoid receptors (OXE, nomenclature agreed by the NC‐IUPHAR subcommittee on Oxoeicosanoid Receptors [219]) are activated by endogenous chemotactic eicosanoid ligands oxidised at the C‐5 position, with 5‐oxo‐ETE the most potent agonist identified for this receptor. Initial characterization of the heterologously expressed OXE receptor suggested that polyunsaturated fatty acids, such as docosahexaenoic acid and EPA, acted as receptor antagonists [823].

### Further reading on Leukotriene receptors

Bäck M et al. (2011) International Union of Basic and Clinical Pharmacology. LXXXIV: leukotriene receptor nomenclature, distribution, and pathophysiological functions. Pharmacol. Rev. 63: 539‐84 [PMID:21771892]

Bäck M et al. (2014) Update on leukotriene, lipoxin and oxoeicosanoid receptors: IUPHAR Review 7. Br. J. Pharmacol. 171: 3551‐74 [PMID:24588652]

Brink C et al. (2004) International Union of Pharmacology XLIV. Nomenclature for the Oxoeicosanoid Receptor. Pharmacol. Rev. 56: 149‐157 [PMID:15001665]

Brink C et al. (2003) International Union of Pharmacology XXXVII. Nomenclature for leukotriene and lipoxin receptors. Pharmacol. Rev. 55: 195‐227 [PMID:12615958]

## Lysophospholipid (LPA) receptors

### Overview

Lysophosphatidic acid (LPA) receptors (nomenclature as agreed by the NC‐IUPHAR Subcommittee on Lysophospholipid Receptors [414, 983]) are activated by the endogenous phospholipid metabolite LPA. The first receptor, LPA_1_, was identified as ventricular zone gene‐1 (vzg‐1), leading to deorphanisation of members of the endothelial differentiation gene (edg) family as other LPA receptors along with sphingosine 1‐phosphate (S1P) receptors. Additional LPA receptor GPCRs were later identified. Gene names have been codified as LPAR1, etc. to reflect the receptor function of proteins. The crystal structure of LPA_1_ was recently solved and demonstrates extracellular LPA access to the binding pocket, consistent with proposed delivery via autotaxin. These studies have also implicated cross‐talk with endocannabinoids via phosphorylated intermediates that can also activate these receptors. The identified receptors can account for most, although not all, LPA‐induced phenomena in the literature, indicating that a majority of LPA‐dependent phenomena are receptor‐mediated. Radioligand binding has been conducted in heterologous expression systems using [^3^ H]LPA(e.g. [586]). In native systems, analysis of binding data is complicated by metabolism and high levels of nonspecific binding, and therefore the relationship between recombinant and endogenously expressed receptors is unclear. Targeted deletion of LPA receptors has clarified signalling pathways and identified physiological and pathophysiological roles. Independent validation by multiple groups has been reported in the peer‐reviewed literature for all six LPA receptors described in the tables, including further validation using a distinct read‐out via a novel TGF*α*"shedding" assay [864]. LPA has also been described as an agonist for the transient receptor potential (Trp) ion channel TRPV1 [1461] and TRPA1 [1012]. In addition, orphan GPCRs (PSP24 [547] and GPR87 [1488]) are proposed as LPA receptors. LPA was originally proposed to be a ligand for GPCR35, but recent data shows that in fact it is a receptor for CXCL17(CXCL17, Q6UXB2) [1266]. Further, the nuclear hormone receptor PPAR*γ*[1309, 1812], has been reported as an LPA receptor. All of these proposed entities require confirmation and are not currently recognized as bona fide LPA receptors.

**Table:** 

Nomenclature	LPA_1_ receptor	LPA_2_ receptor	LPA_3_ receptor	LPA_4_ receptor	LPA_5_ receptor	LPA_6_ receptor
HGNC, UniProt	LPAR1, Q92633	LPAR2, Q9HBW0	LPAR3, Q9UBY5	LPAR4, Q99677	LPAR5, Q9H1C0	LPAR6, P43657
Selective agonists	–	dodecylphosphate [2038], decyl dihydrogen phosphate [2038], GRI977143 [1007]	OMPT [744]	–	–	–
Sub/family‐selective antagonists	Ki16425 (pIC_50_ 6.6–6.9) [1499] – Mouse, VPC12249 (p*K*_i_ 5.2–6.9) [769] – Mouse, VPC32179 [763]	–	Ki16425 (p*K*_i_ 6.4) [1499], VPC12249 (p*K*_i_ 6.4) [769], VPC32179 [763]	–	–	–
Selective antagonists	BMS‐986020 (pIC_50_ 8.9), AM966 (pIC_50_ 6.7–7.8) [1905], ONO‐7300243 (pIC_50_ 6.8) [1940], AM095 (pIC_50_ 6–6.1) [1905]	–	dioctanoylglycerol pyrophosphate (p*K*_i_ 5.5–7) [548, 1499]	–	TCLPA5 (pIC_50_ 6.1) [1047]	–

### Comments

Ki16425[1499], VPC12249[769] and VPC32179[763] have dual antagonist activity at LPA_1_ and LPA_3_ receptors. There is growing evidence for in vivo efficacy of these chemical antagonists in several disorders, including fetal hydrocephalus [2197], fetal hypoxia [778], lung fibrosis [1495], systemic sclerosis [1495] and atherosclerosis progression [1053]. Virtual screening experiments have shown H2L5186303 to be a potent antagonist of LPA_2_[536]. Dodecylphosphate is also an antagonist at LPA_3_ receptors [2038].

### Further reading on Lysophospholipid (LPA) receptors

Chun J et al. (2010) International Union of Basic and Clinical Pharmacology. LXXVIII. Lysophospholipid receptor nomenclature. Pharmacol. Rev. 62: 579‐87 [PMID:21079037]

Kihara Y et al. (2014) Lysophospholipid receptor nomenclature review: IUPHAR Review 8. Br. J. Pharmacol. 171: 3575‐94 [PMID:24602016]

Yung YC et al. (2014) LPA receptor signaling: pharmacology, physiology, and pathophysiology. J. Lipid Res. 55: 1192‐1214 [PMID:24643338]

Yung YC et al. (2015) Lysophosphatidic Acid signaling in the nervous system. Neuron 85: 669‐82 [PMID:25695267]

## Lysophospholipid (S1P) receptors

### Overview

Sphingosine 1‐phosphate (S1P) receptors (nomenclature as agreed by the NC‐IUPHAR Subcommittee on Lysophospholipid receptors [983]) are activated by the endogenous lipid sphingosine 1‐phosphate (S1P) and with lower apparent affinity, sphingosylphosphorylcholine(SPC). Originally cloned as orphan members of the endothelial differentiation gene (edg) family, deorphanisation as lysophospholipid receptors for S1P was based on sequence homology to LPA receptors. Current gene names have been codified as S1P_1_R, etc. to reflect the receptor function of these proteins. Most cellular phenomena ascribed to S1P can be explained by receptor‐mediated mechanisms; S1P has also been described to act at intracellular sites [1915], and awaits precise definition. Previously‐proposed SPC (or lysophophosphatidylcholine) receptors‐ G2A, TDAG8, OGR1 and GPR4‐ continue to lack confirmation of these roles [414]. The relationship between recombinant and endogenously expressed receptors is unclear. Radioligand binding has been conducted in heterologous expression systems using [^32^ P]S1P(e.g [1505]). In native systems, analysis of binding data is complicated by metabolism and high levels of nonspecific binding. Targeted deletion of several S1P receptors and key enzymes involved in S1P biosynthesis or degradation has clarified signalling pathways and physiological roles. A crystal structure of an S1P_1_‐T4 fusion protein has been described [733].

The S1P receptor modulator, fingolimod(FTY720, Gilenya), has received world‐wide approval as the first oral therapy for relapsing forms of multiple sclerosis. This drug has a novel mechanism of action involving modulation of S1P receptors in both the immune and nervous systems [340, 369, 687], although the precise nature of its interaction requires clarification.

**Table:** 

Nomenclature	S1P_1_ receptor	S1P_2_ receptor	S1P_3_ receptor	S1P_4_ receptor	S1P_5_ receptor
HGNC, UniProt	S1PR1, P21453	S1PR2, O95136	S1PR3, Q99500	S1PR4, O95977	S1PR5, Q9H228
Potency order of endogenous ligands	sphingosine 1‐phosphate>dihydrosphingosine 1‐phosphate>sphingosylphosphorylcholine [46, 1505]	sphingosine 1‐phosphate>dihydrosphingosine 1‐phosphate>sphingosylphosphorylcholine [46, 1505]	sphingosine 1‐phosphate>dihydrosphingosine 1‐phosphate>sphingosylphosphorylcholine [1505]	sphingosine 1‐phosphate>dihydrosphingosine 1‐phosphate>sphingosylphosphorylcholine [2012]	sphingosine 1‐phosphate>dihydrosphingosine 1‐phosphate>sphingosylphosphorylcholine [861]
Agonists	amiselimod phosphate [1887], FTY720‐phosphate [220, 560, 1525], siponimod [1524], AUY954 [1525], AFD(R) [220], etrasimod [254], fingolimod [708]	–	–	–	–
Selective agonists	ozanimod [657, 1274, 1757], ponesimod [176], KRP 203‐phosphate [1851] – Mouse, CYM5181 [657], SEW2871 [1713] – Mouse	–	–	–	–
Antagonists	VPC23019 (p*K*_i_ 7.9) [419], VPC03090‐P (p*K*_i_ 7.6–7.7) [972], VPC44116 (pIC_50_ 7.6) [561]	–	VPC44116 (p*K*_i_ 6.5) [561], VPC23019 (p*K*_i_ 5.9) [419]	–	–
Selective antagonists	NIBR‐0213 (pIC_50_ 8.6) [1615], W146 (p*K*_i_ 7.1) [1714]	JTE‐013 (pIC_50_ 7.8) [1515]	–	–	–

### Comments

The FDA‐approved immunomodulator fingolimod(FTY720) can be phosphorylated in vivo [31] to generate a relatively potent agonist with activity at S1P_1_, S1P_3_, S1P_4_ and S1P_5_ receptors [220, 1259]. The physiological consequences of FTY720‐phosphate administration, as well as those of other S1P_1_ agonists, may involve functional antagonism via ubiquitination and subsequent degradation of S1P_1_[1514].

### Further reading on Lysophospholipid (S1P) receptors

Chew WS et al. (2016) To fingolimod and beyond: The rich pipeline of drug candidates that target S1P signaling. Pharmacol. Res. 113: 521‐532 [PMID:27663260]

Chun J et al. (2010) International Union of Basic and Clinical Pharmacology. LXXVIII. Lysophospholipid receptor nomenclature. Pharmacol. Rev. 62: 579‐87 [PMID:21079037]

Pyne NJ et al. (2017) Sphingosine 1‐Phosphate Receptor 1 Signaling in Mammalian Cells. Molecules 22: [PMID:28241498]

Rosen H et al. (2013) Sphingosine‐1‐phosphate and its receptors: structure, signaling, and influence. Annu. Rev. Biochem. 82: 637‐62 [PMID:23527695]

## Melanin‐concentrating hormone receptors

### Overview

Melanin‐concentrating hormone (MCH) receptors (provisional nomenclature as recommended by NC‐IUPHAR [557]) are activated by an endogenous nonadecameric cyclic peptide identical in humans and rats (DFDMLRCMLGRVYRPCWQV) generated from a precursor (PMCH, P20382), which also produces neuropeptide EI(PMCH, P20382) and neuropeptide GE (PMCH, P20382).

**Table:** 

Nomenclature	MCH_1_ receptor	MCH_2_ receptor
HGNC, UniProt	MCHR1, Q99705	MCHR2, Q969V1
Selective antagonists	GW803430 (pIC_50_ 9.3) [781], SNAP‐7941 (pA_2_ 9.2) [190], T‐226296 (pIC_50_ 8.3) [1926], ATC0175 (pIC_50_ 7.9–8.1) [297]	–
Labelled ligands	[^125^ I]S36057 (Antagonist) (p*K*_d_ 9.2–9.5) [69], [^125^I][Phe^13^,Tyr^19^ ]MCH (Agonist) [249], [^3^ H]MCH (human, mouse, rat) (Agonist) [249]	–

### Comments

The MCH_2_ receptor appears to be a non‐functional pseudogene in rodents [1930].

### Further reading on Melanin‐concentrating hormone receptors

Chung S et al. (2011) Recent updates on the melanin‐concentrating hormone (MCH) and its receptor system: lessons from MCH1R antagonists. J. Mol. Neurosci. 43: 115‐21 [PMID:20582487]

Eberle AN et al. (2010) Cellular models for the study of the pharmacology and signaling of melanin‐concentrating hormone receptors. J. Recept. Signal Transduct. Res. 30: 385‐402 [PMID:21083507]

Foord SM et al. (2005) International Union of Pharmacology. XLVI. G protein‐coupled receptor list. Pharmacol Rev 57: 279‐288 [PMID:15914470]

Takase K et al. (2014) Meta‐analysis of melanin‐concentrating hormone signaling‐deficient mice on behavioral and metabolic phenotypes. PLoS ONE 9: e99961 [PMID:24924345]

## Melanocortin receptors

### Overview

Melanocortin receptors (provisional nomenclature as recommended by NC‐IUPHAR[557]) are activated by members of the melanocortin family (*α* ‐MSH(POMC, P01189), *β* ‐MSH(POMC, P01189) and *γ* ‐MSH(POMC, P01189) forms; *δ* form is not found in mammals) and adrenocorticotrophin (ACTH(POMC, P01189)). Endogenous antagonists include agouti(ASIP, P42127) and agouti‐related protein (AGRP, O00253). ACTH(1‐24) was approved by the US FDA as a diagnostic agent for adrenal function test. At least 2 synthetic melanocortin receptor agonists are under clinical development as of 2017.

**Table:** 

Nomenclature	MC_1_ receptor	MC_2_ receptor	MC_3_ receptor	MC_4_ receptor	MC_5_ receptor
HGNC, UniProt	MC1R, Q01726	MC2R, Q01718	MC3R, P41968	MC4R, P32245	MC5R, P33032
Potency order of endogenous ligands	*α* ‐MSH (POMC, P01189) >*β* ‐MSH (POMC, P01189) >ACTH (POMC, P01189), *γ* ‐MSH (POMC, P01189)	ACTH (POMC, P01189)	*γ* ‐MSH (POMC, P01189), *β* ‐MSH (POMC, P01189) >ACTH (POMC, P01189), *α* ‐MSH (POMC, P01189)	*β* ‐MSH (POMC, P01189) >*α* ‐MSH (POMC, P01189), ACTH (POMC, P01189) >*γ* ‐MSH (POMC, P01189)	*α* ‐MSH (POMC, P01189) >*β* ‐MSH (POMC, P01189) >ACTH (POMC, P01189) >*γ* ‐MSH (POMC, P01189)
Selective agonists	–	corticotropin zinc hydroxide	[D‐Trp ^8^]*γ*‐MSH [679]	THIQ [1760]	–
Antagonists	–	–	PG‐106 (pIC_50_ 6.7) [680]	–	–
Selective antagonists	–	–	–	MBP10 (pIC_50_ 10) [123], HS014 (p*K*_i_ 8.5) [1738]	–
Labelled ligands	[^125^ I]NDP‐MSH (Agonist) [1037]	[^125^ I]ACTH‐(1‐24) (Agonist)	[^125^ I]NDP‐MSH (Agonist) [1037], [^125^ I]SHU9119 (Antagonist) [1457]	[^125^ I]SHU9119 (Antagonist) (p*K*_d_ 9.2) [1457], [^125^ I]NDP‐MSH (Agonist) [1037, 1736]	[^125^ I]NDP‐MSH (Agonist) [1037]

### Comments

Polymorphisms of the MC_1_ receptor have been linked to variations in skin pigmentation. Defects of the MC_2_ receptor underlie familial glucocorticoid deficiency. Polymorphisms of the MC_4_ receptor have been linked to obesity [296, 531].

### Further reading on Melanocortin receptors

Caruso V et al. (2014) Synaptic changes induced by melanocortin signalling. Nat. Rev. Neurosci. 15: 98‐110 [PMID:24588018]

Foord SM et al. (2005) International Union of Pharmacology. XLVI. G protein‐coupled receptor list. Pharmacol Rev 57: 279‐288 [PMID:15914470]

Renquist BJ et al. (2011) Physiological roles of the melanocortin MC_3 receptor. Eur. J. Pharmacol. 660: 13‐20 [PMID:21211527]

## Melatonin receptors

### Overview

Melatonin receptors (nomenclature as agreed by the NC‐IUPHARSubcommittee on Melatonin Receptors [474]) are activated by the endogenous ligands melatonin and clinically used drugs like ramelteon and agomelatine.

**Table:** 

Nomenclature	MT_1_ receptor	MT_2_ receptor
HGNC, UniProt	MTNR1A, P48039	MTNR1B, P49286
Endogenous agonists	melatonin [70, 473, 475]	melatonin [70, 473, 475]
Agonists	ramelteon [954], agomelatine [70, 136]	agomelatine [70, 136], ramelteon [954, 1636]
Selective agonists	–	UCM1014 [1855], IIK7 [532, 1888], 5‐methoxy‐luzindole (Partial agonist) [475]
Selective antagonists	–	4P‐PDOT (p*K*_i_ 8.8–9.4) [70, 475, 476], K185 (p*K*_i_ 9.3) [532, 1888], DH97 (p*K*_i_ 8) [1939]
Labelled ligands	[^125^ I]SD6 (Agonist) [1138], 2‐[^125^ I]melatonin (Agonist) [70, 475], [^3^ H]melatonin (Agonist) [235]	[^125^ I]SD6 (Agonist) [1138], 2‐[^125^ I]melatonin (Agonist) [70, 475], [^125^ I]DIV880 (Agonist, Partial agonist) [1138], [^3^ H]melatonin (Agonist) [235]

### Comments

melatonin, 2‐iodo‐melatonin, agomelatine, GR 196429, LY 156735 and ramelteon[954] are nonselective agonists for MT_1_ and MT_2_ receptors. (‐)‐AMMTC displays an ~400‐fold greater agonist potency than (+)‐AMMTC at rat MT_1_ receptors (see AMMTC for structure) [1966]. Luzindole is an MT_1_/MT_2_ non‐selective competitive melatonin receptor antagonist with about 15‐25 fold selectivity for the MT_2_ receptor [476]. MT_1_/MT_2_ heterodimers present different pharmacological profiles from MT_1_ and MT_2_ receptors [75].

The MT_3_ binding site of hamster brain and peripheral tissues such as kidney and testis, also termed the ML_2_ receptor, binds selectively 2‐iodo‐[^125^ I]5MCA‐NAT[1356]. Pharmacological investigations of MT_3_ binding sites have primarily been conducted in hamster tissues. At this site, The endogenous ligand N‐acetylserotonin[495, 1215, 1356, 1588] and 5MCA‐NAT[1588] appear to function as agonists, while prazosin[1215] functions as an antagonist. The MT_3_ binding site of hamster kidney was also identified as the hamster homologue of human quinone reductase 2 (NQO2, P16083[1474, 1475]). The MT_3_ binding site activated by 5MCA‐NAT in eye ciliary body is positively coupled to adenylyl cyclase and regulates chloride secretion [842]. Xenopus melanophores and chick brain express a distinct receptor (x420, P49219; c346, P49288, initially termed Mel_1C_) coupled to the G_i/o_ family of G proteins, for which GPR50 has recently been suggested to be a mammalian counterpart [479] although melatonin does not bind to GPR50 receptors. Several variants of the MTNR1B gene have been associated with increased type 2 diabetes risk.

### Further reading on Melatonin receptors

Dubocovich ML et al. (2010) International Union of Basic and Clinical Pharmacology. LXXV. Nomenclature, classification, and pharmacology of G protein‐coupled melatonin receptors. Pharmacol. Rev. 62: 343‐80 [PMID:20605968]

Jockers R et al. (2016) Update on melatonin receptors: IUPHAR Review 20. Br. J. Pharmacol. 173: 2702‐25 [PMID:27314810]

Liu J et al. (2016) MT1 and MT2 Melatonin Receptors: A Therapeutic Perspective. Annu. Rev. Pharmacol. Toxicol. 56: 361‐83 [PMID:26514204]

Zlotos DP et al. (2013) MT1 and MT2 Melatonin Receptors: Ligands, Models, Oligomers, and Therapeutic Potential. J. Med. Chem. [PMID:24228714]

## Metabotropic glutamate receptors

### Overview

Metabotropic glutamate (mGlu) receptors (nomenclature as agreed by the NC‐IUPHAR Subcommittee on Metabotropic Glutamate Receptors [1743]) are a family of G‐protein coupled receptors activated by the neurotransmitter glutamate. The mGlu family is composed of eight members (named mGlu1 to mGlu8) which are divided in three groups based on similarities of agonist pharmacology, primary sequence and G protein coupling to effector: Group‐I (mGlu_1_ and mGlu_5_), Group‐II (mGlu_2_ and mGlu_3_) and Group‐III (mGlu_4_, mGlu_6_, mGlu_7_ and mGlu_8_) (see Further reading).

Structurally, mGlu are composed of three juxtaposed domains: a core G‐protein‐activating seven‐transmembrane domain (TM), common to all GPCRs, is linked via a rigid cysteine‐rich domain (CRD) to the Venus Flytrap domain (VFTD), a large bi‐lobed extracellular domain where glutamate binds. The structures of the VFTD of mGlu_1_, mGlu_2_, mGlu_3_, mGlu_5_ and mGlu_7_ have been solved [1075, 1364, 1408, 1984]. The structure of the 7 transmembrane (TM) domains of both mGlu1 and mGlu5 have been solved, and confirm a general helical organization similar to that of other GPCRs, although the helices appear more compacted [465, 2136]. mGlu form constitutive dimers crosslinked by a disulfide bridge. Although mGlu receptors have been thought to only form homodimers, recent studies revealed the possible formation of heterodimers between either group‐I receptors, or within and between group‐II and ‐III receptors [468]. Although well characterized in transfected cells, co‐localization and specific pharmacological properties also suggest the existence of such heterodimers in the brain [2183].

The endogenous ligands of mGlu are L‐glutamic acid, L‐serine‐O‐phosphate, N‐acetylaspartylglutamate (NAAG) and L‐cysteine sulphinic acid. Group‐I mGlu receptors may be activated by 3,5‐DHPG and (S)‐3HPG[204] and antagonized by (S)‐hexylhomoibotenic acid[1235]. Group‐II mGlu receptors may be activated by LY389795[1365], LY379268[1365], eglumegad[1744, 2138], DCG‐IV and (2R,3R)‐APDC[1745], and antagonised by eGlu[890] and LY307452[518, 2096]. Group‐III mGlu receptors may be activated by L‐AP4 and (R,S)‐4‐PPG[610]. An example of an antagonist selective for mGlu receptors is LY341495, which blocks mGlu_2_ and mGlu_3_ at low nanomolar concentrations, mGlu_8_ at high nanomolar concentrations, and mGlu_4_, mGlu_5_, and mGlu_7_ in the micromolar range [1001]. In addition to orthosteric ligands that directly interact with the glutamate recognition site, allosteric modulators that bind within the TM domain have been described. Negative allosteric modulators are listed separately. The positive allosteric modulators most often act as ‘potentiators’ of an orthosteric agonist response, without significantly activating the receptor in the absence of agonist.

**Table:** 

Nomenclature	mGlu_1_ receptor	mGlu_2_ receptor	mGlu_3_ receptor	mGlu_4_ receptor	mGlu_5_ receptor
HGNC, UniProt	GRM1, Q13255	GRM2, Q14416	GRM3, Q14832	GRM4, Q14833	GRM5, P41594
Endogenous agonists	L‐glutamic acid [1574]	L‐glutamic acid [1574]	L‐glutamic acid [1574], NAAG [1750]	L‐glutamic acid [1574]	L‐glutamic acid [1574]
Agonists	–	–	–	L‐AP4 [2138], L‐serine‐O‐phosphate [2138]	–
Selective agonists	–	–	–	LSP4‐2022 [666]	(S)‐(+)‐CBPG (Partial agonist) [1261] – Rat, CHPG [1407]
Antagonists	LY367385 (pIC_50_ 5.1) [364]	–	–	MAP4 (p*K*_i_ 4.6) [721] – Rat	–
Selective antagonists	3‐MATIDA (pIC_50_ 5.2) [1386] – Rat, (S)‐(+)‐CBPG (pIC_50_ 4.2) [1261] – Rat, (S)‐TBPG (pIC_50_ 4.2) [381] – Rat, AIDA (pA_2_ 4.2) [1387]	PCCG‐4 (pIC_50_ 5.1) [1551] – Rat	–	–	ACDPP (pIC_50_ 6.9) [186]
Allosteric modulators	–	CBiPES (Positive) (pEC_50_ 7) [917], 4‐MPPTS (Positive) (pIC_50_ 5.8) [100, 916, 917, 1731]	–	SIB‐1893 (Positive) (pEC_50_ 6.3–6.8) [1281], MPEP (Positive) (pEC_50_ 6.3–6.6) [1281], PHCCC (Positive) (pEC_50_ 4.5) [1247]	3,3'‐difluorobenzaldazine (Positive) (pIC_50_ 5.6–8.5) [1481, 1482], alloswitch‐1 (Negative) (pIC_50_ 8.1) [1583] – Rat, CDPPB (Positive) (pEC_50_ 7.6–8) [1002, 1180], MTEP (Negative) (p*K*_i_ 7.8) [228], MPEP (Negative) (pIC_50_ 7.4–7.7) [609, 611], fenobam (Negative) (pIC_50_ 7.2) [1592], SIB‐1893 (Negative) (pIC_50_ 5.9–6.5) [609, 2028], SIB‐1757 (Negative) (pIC_50_ 6–6.4) [609, 2028], CPPHA (Positive) (pIC_50_ 6.3) [1482]
Selective allosteric modulators	BAY 367620 (Negative) (p*K*_i_ 9.5) [279] – Rat, JNJ16259685 (Negative) (pIC_50_ 8.9) [1104], Ro01‐6128 (Positive) (p*K*_i_ 7.5–7.7) [1019] – Rat, LY456236 (Negative) (pIC_50_ 6.9) [1160], CPCCOEt (Negative) (pIC_50_ 5.2–5.8) [1183]	Ro64‐5229 (Negative) (pIC_50_ 7) [1031] – Rat, biphenylindanone A (Positive) (pEC_50_ 7) [187]	–	VU0361737 (Positive) (pEC_50_ 6.6) [508], VU0155041 (Positive) (pEC_50_ 6.1) [1468]	VU‐1545 (Positive) (pEC_50_ 8) [429]

**Table:** 

Nomenclature	mGlu_6_ receptor	mGlu_7_ receptor	mGlu_8_ receptor
HGNC, UniProt	GRM6, O15303	GRM7, Q14831	GRM8, O00222
Endogenous agonists	L‐glutamic acid [1574]	L‐glutamic acid [1574]	L‐serine‐O‐phosphate [1254, 2138], L‐glutamic acid [1574]
Agonists	–	LSP4‐2022 [666], L‐serine‐O‐phosphate [2138], L‐AP4 [2138]	(S)‐3,4‐DCPG [1952], L‐AP4 [1254]
Selective agonists	1‐benzyl‐APDC [1987] – Rat, homo‐AMPA [244]	–	–
Antagonists	MAP4 (pIC_50_ 3.5) [1575] – Rat, THPG [1956]	–	MPPG (pIC_50_ 4.3) [2138]
Allosteric modulators	–	MMPIP (Negative) (pIC_50_ 6.1–7.6) [1467, 1900] – Rat, ADX71743 (Negative) (pIC_50_ 7.2) [938], AMN082 (Positive) (pEC_50_ 6.5–6.8) [1349], XAP044 (Negative) (pIC_50_ 5.6) [618]	–

### Comments

The activity of NAAG as an agonist at mGlu_3_ receptors was questioned on the basis of contamination with glutamate [341, 576], but this has been refuted [1430].

Radioligand binding using a variety of radioligands has been conducted on recombinant receptors (for example, [^3^ H]R214127[1103] and [^3^ H]YM298198[1025] at mGlu_1_ receptors and [^3^ H]M‐MPEP[609] and [^3^ H]methoxymethyl‐MTEP[48] at mGlu_5_ receptors. Although a number of radioligands have been used to examine binding in native tissues, correlation with individual subtypes is limited. Many pharmacological agents have not been fully tested across all known subtypes of mGlu receptors. Potential differences linked to the species (e.g. human versus rat or mouse) of the receptors and the receptor splice variants are generally not known. The influence of receptor expression level on pharmacology and selectivity has not been controlled for in most studies, particularly those involving functional assays of receptor coupling.

(S)‐(+)‐CBPG is an antagonist at mGlu_1_, but is an agonist (albeit of reduced efficacy) at mGlu_5_ receptors. DCG‐IV also exhibits agonist activity at NMDA glutamate receptors [2007], and is an antagonist at all Group‐III mGluRs with an IC_50_ of 30μM. A potential novel metabotropic glutamate receptor coupled to phosphoinositide turnover has been observed in rat brain; it is activated by 4‐methylhomoibotenic acid (ineffective as an agonist at recombinant Group I metabotropic glutamate receptors), but is resistant to LY341495[356]. There are also reports of a distinct metabotropic glutamate receptor coupled to phospholipase D in rat brain, which does not readily fit into the current classification [1013, 1549]

A related class C receptor composed of two distinct subunits, T1R1 + T1R3 is also activated by glutamate and is responsible for umami taste detection.

All selective antagonists at metabotropic glutamate receptors are competitive.

### Further reading on Metabotropic glutamate receptors

Conn PJ et al. (1997) Pharmacology and functions of metabotropic glutamate receptors. Annu. Rev. Pharmacol. Toxicol. 37: 205‐237 [PMID:9131252]

Ferraguti F et al. (2006) Metabotropic glutamate receptors. Cell Tissue Res. 326: 483‐504 [PMID:16847639]

Nicoletti F et al. (2011) Metabotropic glutamate receptors: from the workbench to the bedside. Neuropharmacology 60: 1017‐41 [PMID:21036182]

Niswender CM et al. (2010) Metabotropic glutamate receptors: physiology, pharmacology, and disease. Annu. Rev. Pharmacol. Toxicol. 50: 295‐322 [PMID:20055706]

Pin JP et al. (2016) Organization and functions of mGlu and GABAB receptor complexes. Nature 540: 60‐68 [PMID:27905440]

Rondard P et al. (2011) The complexity of their activation mechanism opens new possibilities for the modulation of mGlu and GABAB class C G protein‐coupled receptors. Neuropharmacology 60: 82‐92 [PMID:20713070]

## Motilin receptor

### Overview

Motilin receptors (provisional nomenclature) are activated by motilin(MLN, P12872), a 22 amino‐acid peptide derived from a precursor (MLN, P12872), which may also generate a motilin‐associated peptide (MLN, P12872). These receptors promote gastrointestinal motility and are suggested to be responsible for the gastrointestinal prokinetic effects of certain macrolide antibiotics (often called motilides; e.g. erythromycin), although for many of these molecules the evidence is sparse.

**Table:** 

Nomenclature	motilin receptor
HGNC, UniProt	MLNR, O43193
Endogenous agonists	motilin (MLN, P12872) [386, 1286, 1287, 1288]
Agonists	alemcinal [1947], erythromycin‐A [533, 1947], azithromycin [225]
Selective agonists	camicinal [105, 1712], mitemcinal [1023, 1918] – Rabbit
Selective antagonists	MA‐2029 (pA_2_ 9.2) [1884], GM‐109 (pIC_50_ 8) [736] – Rabbit
Labelled ligands	[^125^ I]motilin (human) (Agonist) [533]

### Comments

In terms of structure, the motilin receptor has closest homology with the ghrelin receptor. Thus, the human motilin receptor shares 52% overall amino acid identity with the ghrelin receptor and 86% in the transmembrane regions [759, 1918, 1947]. However, differences between the N‐terminus regions of these receptors means that their cognate peptide ligands do not readily activate each other [408, 1712]. In laboratory rodents, the gene encoding the motilin percursor appears to be absent, while the receptor appears to be a pseudogene [759, 1710]. Functions of motilin(MLN, P12872) are not usually detected in rodents, although brain and other responses to motilin and the macrolide alemcinal have been reported and the mechanism of these actions is obscure [1311, 1462]. Notably, in some non‐laboratory rodents (e.g. the North American kangaroo rat (Dipodomys) and mouse (Microdipodops) a functional form of motilin may exist but the motilin receptor is non‐functional [1159]. Marked differences in ligand affinities for the motilin receptor in dogs and humans may be explained by significant differences in receptor structure [1711]. Note that for the complex macrolide structures, selectivity of action has often not been rigorously examined and other actions are possible (e.g. P2X inhibition by erythromycin; [2216]). Small molecule motilin receptor agonists are now described [1159, 1712, 2100]. The motilin receptor does not appear to have constitutive activity [812]. Although not proven, the existence of biased agonism at the receptor has been suggested [1288, 1348, 1709]. A truncated 5‐transmembrane structure has been identified but this is without activity when transfected into a host cell [533]. Receptor dimerisation has not been reported.

### Further reading on Motilin receptor

De Smet B et al. (2009) Motilin and ghrelin as prokinetic drug targets. Pharmacol. Ther. 123: 207‐23 [PMID:19427331]

Sanger GJ et al. (2016) Ghrelin and motilin receptors as drug targets for gastrointestinal disorders. Nat Rev Gastroenterol Hepatol 13: 38‐48 [PMID:26392067]

## Neuromedin U receptors

### Overview

Neuromedin U receptors (provisional nomenclature as recommended by NC‐IUPHAR[557]) are activated by the endogenous 25 amino acid peptide neuromedin U (neuromedin U‐25 (NMU, P48645), NmU‐25), a peptide originally isolated from pig spinal cord [1344]. In humans, NmU‐25 appears to be the sole product of a precursor gene (NMU, P48645) showing a broad tissue distribution, but which is expressed at highest levels in the upper gastrointestinal tract, CNS, bone marrow and fetal liver. Much shorter versions of NmU are found in some species, but not in human, and are derived at least in some instances from the proteolytic cleavage of the longer NmU. Despite species differences in NmU structure, the C‐terminal region (particularly the C‐terminal pentapeptide) is highly conserved and contains biological activity. Neuromedin S (neuromedin S‐33 (NMS, Q5H8A3)) has also been identified as an endogenous agonist [1378]. NmS‐33 is, as its name suggests, a 33 amino‐acid product of a precursor protein derived from a single gene and contains an amidated C‐terminal heptapeptide identical to NmU. NmS‐33 appears to activate NMU receptors with equivalent potency to NmU‐25.

**Table:** 

Nomenclature	NMU1 receptor	NMU2 receptor
HGNC, UniProt	NMUR1, Q9HB89	NMUR2, Q9GZQ4
Antagonists	–	R‐PSOP (p*K*_B_ 7) [1193]

### Comments

NMU1 and NMU2 couple predominantly to G_q/11_ although there is evidence of good coupling to G_i/o_[218, 825, 833]. NMU1 and NMU2 can be labelled with [^125^I]‐NmU and [^125^I]‐NmS (of various species, e.g. [1319]), BODIPY ^®^ TMR‐NMU or Cy3B‐NMU‐8[218]. A range of radiolabelled (^125^I‐), fluorescently labelled (e.g. Cy3, Cy5, rhodamine and FAM) and biotin labelled versions of neuromedin U‐25(NMU, P48645) and neuromedin S‐33 (NMS, Q5H8A3) are now commercially available.

### Further reading on Neuromedin U receptors

Brighton PJ et al. (2004) Neuromedin U and its receptors: structure, function, and physiological roles. Pharmacol. Rev. 56: 231‐48 [PMID:15169928]

Budhiraja S et al. (2009) Neuromedin U: physiology, pharmacology and therapeutic potential. Fundam Clin Pharmacol 23: 149‐57 [PMID:19645813]

Mitchell JD et al. (2009) Emerging pharmacology and physiology of neuromedin U and the structurally related peptide neuromedin S. Br. J. Pharmacol. 158: 87‐103 [PMID:19519756]

Novak CM. (2009) Neuromedin S and U. Endocrinology 150: 2985‐7 [PMID:19549882]

## Neuropeptide FF/neuropeptide AF receptors

### Overview

The Neuropeptide FF receptor family contains two subtypes, NPFF1 and NPFF2 (provisional nomenclature [557]), which exhibit high affinities for neuropeptide FF (NPFF, O15130) and RFamide related peptides (RFRP: precursor gene symbol NPVF, Q9HCQ7). NPFF1 is broadly distributed in the central nervous system with the highest levels found in the limbic system and the hypothalamus. NPFF2 is present in high density in the superficial layers of the mammalian spinal cord where it is involved in nociception and modulation of opioid functions.

**Table:** 

Nomenclature	NPFF1 receptor	NPFF2 receptor
HGNC, UniProt	NPFFR1, Q9GZQ6	NPFFR2, Q9Y5X5
Potency order of endogenous ligands	RFRP‐1 (NPVF, Q9HCQ7) >RFRP‐3 (NPVF, Q9HCQ7) > FMRFneuropeptide FF (NPFF, O15130) >neuropeptide AF (NPFF, O15130) >neuropeptide SF (NPFF, O15130), QRFP43 (QRFP, P83859), PrRP‐31 (PRLH, P81277) [663]	neuropeptide AF (NPFF, O15130), neuropeptide FF (NPFF, O15130) >PrRP‐31 (PRLH, P81277) > FMRF, QRFP43 (QRFP, P83859) >neuropeptide SF (NPFF, O15130) [663]
Endogenous agonists	neuropeptide FF (NPFF, O15130) [663, 664, 1359], RFRP‐3 (NPVF, Q9HCQ7) [664, 665, 1359]	neuropeptide FF (NPFF, O15130) [664, 1358]
Selective agonists	–	dNPA [1681], AC263093 [1092]
Antagonists	RF9 (p*K*_i_ 7.2) [1814]	–
Selective antagonists	AC262620 (p*K*_i_ 7.7–8.1) [1092], AC262970 (p*K*_i_ 7.4–8.1) [1092]	–
Labelled ligands	[^125^ I]Y‐RFRP‐3 (Agonist) [664], [^3^ H]NPVF (Agonist) [1928], [^125^ I]NPFF (Agonist) [663]	[^125^ I]EYF (Agonist) [1359], [^3^ H]EYF (Agonist) [1928], [^125^ I]NPFF (Agonist) [663]

### Comments

An orphan receptor GPR83(Q9NYM4) shows sequence similarities with NPFF1, NPFF2, PrRP and QRFP receptors. The antagonist RF9 is selective for NPFF receptors, but does not distinguish between the NPFF1 and NPFF2 subtypes (pK_i_ 7.1 and 7.2, respectively, [1814]).

### Further reading on Neuropeptide FF/neuropeptide AF receptors

Moulédous L et al. (2010) Opioid‐modulating properties of the neuropeptide FF system. Biofactors 36: 423‐9 [PMID:20803521]

Vyas N et al. (2006) Structure‐activity relationships of neuropeptide FF and related peptidic and non‐peptidic derivatives. Peptides 27: 990‐6 [PMID:16490282]

Yang HY et al. (2008) Modulatory role of neuropeptide FF system in nociception and opiate analgesia. Neuropeptides 42: 1‐18 [PMID:17854890]

## Neuropeptide S receptor

### Overview

The neuropeptide S receptor (NPS, provisional nomenclature [557]) responds to the 20 amino‐acid peptide neuropeptide S derived from a precursor (NPS, P0C0P6).

**Table:** 

Nomenclature	NPS receptor
HGNC, UniProt	NPSR1, Q6W5P4
Endogenous agonists	neuropeptide S (NPS, P0C0P6) [2159]
Selective agonists	PWT1‐NPS [1692] – Mouse
Selective antagonists	NCGC 84 (pA_2_ 9) [1957], SHA 68 (pA_2_ 8.1) [1693] – Mouse, RTI‐118 [2214]
Labelled ligands	[^125^ I]Tyr ^10^NPS (human) (Agonist) [2159]

### Comments

Multiple single‐nucleotide polymorphisms (SNP) and several splice variants have been identified in the human NPS receptor. The most interesting of these is an Asn‐Ile exchange at position 107 (Asn^107^Ile). The human NPS receptor Asn^107^Ile displayed similar binding affinity but higher NPS potency (by approx. 10‐fold) than human NPS receptor Asn107 [1645]. Several epidemiological studies reported an association between Asn^107^Ile receptor variant and susceptibility to panic disorders [458, 460, 1506, 1621]. The SNP Asn^107^Ile has also been linked to sleep behavior [662], inflammatory bowel disease [402], schizophrenia [1145], increased impulsivity and ADHD symptoms [1083]. Interestingly, a carboxy‐terminal splice variant of human NPS receptor was found to be overexpressed in asthmatic patients [1091].

### Further reading on Neuropeptide S receptor

Guerrini R et al. (2010) Neurobiology, pharmacology, and medicinal chemistry of neuropeptide S and its receptor. Med Res Rev 30: 751‐77 [PMID:19824051]

Xu YL et al. (2004) Neuropeptide S: a neuropeptide promoting arousal and anxiolytic‐like effects. Neuron 43: 487‐497 [PMID:15312648]

## Neuropeptide W/neuropeptide B receptors

### Overview

The neuropeptide BW receptor 1 (NPBW1, provisional nomenclature [557]) is activated by two 23‐amino‐acid peptides, neuropeptide W (neuropeptide W‐23 (NPW, Q8N729)) and neuropeptide B (neuropeptide B‐23 (NPB, Q8NG41)) [584, 1792]. C‐terminally extended forms of the peptides (neuropeptide W‐30(NPW, Q8N729) and neuropeptide B‐29 (NPB, Q8NG41)) also activate NPBW1 [216]. Unique to both forms of neuropeptide B is the N‐terminal bromination of the first tryptophan residue, and it is from this post‐translational modification that the nomenclature NPB is derived. These peptides were first identified from bovine hypothalamus and therefore are classed as neuropeptides. Endogenous variants of the peptides without the N‐terminal bromination, des‐Br‐neuropeptide B‐23(NPB, Q8NG41) and des‐Br‐neuropeptide B‐29 (NPB, Q8NG41), were not found to be major components of bovine hypothalamic tissue extracts. The NPBW2 receptor is activated by the short and C‐terminal extended forms of neuropeptide W and neuropeptide B [216].

**Table:** 

Nomenclature	NPBW1 receptor	NPBW2 receptor
HGNC, UniProt	NPBWR1, P48145	NPBWR2, P48146
Potency order of endogenous ligands	neuropeptide B‐29 (NPB, Q8NG41) >neuropeptide B‐23 (NPB, Q8NG41) >neuropeptide W‐23 (NPW, Q8N729) >neuropeptide W‐30 (NPW, Q8N729) [216]	neuropeptide W‐23 (NPW, Q8N729) >neuropeptide W‐30 (NPW, Q8N729) >neuropeptide B‐29 (NPB, Q8NG41) >neuropeptide B‐23 (NPB, Q8NG41) [216]
Selective agonists	Ava3 [945], Ava5 [945]	–
Labelled ligands	[^125^ I]NPW‐23 (human) (Agonist) [1816]	[^125^ I]NPW‐23 (human) (Agonist) [1792]

### Comments

Potency measurements were conducted with heterologously‐expressed receptors with a range of 0.14‐0.57 nM (NPBW1) and 0.98‐21 nM (NPBW2). NPBW1^‐/‐^ mice show changes in social behavior, suggesting that the NPBW1 pathway may have an important role in the emotional responses of social interaction [1414]. For a review of the contribution of neuropeptideB/W to social dominance, see [2080]. It has been reported that neuropeptide W may have a key role in the gating of stressful stimuli when mice are exposed to novel environments [1392]. Two antagonists have been discovered and reported to have affinity for NPBW1, ML181 and ML250, the latter exhibiting improved selectivity ( ∼ 100 fold) for NPBW1 compared to MCH1 receptors [694, 695]. Computational insights into the binding of antagonists to this receptor have also been described [1541].

### Further reading on Neuropeptide W/neuropeptide B receptors

Sakurai T. (2013) NPBWR1 and NPBWR2: Implications in Energy Homeostasis, Pain, and Emotion. Front Endocrinol (Lausanne) 4: 23 [PMID:23515889]

Singh G et al. (2006) Neuropeptide B and W: neurotransmitters in an emerging G‐protein‐coupled receptor system. Br. J. Pharmacol. 148: 1033‐41 [PMID:16847439]

## Neuropeptide Y receptors

### Overview

Neuropeptide Y (NPY) receptors (nomenclature as agreed by the NC‐IUPHAR Subcommittee on Neuropeptide Y Receptors [1330]) are activated by the endogenous peptides neuropeptide Y (NPY, P01303), neuropeptide Y‐(3‐36), peptide YY(PYY, P10082), PYY‐(3‐36) and pancreatic polypeptide (PPY, P01298) (PP). The receptor originally identified as the Y3 receptor has been identified as the CXCR4 chemokine recepter (originally named LESTR, [1201]). The y6 receptor is a functional gene product in mouse, absent in rat, but contains a frame‐shift mutation in primates producing a truncated non‐functional gene [676]. Many of the agonists exhibit differing degrees of selectivity dependent on the species examined. For example, the potency of PP is greater at the rat Y_4_receptor than at the human receptor [513]. In addition, many agonists lack selectivity for individual subtypes, but can exhibit comparable potency against pairs of NPY receptor subtypes, or have not been examined for activity at all subtypes. [^125^I]‐PYY or [^125^I]‐NPY can be used to label Y_1_, Y_2_, Y_5_ and y_6_ subtypes non‐selectively, while [^125^ I][cPP(1‐7), NPY(19‐23), Ala ^31^, Aib^32^, Gln^34^]hPP may be used to label Y_5_ receptors preferentially (note that cPP denotes chicken peptide sequence and hPP is the human sequence).

**Table:** 

Nomenclature	Y_1_ receptor	Y_2_ receptor	Y_4_ receptor	Y_5_ receptor	y_6_ receptor
HGNC, UniProt	NPY1R, P25929	NPY2R, P49146	NPY4R, P50391	NPY5R, Q15761	NPY6R, Q99463
Potency order of endogenous ligands	neuropeptide Y = peptide YY ≫ pancreatic polypeptide	peptide YY = peptide YY(3‐36) = neuropeptide Y = neuropeptide Y(3‐36) ≫ pancreatic polypeptide	pancreatic polypeptide ≫ neuropeptide Y = peptide YY	neuropeptide Y > peptide YY > pancreatic polypeptide	neuropeptide Y = peptide YY > pancreatic polypeptide
Endogenous agonists	neuropeptide Y (NPY, P01303), peptide YY (PYY, P10082)	PYY‐(3‐36) (PYY, P10082) [619, 633], neuropeptide Y (NPY, P01303), neuropeptide Y‐(3‐36) (NPY, P01303), peptide YY (PYY, P10082)	pancreatic polypeptide (PPY, P01298) [98, 1217, 1978, 2165]	–	–
Agonists	[Leu ^31^,Pro^34^]NPY [392], [Leu ^31^,Pro^34^]PYY (human), [Pro ^34^]NPY, [Pro ^34^]PYY (human)	–	–	–	–
Selective agonists	–	–	–	[Ala ^31^,Aib^32^]NPY (pig) [264]	–
Selective antagonists	BIBO3304 (pIC_50_ 9.5) [2110], BIBP3226 (p*K*_i_ 8.1–9.3) [463, 2111]	BIIE0246 (pIC_50_ 8.5) [461], JNJ‐5207787 (pIC_50_ 6.9–7.1) [182]	–	L‐152,804 (p*K*_i_ 7.6) [944]	–
Selective allosteric modulators	–	–	niclosamide (Positive) [1827]	–	–
Labelled ligands	[^3^ H]BIBP3226 (Antagonist) (p*K*_d_ 8.7), [^125^ I][Leu ^31^,Pro^34^]NPY (Agonist)	[^125^ I]PYY‐(3‐36) (human) (Agonist)	[^125^ I]PP (human) (Agonist)	[^125^ I][cPP(1‐7), NPY(19‐23), Ala ^31^, Aib^32^, Gln^34^]hPP (Agonist) [481] – Rat	–
Comments	Note that Pro^34^‐containing NPY and PYY can also bind Y_4_ and Y_5_ receptors, so strictly speaking are not selective, but are the 'preferred' agonists.	–	–	–	–

### Comments

The Y_1_ agonists indicated are selective relative to Y_2_ receptors. BIBP3226 is selective relative to Y_2_, Y_4_ and Y_5_ receptors [632]. NPY‐(13‐36) is Y_2_ selective relative to Y_1_ and Y_5_ receptors. PYY‐(3‐36) is Y_2_ selective relative to Y_1_ receptors. Note that Pro34‐containing NPY and PYY can also bind Y_4_ and Y_5_, thus they are selective only relative to Y_2_. The y_6_ receptor is a pseudogene in humans, but is functional in mouse, rabbit and some other mammals.

### Further reading on Neuropeptide Y receptors

Bowers ME et al. (2012) Neuropeptide regulation of fear and anxiety: Implications of cholecystokinin, endogenous opioids, and neuropeptide Y. Physiol. Behav. 107: 699‐710 [PMID:22429904]

Michel MC et al. (1998) XVI. International Union of Pharmacology recommendations for the nomenclature of neuropeptide Y, peptide YY and pancreatic polypeptide receptors. Pharmacol. Rev. 50: 143‐150 [PMID:9549761]

Pedragosa‐Badia X et al. (2013) Neuropeptide Y receptors: how to get subtype selectivity. Front Endocrinol (Lausanne) 4: 5 [PMID:23382728]

Zhang L et al. (2011) The neuropeptide Y system: pathophysiological and therapeutic implications in obesity and cancer. Pharmacol. Ther. 131: 91‐113 [PMID:21439311]

## Neurotensin receptors

### Overview

Neurotensin receptors (nomenclature as recommended by NC‐IUPHAR[557]) are activated by the endogenous tridecapeptide neurotensin (pGlu‐Leu‐Tyr‐Glu‐Asn‐Lys‐Pro‐Arg‐Arg‐Pro‐Tyr‐Ile‐Leu) derived from a precursor (NTS, 30990), which also generates neuromedin N, an agonist at the NTS_2_ receptor. [^3^ H]neurotensin (human, mouse, rat) and [^125^ I]neurotensin (human, mouse, rat) may be used to label NTS_1_ and NTS_2_ receptors at 0.1‐0.3 and 3‐5 nM concentrations respectively.

**Table:** 

Nomenclature	NTS_1_ receptor	NTS_2_ receptor
HGNC, UniProt	NTSR1, P30989	NTSR2, O95665
Potency order of endogenous ligands	neurotensin (NTS, P30990) >neuromedin N {Mouse, Rat} [776]	neurotensin (NTS, P30990) = neuromedin N {Mouse, Rat} [1297]
Selective agonists	JMV449 [1822] – Rat	levocabastine [1297, 1657]
Selective antagonists	meclinertant (pIC_50_ 7.5–8.2) [699]	–
Labelled ligands	[^3^ H]meclinertant (Antagonist) (p*K*_d_ 8.5) [1085] – Rat	–

### Comments

neurotensin(NTS, P30990) appears to be a low‐efficacy agonist at the NTS_2_ receptor [2039], while the NTS_1_ receptor antagonist meclinertant is an agonist at NTS_2_ receptors [2039]. An additional protein, provisionally termed NTS_3_ (also known as NTR3, gp95 and sortilin; ENSG00000134243), has been suggested to bind lipoprotein lipase and mediate its degradation [1460]. It has been reported to interact with the NTS_1_ receptor [1273] and the NTS_2_ receptor [260], and has been implicated in hormone trafficking and/or neurotensin uptake. A splice variant of the NTS_2_ receptor bearing 5 transmembrane domains has been identified in mouse [195] and later in rat [1561].

### Further reading on Neurotensin receptors

Boules M et al. (2013) Diverse roles of neurotensin agonists in the central nervous system. Front Endocrinol (Lausanne) 4: 36 [PMID:23526754]

Mazella J et al. (2012) Neurotensin and its receptors in the control of glucose homeostasis. Front Endocrinol (Lausanne) 3: 143 [PMID:23230428]

Myers RM et al. (2009) Cancer, chemistry, and the cell: molecules that interact with the neurotensin receptors. ACS Chem. Biol. 4: 503‐25 [PMID:19462983]

Tanganelli S et al. (2012) Relevance of dopamine D(2)/neurotensin NTS1 and NMDA/neurotensin NTS1 receptor interaction in psychiatric and neurodegenerative disorders. Curr. Med. Chem. 19: 304‐16 [PMID:22335510]

## Opioid receptors

### Overview

Opioid and opioid‐like receptors are activated by a variety of endogenous peptides including [Met]enkephalin(PENK, P01210) (met), [Leu]enkephalin(PENK, P01210) (leu), *β* ‐endorphin(POMC, P01189) (*β*‐end), *α* ‐neodynorphin(PDYN, P01213), dynorphin A(PDYN, P01213) (dynA), dynorphin B(PDYN, P01213) (dynB), big dynorphin(PDYN, P01213) (Big dyn), nociceptin/orphanin FQ(PNOC, Q13519) (N/OFQ); endomorphin‐1 and endomorphin‐2 are also potential endogenous peptides. The Greek letter nomenclature for the opioid receptors, *μ*, *δ* and *κ*, is well established, and NC‐IUPHAR considers this nomenclature appropriate, along with the symbols spelled out (mu, delta, and kappa), and the acronyms, MOP, DOP, and KOP. [390, 441, 557]. The human N/OFQ receptor, NOP, is considered 'opioid‐related' rather than opioid because, while it exhibits a high degree of structural homology with the conventional opioid receptors [1361], it displays a distinct pharmacology. Currently there are numerous clinically used drugs, such as morphine and many other opioid analgesics, as well as antagonists such as naloxone, however only for the *μ* receptor.

**Table:** 

Nomenclature	*δ* receptor	*κ* receptor	*μ* receptor	NOP receptor
HGNC, UniProt	OPRD1, P41143	OPRK1, P41145	OPRM1, P35372	OPRL1, P41146
Principal endogenous agonists	*β* ‐endorphin (POMC, P01189), [Leu]enkephalin (PENK, P01210), [Met]enkephalin (PENK, P01210)	big dynorphin (PDYN, P01213), dynorphin A (PDYN, P01213)	*β* ‐endorphin (POMC, P01189), [Met]enkephalin (PENK, P01210), [Leu]enkephalin (PENK, P01210)	nociceptin/orphanin FQ (PNOC, Q13519) [11, 153, 1507]
Potential endogenous agonists	–	–	endomorphin‐1, endomorphin‐2	–
Agonists	DADLE [1972], etorphine [1972], ethylketocyclazocine [1972]	–	levorphanol [727], hydromorphone [2094], fentanyl [1972], buprenorphine (Partial agonist) [1972], methadone [1595], codeine [1972], tapentadol [1992], pethidine [1595]	–
Sub/family‐selective agonists	BU08028 (Partial agonist) [979]	BU08028 [979]	BU08028 (Partial agonist) [979]	cebranopadol [1182], BU08028 (Partial agonist) [979]
Selective agonists	UFP‐512 [2033], BW373U86 [1115], ADL5859 [1115], DPDPE [1391, 1972], [D‐Ala ^2^]deltorphin II [515], ADL5747 [1116], SNC80 [268, 1620]	U50488 [313, 1545, 1813, 1972, 2046, 2222, 2224], enadoline [848, 1447], U69593 [1089, 1972], salvinorin A [259, 1677]	sufentanil [2041], DAMGO [726, 1972], loperamide [323], morphine [653, 1972], PL017 [304, 1972]	N/OFQ‐(1‐13)‐NH_2_ [153, 696, 1304, 1507], Ac‐RYYRWK‐NH_2_ (Partial agonist) [464, 1304], SCH221510 [2030], Ro64‐6198 [898, 2108]
Antagonists	naltrexone (p*K*_i_ 8) [1972], naloxone (p*K*_i_ 7.2) [1972]	buprenorphine (p*K*_i_ 9.1–10.2) [1972, 2224], nalmefene (p*K*_i_ 9.5) [1972], naltrexone (p*K*_i_ 8.4–9.4) [1545, 1813, 1972], naloxone (p*K*_i_ 7.6–8.6) [1545, 1813, 1972, 2222, 2224]	naltrexone (p*K*_i_ 9.1–9.7) [965, 1972], nalmefene (p*K*_i_ 9.5) [1972], nalorphine (p*K*_i_ 8.9) [1972], naloxone (p*K*_i_ 8.9) [1972], methylnaltrexone (p*K*_i_ 8.7) [2094]	–
Sub/family‐selective antagonists	AT‐076 (p*K*_i_ 7.7) [1972, 2201]	AT‐076 (p*K*_i_ 8.9) [1972, 2202]	AT‐076 (p*K*_i_ 8.8) [1972, 2202]	AT‐076 (p*K*_i_ 8.8) [2202]
Selective antagonists	naltriben (p*K*_i_ 10) [1841, 1972], naltrindole (p*K*_i_ 9.7) [1594, 1972], TIPP *ψ* (Inverse agonist) (p*K*_i_ 9) [1735, 1972]	nor‐binaltorphimine (p*K*_i_ 8.9–11) [1545, 1593, 1813, 1972, 2222, 2224], 5'‐guanidinonaltrindole (p*K*_i_ 9.7–9.9) [924, 1545, 1868], JDTic (p*K*_i_ 9–9.4) [1400, 1951, 2202]	alvimopan (p*K*_i_ 9.3) [1114], levallorphan (p*K*_i_ 8.8–9.3) [1250], CTAP (p*K*_i_ 8.6) [304, 1972]	UFP‐101 (p*K*_i_ 10.2) [269], LY2940094 (p*K*_i_ 10) [1971], compound 24 (p*K*_i_ 9.6) [549], SB 612111 (p*K*_i_ 9.2–9.5) [1856, 2200], J‐113397 (pIC_50_ 8.3) [962]
Allosteric modulators	–	–	BMS‐986123 (Neutral) (p*K*_B_ 6) [247], BMS‐986121 (Positive) (p*K*_B_ 5.7) [247], BMS‐986124 (Neutral) (p*K*_B_ 5.7) [247], BMS‐986122 (Positive) (p*K*_B_ 5.3) [247]	–
Labelled ligands	[^3^ H]naltrindole (Antagonist) (p*K*_d_ 10.4) [2161] – Rat, [^3^ H][D‐Ala2]deltorphin I (Selective Agonist) [1865], [^3^ H]diprenorphine (Agonist) [52, 1972], [^3^ H]DPDPE (Agonist) [26], [^3^ H]deltorphin II (Agonist) [261], [^3^ H]naltriben (Antagonist) [1154]	[^3^ H]diprenorphine (Antagonist) (p*K*_d_ 9.1) [52, 1813], [^3^ H]U69593 (Agonist) [1089, 1545, 1813], [^3^ H]enadoline (Agonist) [1815]	[^3^ H]diprenorphine (Antagonist) (p*K*_d_ 10.1) [1638] – Mouse, [^3^ H]DAMGO (Agonist) [1638] – Rat, [^3^ H]PL017 (Agonist) [751] – Rat	[^3^ H]N/OFQ (Agonist) [464, 1360]

### Comments

Three naloxone‐sensitive opioid receptor genes have been identified in humans, and while the *μ*‐receptor in particular may be subject to extensive alternative splicing [1535], these putative isoforms have not been correlated with any of the subtypes of receptor proposed in years past. Opioid receptors may heterodimerize with each other or with other 7TM receptors [926], and give rise to complexes with a unique pharmacology, however, evidence for such heterodimers in native cells is equivocal and the consequences of this heterodimerization for signalling remains largely unknown. For *μ*‐opioid receptors at least, dimerization does not seem to be required for signalling [1078]. A distinct met‐enkephalin receptor lacking structural resemblance to the opioid receptors listed has been identified (OGFR, 9NZT2) and termed an opioid growth factor receptor [2198].

Endomorphin‐1 and endomorphin‐2 have been identified as highly selective, putative endogenous agonists for the *μ*‐opioid receptor. At present, however, the mechanisms for endomorphin synthesis in vivo have not been established, and there is no gene identified that encodes for either. Thus, the status of these peptides as endogenous ligands remains unproven.

Two areas of increasing importance in defining opioid receptor function are the presence of functionally relevant single nucleotide polymorphisms in human *μ*‐receptors [1490] and the identification of biased signalling by opioid receptor ligands, in particular, compounds previously characterized as antagonists [236]. Pathway bias for agonists makes general rank orders of potency and efficacy somewhat obsolete, so these do not appear in the table. As ever, the mechanisms underlying the acute and long term regulation of opiod receptor function are the subject of intense investigation and debate.

The richness of opioid receptor pharmacology has been enhanced with the recent discovery of allosteric modulators of *μ* and *δ* receptors, notably the positive allosteric modulators and silent allosteric "antagonists" outlined in [247, 248]. Negative allosteric modulation of opioid receptors has been previously suggested [953], whether all compounds are acting at a similar site remains to be established.

### Further reading on Opioid receptors

Butelman ER et al. (2012) *κ*‐opioid receptor/dynorphin system: genetic and pharmacotherapeutic implications for addiction. Trends Neurosci. 35: 587‐96 [PMID:22709632]

Cox BM et al. (2015) Challenges for opioid receptor nomenclature: IUPHAR Review 9. Br. J. Pharmacol. 172: 317‐23 [PMID:24528283]

Pradhan AA et al. (2011) The delta opioid receptor: an evolving target for the treatment of brain disorders. Trends Pharmacol. Sci. 32: 581‐90 [PMID:21925742]

Williams JT et al. (2013) Regulation of *μ*‐opioid receptors: desensitization, phosphorylation, internalization, and tolerance. Pharmacol. Rev. 65: 223‐54 [PMID:23321159]

## Orexin receptors

### Overview

Orexin receptors (nomenclature as agreed by the NC‐IUPHAR Subcommittee on Orexin receptors [557]) are activated by the endogenous polypeptides orexin‐A(HCRT, O43612) and orexin‐B(HCRT, O43612) (also known as hypocretin‐1 and ‐2; 33 and 28 aa) derived from a common precursor, preproorexin or orexin precursor, by proteolytic cleavage [1703].

**Table:** 

Nomenclature	OX_1_ receptor	OX_2_ receptor
HGNC, UniProt	HCRTR1, O43613	HCRTR2, O43614
Potency order of endogenous ligands	orexin‐A (HCRT, O43612) >orexin‐B (HCRT, O43612)	orexin‐A (HCRT, O43612) = orexin‐B (HCRT, O43612)
Selective agonists	–	[Ala ^11^, D‐Leu^15^]orexin‐B [66, 1612]
Selective antagonists	suvorexant (p*K*_i_ 9.3) [391], SB‐649868 (p*K*_i_ 9.1) [442], SB‐674042 (p*K*_i_ 8.7–9.1) [1098, 1253, 1255], filorexant (p*K*_i_ 8.6) [2124], almorexant (pIC_50_ 7.9) [221], SB‐408124 (p*K*_i_ 7.2–7.6) [1098, 1253], SB‐334867 (p*K*_i_ 7.4–7.5) [1253, 1591]	filorexant (p*K*_i_ 9.5) [2124], suvorexant (p*K*_i_ 9.5) [391], EMPA (p*K*_i_ 9) [1252], SB‐649868 (p*K*_i_ 8.9) [442], JNJ‐10397049 (p*K*_i_ 8–8.6) [1300], almorexant (pIC_50_ 8.1) [221], TCS‐OX2‐29 (p*K*_i_ 7.4) [798]
Labelled ligands	–	[^3^ H]‐almorexant (Selective Antagonist) (p*K*_d_ 8.9–9.8) [1253, 1255], [^3^ H]Cp‐1 (Selective Antagonist) (p*K*_d_ 9.2–9.4) [1253], [^3^ H]EMPA (Selective Antagonist) (p*K*_d_ 8.6–9) [1252, 1255], [^125^ I]‐orexin‐A (Agonist) [1066, 1611, 1703]

### Comments

The primary coupling of orexin receptors to G_q/11_ proteins is rather speculative and based on the strong activation of phospholipase C, though recent studies in recombinant CHO cells also stress the importance of G_q/11_[1065]. Coupling of both receptors to G_i/o_ and G_s_ has also been reported [951, 1068, 1146, 1629] for most cellular responses observed, the G protein pathway is unknown. The potency order of endogenous ligands may depend on the cellular signal transduction machinery. Most of the OX_2_ receptor selective antagonists listed are weakly selective (≤10‐fold), or selectivity may be less than 100‐fold or not unequivocally determined. [Ala ^11^, D‐Leu^15^]orexin‐B may show poor OX_2_ receptor selectivity [1612].

Orexin receptors have been reported to be able to form complexes with each other and some other GPCRs as well as CRF receptors [1067, 1426], which might affect the signaling and pharmacology. Recently a promising synthetic orexin receptor ligand (compound 26) has been reported but not thoroughly characterized [1412]. Loss‐of‐function mutations in the gene encoding the OX_2_ receptor underlie canine hereditary narcolepsy [1177]. Antagonists of the orexin receptors are the focus of major drug discovery effort for their potential to treat insomnia and other disorders of wakefulness [1668], while agonists would likely be useful in human narcolepsy.

### Further reading on Orexin receptors

Baimel C et al. (2015) Orexin/hypocretin role in reward: implications for opioid and other addictions. Br. J. Pharmacol. 172: 334‐48 [PMID:24641197]

Kukkonen JP. (2013) Physiology of the orexinergic/hypocretinergic system: a revisit in 2012. Am. J. Physiol., Cell Physiol. 304: C2‐32 [PMID:23034387]

Li SB et al. (2016) Hypocretins, Neural Systems, Physiology, and Psychiatric Disorders. Curr Psychiatry Rep 18: 7 [PMID:26733323]

Mahler SV et al. (2014) Motivational activation: a unifying hypothesis of orexin/hypocretin function. Nat. Neurosci. 17: 1298‐303 [PMID:25254979]

## Oxoglutarate receptor

### Overview

Nomenclature as recommended by NC‐IUPHAR[414].

**Table:** 

Nomenclature	oxoglutarate receptor
HGNC, UniProt	OXGR1, Q96P68
Endogenous agonists	*α* ‐ketoglutaric acid [762, 1854]

## P2Y receptors

### Overview

P2Y receptors (nomenclature as agreed by the NC‐IUPHAR Subcommittee on P2Y Receptors [1, 2]) are activated by the endogenous ligands ATP, ADP, uridine triphosphate, uridine diphosphate and UDP‐glucose. The relationship of many of the cloned receptors to endogenously expressed receptors is not yet established and so it might be appropriate to use wording such as 'uridine triphosphate‐preferring (or ATP‐, etc.) P2Y receptor' or 'P2Y_1_‐like', etc., until further, as yet undefined, corroborative criteria can be applied [251, 514, 878, 2044, 2089].

Clinically used drugs acting on these receptors include the dinucleoside polyphosphate diquafosol, agonist of the P2Y_2_ receptor subtype, approved in Japan for the management of dry eye disease [1101], and the P2Y_12_ receptor antagonists prasugrel, ticagrelor and cangrelor, all approved as antiplatelet drugs [273, 1602].

**Table:** 

Nomenclature	P2Y_1_ receptor	P2Y_2_ receptor	P2Y_4_ receptor	P2Y_6_ receptor
HGNC, UniProt	P2RY1, P47900	P2RY2, P41231	P2RY4, P51582	P2RY6, Q15077
Potency order of endogenous ligands	ADP>ATP	uridine triphosphate>ATP [1112]	uridine triphosphate>ATP (at rat recombinant receptors, UTP = ATP)	uridine diphosphate≫uridine triphosphate>ADP
Endogenous agonists	–	uridine triphosphate [989, 1112]	–	–
Agonists	ADP *β*S [1921], 2MeSADP [1729, 2054]	–	–	–
Sub/family‐selective agonists	–	diquafosol [1554], denufosol [1113, 1554, 2181], UTP *γ*S [1112]	diquafosol [240], denufosol [2181], UTP *γ*S [1113]	–
Selective agonists	MRS2365 [329], 2‐Cl‐ADP( *α*‐BH_3_) [76]	MRS2698 [874], 2‐thioUTP [498], PSB1114 (EC_50_ value determined using an IP_3_ functional assay) [498, 499, 873]	MRS4062 [1276], MRS2927 [1276], (N)methanocarba‐UTP [989]	Rp‐5‐OMe‐UDP *α*B [644, 702], MRS2957 [1275], MRS2693 [146]
Antagonists	suramin (p*K*_i_ 5.3) [2054], PPADS (p*K*_i_ 5.2) [2054]	–	ATP (p*K*_d_ 6.2) [970]	–
Sub/family‐selective antagonists	–	reactive blue‐2 (pIC_50_ 6) [892], suramin (pIC_50_ 4.3) [892, 1729]	PPADS (pEC_50_ 2–5) [881], reactive blue‐2 (pIC_50_ 4.7) [171] – Rat	reactive blue‐2 (p*K*_B_ 6) [2045], PPADS (p*K*_B_ 4) [2045], suramin (p*K*_B_ 4) [2045]
Selective antagonists	MRS2500 (p*K*_i_ 8.8–9.1) [286, 988], MRS2279 (p*K*_i_ 7.9) [2054], MRS2179 (p*K*_i_ 7–7.1) [203, 2054]	AR‐C118925XX (pIC_50_∼6) [968], AR‐C126313 (pEC_50_ 6) [874], PSB‐416 (pIC_50_ 4.7) [792]	ATP (p*K*_d_ 6.2) [970]	MRS2578 (pIC_50_ 7.4) [1257], MRS2567 (pIC_50_ 6.9) [1257]
Allosteric modulators	2,2'‐pyridylisatogen tosylate (Negative) (pIC_50_ 7.8) [601]	–	–	–
Selective allosteric modulators	BMS compound 16 (Negative) (p*K*_i_ 6.9) [2206]	–	–	–
Labelled ligands	[^3^ H]MRS2279 (Antagonist) (p*K*_d_ 8.1) [2054], [^3^ H]2MeSADP (Agonist) [1921], [^35^ S]ADP *β*S (Agonist)	–	–	MRS4162 (Selective Antagonist) (pEC_50_ 7.6) [897]

**Table:** 

Nomenclature	P2Y_11_ receptor	P2Y_12_ receptor	P2Y_13_ receptor	P2Y_14_ receptor
HGNC, UniProt	P2RY11, Q96G91	P2RY12, Q9H244	P2RY13, Q9BPV8	P2RY14, Q15391
Potency order of endogenous ligands	ATP	ADP [775]	ADP≫ATP	uridine diphosphate [281]
Sub/family‐selective agonists	–	2MeSADP [775], ADP *β*S [1921]	2MeSADP [1271], 2MeSATP [1271], ADP *β*S [1271]	–
Selective agonists	AR‐C67085 [93, 372], NF546 [1317], ATP *γ*S [372]	–	–	*α*.*β* ‐methylene‐2‐thio‐UDP [407], MRS2905 [879], 2‐thio‐UDP [407]
Antagonists	–	PSB‐0739 (p*K*_i_ 7.6) [97]	–	–
Sub/family‐selective antagonists	suramin (pIC_50_ 4.8–6) [372], reactive blue‐2 (pIC_50_ 5) [372]	cangrelor (pIC_50_ 9.4) [882], Ap_4_A (pIC_50_ 6) [1271], 2MeSAMP (pIC_50_ 5.4) [1921]	cangrelor (pIC_50_ 8.3) [1271], Ap_4_A (pIC_50_ 6.7) [1271], 2MeSAMP (pIC_50_ 5.6) [1271]	–
Selective antagonists	NF157 (p*K*_i_ 7.3) [1999], NF340 (pIC_50_ 6.4–7.1) [1317]	AZD1283 (p*K*_i_ 8) [79, 2207], ARL66096 (pIC_50_ 7.9) [846, 847], ticagrelor (p*K*_i_ 7.8) [2203]	MRS2603 (pIC_50_ 6.2) [996], MRS2211 (pIC_50_ 6) [996]	PPTN (p*K*_i_ 10.1) [102]
Labelled ligands	–	[^3^ H]2MeSADP (Agonist) [1921], [^3^ H]PSB‐0413 (Antagonist) (p*K*_d_ 8.3–8.5) [497, 1497]	[^33^ P]2MeSADP (Agonist) [1271]	MRS4174 (Selective Antagonist) (p*K*_i_ 10.1) [1006], MRS4183 (Selective Agonist) [1005]

### Comments

A series of 4‐alkyloxyimino derivatives of uridine‐5'‐triphosphate which could be useful for derivatization as fluorescent P2Y_2/4/6_ receptor probes has been recently synthesized [897].

Single nucleotide polymorphisms of the P2YR_1_ gene are associated with different platelet reactivity to ADP ADP[784]. Three frequent nonsynonymous P2Y_2_ receptor polymorphisms have been identified, one of which was significantly more common in cystic fibrosis patients. This polymorphism is linked to increases in Ca^2+^ influx in transfected cells, and might therefore play a role in disease development [263]. Although uridine triphosphate (UTP) was also shown to be a biased agonist at P2Y_11_, this is still under debate [1388, 2104]. A group of single nucleotide polymorphisms in the P2Y_12_ gene, forming the so called P2Y_12_ H2 haplotype, has been associated with increased platelet responsiveness to ADP, increased risk of peripheral arterial disease and with coronary artery disease [291]. The platelet‐type bleeding disorder due to P2Y_12_ receptor defects is an autosomal recessive condition characterized by mild to moderate mucocutaneous bleeding and excessive bleeding after surgery or trauma. The defect is due to the inability of ADP to induce platelet aggregation [287]. The P2Y_13_ receptor Met‐158‐Thr polymorphism, which is in linkage disequilibrium with the P2Y_12_ locus, is not associated with acute myocardial infarction, diabetes mellitus or related risk factors [44]. The P2Y_14_ receptor was previously considered to exclusively bind sugar nucleotides such as UDP‐glucose and UDP‐galactose[299]. However, more recent evidence with several cell lines has demonstrated that uridine diphosphate (UDP) is 5‐fold more potent than UDP‐glucose[281]. UDP was also shown to competitively antagonise the UDP‐glucose response at the human recombinant P2Y_14_ receptor [578].

### Further reading on P2Y receptors

Abbracchio MP et al. (2006) International Union of Pharmacology LVIII: update on the P2Y G protein‐coupled nucleotide receptors: from molecular mechanisms and pathophysiology to therapy. Pharmacol. Rev. 58: 281‐341 [PMID:16968944]

Jacobson KA et al. (2015) Nucleotides Acting at P2Y Receptors: Connecting Structure and Function. Mol. Pharmacol. 88: 220‐30 [PMID:25837834]

von Kügelgen I et al. (2016) Pharmacology and structure of P2Y receptors. Neuropharmacology 104: 50‐61 [PMID:26519900]

## Parathyroid hormone receptors

### Overview

The parathyroid hormone receptors (nomenclature as agreed by the NC‐IUPHAR Subcommittee on Parathyroid Hormone Receptors [606]) are family B G protein‐coupled receptors. The parathyroid hormone (PTH)/parathyroid hormone‐related peptide (PTHrP) receptor (PTH1 receptor) is activated by precursor‐derived peptides: PTH(PTH, P01270) (84 amino acids), and PTHrP(PTHLH, P12272) (141 amino‐acids) and related peptides (PTH‐(1‐34), PTHrP‐(1‐36)(PTHLH, P12272)). The parathyroid hormone 2 receptor (PTH2 receptor) is activated by the precursor‐derived peptide TIP39(PTH2, Q96A98) (39 amino acids). [^125^I]PTH may be used to label both PTH1 and PTH2 receptors.

**Table:** 

Nomenclature	PTH1 receptor	PTH2 receptor
HGNC, UniProt	PTH1R, Q03431	PTH2R, P49190
Potency order of endogenous ligands	PTH (PTH, P01270) = PTHrP (PTHLH, P12272)	TIP39 (PTH2, Q96A98), PTH (PTH, P01270) ≫PTHrP (PTHLH, P12272)
Agonists	teriparatide [604]	TIP39 (PTH2, Q96A98) [661, 804]
Selective agonists	PTHrP‐(1‐34) (human) [605] – Rat	–

### Comments

The parathyroid hormone type 1 receptor (PTHR) is the canonical GPCR for PTH and PTHrP. It is coupled to G_s_ and G_q_ and regulates the development of bone, heart, mammary glands and other tissues in response to PTHrP, and blood concentrations of calcium and phosphate ions, as well as vitamin D, in response to PTH. Another important action of the PTH/PTHR system is to stimulate bone formation when the hormone is intermittently administrated (daily injection). Although PTH(PTH, P01270) is an agonist at human PTH2 receptors, it fails to activate the rodent orthologues. TIP39(PTH2, Q96A98) is a weak antagonist at PTH1 receptors [925].

### Further reading on Parathyroid hormone receptors

Cheloha RW et al. (2015) PTH receptor‐1 signalling‐mechanistic insights and therapeutic prospects. Nat Rev Endocrinol [PMID:26303600]

Gardella TJ et al. (2015) International Union of Basic and Clinical Pharmacology. XCIII. The Parathyroid Hormone Receptors‐Family B G Protein‐Coupled Receptors. Pharmacol. Rev. 67: 310‐37 [PMID:25713287]

Vilardaga JP et al. (2014) Endosomal generation of cAMP in GPCR signaling. Nat. Chem. Biol. 10: 700‐6 [PMID:25271346]

## Platelet‐activating factor receptor

### Overview

Platelet‐activating factor (PAF, 1‐O‐alkyl‐2‐acetyl‐sn‐glycero‐3‐phosphocholine) is an ether phospholipid mediator associated with platelet coagulation, but also subserves inflammatory roles. The PAF receptor (provisional nomenclature recommended by NC‐IUPHAR [557]) is activated by PAF and other suggested endogenous ligands are oxidized phosphatidylcholine [1265] and lysophosphatidylcholine[1492]. It may also be activated by bacterial lipopolysaccharide [1417].

**Table:** 

Nomenclature	PAF receptor
HGNC, UniProt	PTAFR, P25105
Selective agonists	methylcarbamyl PAF
Selective antagonists	foropafant (p*K*_i_ 10.3) [774], ABT‐491 (p*K*_i_ 9.2) [30], CV‐6209 (pIC_50_ 8.1–8.3) [652, 1416], L659989 (p*K*_i_ 7.8) [851], apafant (p*K*_i_ 5.2–7.5) [1529, 1904]
Labelled ligands	[^3^ H]PAF (Agonist) [585, 1416]

### Comments

Note that a previously recommended radioligand ([^3^ H]apafant; K_d_44.6 nM) is currently unavailable.

### Further reading on Platelet‐activating factor receptor

Foord SM et al. (2005) International Union of Pharmacology. XLVI. G protein‐coupled receptor list. Pharmacol Rev 57: 279‐288 [PMID:15914470]

Ishii S et al. (2000) Platelet‐activating factor (PAF) receptor and genetically engineered PAF receptor mutant mice. Prog. Lipid Res. 39: 41‐82 [PMID:10729607]

Prescott SM et al. (2000) Platelet‐activating factor and related lipid mediators. Annu. Rev. Biochem. 69: 419‐45 [PMID:10966465]

## Prokineticin receptors

### Overview

Prokineticin receptors, PKR_1_ and PKR_2_(provisional nomenclature as recommended by NC‐IUPHAR[557]) respond to the cysteine‐rich 81‐86 amino‐acid peptides prokineticin‐1(PROK1, Q9HC23) (also known as endocrine gland‐derived vascular endothelial growth factor, mambakine) and prokineticin‐2(PROK2, Q9HC23) (protein Bv8 homologue). An orthologue of PROK1 from black mamba (Dendroaspis polylepis) venom, mamba intestinal toxin 1 (MIT1, [1749]) is a potent, non‐selective agonist at prokineticin receptors [1279], while Bv8, an orthologue of PROK2 from amphibians (Bombina sp., [1357]), is equipotent at recombinant PKR_1_ and PKR_2_[1435], and has high potency in macrophage chemotaxis assays, which are lost in PKR_1_‐null mice.

**Table:** 

Nomenclature	PKR_1_	PKR_2_
HGNC, UniProt	PROKR1, Q8TCW9	PROKR2, Q8NFJ6
Potency order of endogenous ligands	prokineticin‐2 (PROK2, Q9HC23) >prokineticin‐1 (PROK1, Q9HC23) >prokineticin‐2 *β* (PROK2) [1175, 1279, 1843]	prokineticin‐2 (PROK2, Q9HC23) >prokineticin‐1 (PROK1, Q9HC23) >prokineticin‐2 *β* (PROK2) [1175, 1279, 1843]
Endogenous agonists	prokineticin‐2 (PROK2, Q9HC23) [316, 1279], prokineticin‐1 (PROK1, Q9HC23) [316, 1279], prokineticin‐2 *β* (PROK2) [316]	prokineticin‐2 (PROK2, Q9HC23) [316, 1279], prokineticin‐1 (PROK1, Q9HC23) [316, 1279], prokineticin‐2 *β* (PROK2) [316]
Agonists	MIT1 [1279]	MIT1 [1279]
Selective agonists	IS20 [612], IS1 [612]	–
Labelled ligands	[^125^ I]BH‐MIT1 (Agonist) [1279]	[^125^ I]BH‐MIT1 (Agonist) [1279]

### Comments

Genetic mutations in PROKR1 are associated with Hirschsprung's disease [1688], while genetic mutations in PROKR2 are associated with hypogonadotropic hypogonadism with anosmia [455], hypopituitarism with pituitary stalk interruption [1649] and Hirschsprung's disease [1688].

### Further reading on Prokineticin receptors

Boulberdaa M et al. (2011) Prokineticin receptor 1 (PKR1) signalling in cardiovascular and kidney functions. Cardiovasc. Res. 92: 191‐8 [PMID:21856786]

Negri L et al. (2012) Bv8/PK2 and prokineticin receptors: a druggable pronociceptive system. Curr Opin Pharmacol 12: 62‐6 [PMID:22136937]

Negri L et al. (2007) Bv8/Prokineticin proteins and their receptors. Life Sci. 81: 1103‐16 [PMID:17881008]

Ngan ES et al. (2008) Prokineticin‐signaling pathway. Int. J. Biochem. Cell Biol. 40: 1679‐84 [PMID:18440852]

## Prolactin‐releasing peptide receptor

### Overview

The precursor (PRLH, P81277) for PrRP generates 31 and 20‐amino‐acid versions. QRFP43(QRFP, P83859) (named after a pyroglutamylated arginine‐phenylalanine‐amide peptide) is a 43 amino acid peptide derived from QRFP(P83859) and is also known as P518 or 26RFa. RFRP is an RF amide‐related peptide [794] derived from a FMRFamide‐related peptide precursor (NPVF, Q9HCQ7), which is cleaved to generate neuropeptide SF(NPFF, O15130), neuropeptide RFRP‐1(NPVF, Q9HCQ7), neuropeptide RFRP‐2(NPVF, Q9HCQ7) and neuropeptide RFRP‐3(NPVF, Q9HCQ7) (neuropeptide NPVF).

**Table:** 

Nomenclature	PrRP receptor
HGNC, UniProt	PRLHR, P49683
Potency order of endogenous ligands	PrRP‐20 (PRLH, P81277) = PrRP‐31 (PRLH, P81277) [1099]
Endogenous agonists	PrRP‐20 (PRLH, P81277) [509, 1099], PrRP‐31 (PRLH, P81277) [509, 1099]
Endogenous antagonists	neuropeptide Y (NPY, P01303) (p*K*_i_ 5.4) [1087]
Labelled ligands	[^125^ I]PrRP‐20 (human) (Agonist) [1099], [^125^ I]PrRP31 (Agonist) [501]

### Comments

The orphan receptor GPR83(Q9NYM4) shows sequence similarities with NPFF1, NPFF2, PrRP and QRFP receptors.

### Further reading on Prolactin‐releasing peptide receptor

Samson WK et al. (2006) Prolactin releasing peptide (PrRP): an endogenous regulator of cell growth. Peptides 27: 1099‐103 [PMID:16500730]

Takayanagi Y et al. (2010) Roles of prolactin‐releasing peptide and RFamide related peptides in the control of stress and food intake. FEBS J. 277: 4998‐5005 [PMID:21126313]

## Prostanoid receptors

### Overview

Prostanoid receptors (nomenclature as agreed by the NC‐IUPHAR Subcommittee on Prostanoid Receptors [2132]) are activated by the endogenous ligands prostaglandins PGD_2_, PGE_1_, PGE_2_ , PGF_2*α*_, PGH_2_, prostacyclin [PGI_2_] and thromboxane A_2_. Measurement of the potency of PGI_2_ and thromboxane A_2_ is hampered by their instability in physiological salt solution; they are often replaced by cicaprost and U46619, respectively, in receptor characterization studies.

**Table:** 

Nomenclature	DP_1_ receptor	DP_2_ receptor
HGNC, UniProt	PTGDR, Q13258	PTGDR2, Q9Y5Y4
Potency order of endogenous ligands	PGD_2_>PGE_1_≫PGE_2_>PGF_2*α*_>PGI_2_, thromboxane A_2_	PGD_2_≫PGF_2*α*_, PGE_2_>PGI_2_, thromboxane A_2_
Selective agonists	BW 245C [173, 2133, 2134], L‐644,698 [2133, 2134]	15(R)‐15‐methyl‐PGD_2_ [747, 1366, 1889]
Antagonists	–	fevipiprant (p*K*_d_ 9) [1908, 1909], ramatroban (p*K*_i_ 7.4) [1889]
Selective antagonists	laropiprant (p*K*_i_ 10.1) [1882], BWA868C (p*K*_i_ 8.6–9.3) [173, 640, 2133], ONO‐AE3‐237 (p*K*_i_ 7.7) [796, 1974, 1976]	CAY 10471 (pIC_50_ 8.9) [1684, 2003]
Labelled ligands	[^3^ H]PGD_2_ (Agonist) [2119, 2133]	[^3^ H]PGD_2_ (Agonist) [1280, 1790]

**Table:** 

Nomenclature	EP_1_ receptor	EP_2_ receptor	EP_3_ receptor	EP_4_ receptor
HGNC, UniProt	PTGER1, P34995	PTGER2, P43116	PTGER3, P43115	PTGER4, P35408
Potency order of endogenous ligands	PGE_2_>PGE_1_>PGF_2*α*_, PGI_2_>PGD_2_, thromboxane A_2_	PGE_2_ = PGE_1_>PGF_2*α*_, PGI_2_>PGD_2_, thromboxane A_2_	PGE_2_, PGE_1_>PGF_2*α*_, PGI_2_>PGD_2_, thromboxane A_2_	PGE_2_ = PGE_1_>PGF_2*α*_, PGI_2_>PGD_2_, thromboxane A_2_
Endogenous agonists	–	PGE_2_ [7, 1871, 2119]	PGE_2_ (EP_3_‐III isoform) [7]	–
Agonists	17‐phenyl‐ω‐trinor‐PGE_2_ [1783]	PGE_1_ [111]	misoprostol (methyl ester) (EP_3_‐III isoform) [7]	–
Selective agonists	ONO‐DI‐004 [1899] – Mouse	ONO‐AE1‐259 [1899] – Mouse, butaprost (free acid form) [7, 1871]	sulprostone (EP_3_‐III isoform) [7], ONO‐AE‐248 [562, 1206]	L902688 [563, 1129], ONO‐AE1‐329 [562, 563]
Antagonists	–	–	–	EP_4_A (p*K*_i_ 7.6–8.5) [1229, 2195]
Selective antagonists	ONO‐8711 (p*K*_i_ 9.2) [2079], SC‐51322 (p*K*_i_ 7.9) [7]	PF‐04418948 (PF‐04418948 has weaker affinity at the EP2‐receptor in guinea‐pigs) (p*K*_B_ 8.3) [14, 157], TG6‐129 (p*K*_B_ 8.1) [598]	L‐826266 (EP_3_‐III isoform (pK_i_=8.04 in the presence of HSA)) (p*K*_i_ 9.1) [933], ONO‐AE3‐240 (pIC_50_ 8.8) [38] – Mouse, DG‐041 (p*K*_i_ 8.4) [931]	ONO‐AE3‐208 (p*K*_i_ 8.5), GW 627368 (p*K*_i_ 7–7.1) [2119, 2120]
Labelled ligands	[^3^ H]PGE_2_ (Agonist) [7, 1783, 2119]	[^3^ H]PGE_2_ (Agonist) [7, 2119]	[^3^ H]PGE_2_ (Agonist) [7, 2119]	[^3^ H]PGE_2_ (Agonist) [7, 420, 2107, 2119]

**Table:** 

Nomenclature	FP receptor	IP receptor	TP receptor
HGNC, UniProt	PTGFR, P43088	PTGIR, P43119	TBXA2R, P21731
Potency order of endogenous ligands	PGF_2*α*_>PGD_2_>PGE_2_>PGI_2_, thromboxane A_2_	PGI_2_≫PGE_1_>PGD_2_, PGF_2*α*_>thromboxane A_2_	thromboxane A_2_ = PGH_2_≫PGD_2_, PGE_2_, PGF_2*α*_, PGI_2_
Endogenous agonists	–	PGI_2_ [1804], PGE_1_ [1277, 1873]	–
Agonists	–	iloprost [7, 2119], treprostinil [2107]	–
Selective agonists	fluprostenol [7], latanoprost (free acid form) [7]	cicaprost [7]	U46619 [7]
Antagonists	–	–	ramatroban (p*K*_i_ 8) [1944]
Selective antagonists	AS604872 (p*K*_i_ 7.5) [361]	RO1138452 (p*K*_i_ 8.7) [162], RO3244794 (pA_2_ 8.5) [162]	vapiprost (p*K*_i_ 8.3–9.4) [63, 1216], SQ‐29548 (p*K*_i_ 8.1–9.1) [7, 1907, 2119]
Labelled ligands	[^3^ H]PGF_2*α*_ (Agonist) [7, 8, 2119], [^3^ H](+)‐fluprostenol (Agonist)	[^3^ H]iloprost (Agonist) [7, 172, 2107, 2119]	[^125^ I]SAP (Antagonist) (p*K*_d_ 7.7–9.3) [1415], [^125^ I]BOP (Agonist) [1381], [^3^ H]SQ‐29548 (Antagonist) (p*K*_d_ 7.4–8.2) [7, 2119]

### Comments

Whilst cicaprost is selective for IP receptors, it does exhibit moderate agonist potency at EP_4_ receptors [7]. Apart from IP receptors, iloprost also binds to EP_1_ receptors. The IP receptor agonist treprostinil binds also to human EP_2_ and DP_1_ receptors with high affinity (pK_i_ 8.44 and 8.36, respectively) [2107]. The EP_1_ agonist 17‐phenyl‐ω‐trinor‐PGE_2_ also shows agonist activity at EP_3_ receptors. Butaprost and SC46275 may require de‐esterification within tissues to attain full agonist potency. There is evidence for subtypes of FP [1171] and TP receptors [1050, 1637]. mRNA for the EP_3_ receptor undergoes alternative splicing to produce variants which can interfere with signalling [1509] or generate complex patterns of G‐protein (G_i/o_, G_q/11_, G_s_ and G_12,13_) coupling (e.g. [1042, 1433]). The number of EP_3_ receptor (protein) variants are variable depending on species, with five in human, three in rat and three in mouse. Putative receptor(s) for prostamide F (which as yet lack molecular correlates) and which preferentially recognize PGF2‐1‐ethanolamide and its analogues (e.g. Bimatoprost) have been identified, together with moderate‐potency antagonists (e.g. AGN 211334) [2131]. The free acid form of AL‐12182, AL12180, used in in vitro studies, has a EC_50_ of 15nM which is the concentration of the compound giving half‐maximal stimulation of inositol phosphate turnover in HEK‐293 cells expressing the human FP receptor [1784]. References given alongside the TP receptor agonists I‐BOP [1295] and STA_2_[63] use human platelets as the source of TP receptors for competition radio‐ligand binding assays to determine the indicated activity values. Pharmacological evidence for a second IP receptor, denoted IP_2_, in the central nervous system [1924, 2082] and in the BEAS‐2B human airway epithelial cell line [2122] is available. This receptor is selectively activated by 15R‐17,18,19, 20‐tetranor‐16‐m‐tolyl‐isocarbacyclin (15R‐TIC) and 15R‐Deoxy 17,18,19,20‐tetranor‐16‐m‐tolyl‐isocarbacyclin (15‐deoxy‐TIC). However, molecular biological evidence for an IP_2_ subtype is currently lacking.

### Further reading on Prostanoid receptors

Woodward DF et al. (2011) International union of basic and clinical pharmacology. LXXXIII: classification of prostanoid receptors, updating 15 years of progress. Pharmacol. Rev. 63: 471‐538 [PMID:21752876]

## Proteinase‐activated receptors

### Overview

Proteinase‐activated receptors (PARs, nomenclature as agreed by the NC‐IUPHAR Subcommittee on Proteinase‐activated Receptors [809]) are unique members of the GPCR superfamily activated by proteolytic cleavage of their amino terminal exodomains. Agonist proteinase‐induced hydrolysis unmasks a tethered ligand (TL) at the exposed amino terminus, which acts intramolecularly at the binding site in the body of the receptor to effect transmembrane signalling. TL sequences at human PAR1‐4 are SFLLRN‐NH_2_, SLIGKV‐NH_2_, TFRGAP‐NH_2_ and GYPGQV‐NH_2_, respectively. With the exception of PAR3, these synthetic peptide sequences (as carboxyl terminal amides) are able to act as agonists at their respective receptors. Several proteinases, including neutrophil elastase, cathepsin G and chymotrypsin can have inhibitory effects at PAR1 and PAR2 such that they cleave the exodomain of the receptor without inducing activation of G*α*q‐coupled calcium signalling, thereby preventing activation by activating proteinases but not by agonist peptides. Neutrophil elastase (NE) cleavage of PAR1 and PAR2 can however activate MAP kinase signaling by exposing a TL that is different from the one revealed by trypsin [1624]. PAR2 ectivation by NE regulates inflammation and pain responses [1397, 2217] and triggers mucin secretion from airway epithelial cells [2220].

**Table:** 

Nomenclature	PAR1	PAR2	PAR3	PAR4
HGNC, UniProt	F2R, P25116	F2RL1, P55085	F2RL2, O00254	F2RL3, Q96RI0
Agonist proteases	thrombin (F2, P00734), activated protein C (PROC, P04070), matrix metalloproteinase 1 (MMP1, P45452), matrix metalloproteinase 13 (MMP13, P45452) [73]	Trypsin, tryptase, TF/VIIa, Xa	thrombin (F2, P00734)	thrombin (F2, P00734), trypsin, cathepsin G (CTSG, P08311)
Agonists	F16357	–	–	–
Selective agonists	TFLLR‐NH_2_ [355]	AC264613 [1767], AY77 [2178], AC‐55541 [1767], GB110 [104], 2‐furoyl‐LIGRLO‐amide [1305], SLIGKV‐NH_2_ [1134], SLIGRL‐NH_2_ [1134]	–	AYPGKF‐NH2, GYPGKF‐NH2, GYPGQV‐NH_2_
Selective antagonists	vorapaxar (p*K*_i_ 8.1) [295], atopaxar (pIC_50_ 7.7) [1024], RWJ‐56110 (pIC_50_ 6.4) [49]	GB88 (pIC_50_ 5.7) [1886], P2pal18s [1776]	–	YD‐3 (pIC_50_ 6.9) [2091], ML354 (pIC_50_ 6.8) [2091]
Labelled ligands	[^3^ H]haTRAP (Agonist) [17]	2‐furoyl‐LIGRL[N‐(Alexa Fluor 594)‐O]‐NH_2_ (Agonist) [810], 2‐furoyl‐LIGRL[N[^3^ H]propionyl]‐O‐NH_2_ (Agonist) [810], [^3^ H]2‐furoyl‐LIGRL‐NH_2_ (Selective Agonist) [946], trans‐cinnamoyl‐LIGRLO [N‐[ ^3^H]propionyl]‐NH_2_ (Agonist) [28]	–	–
Comments	TFLLR‐NH_2_ is selective relative to the PAR_2_ receptor [159, 958].	2‐Furoyl‐LIGRLO‐NH_2_ activity was measured via calcium mobilisation in HEK 293 cells which constitutively coexpress human PAR_1_ and PAR_2_.	–	–

### Comments

Endogenous serine proteases (EC 3.4.21.) active at the proteinase‐activated receptors include: thrombin(F2, P00734), generated by the action of Factor X (F10, P00742) on liver‐derived prothrombin (F2, P00734); trypsin, generated by the action of enterokinase (TMPRSS15, P98073) on pancreatic‐derived trypsinogen (PRSS1, P07477); tryptase, a family of enzymes (*α*/*β*1 TPSAB1, Q15661 ; *γ*1 TPSG1, Q9NRR2; *δ*1 TPSD1, Q9BZJ3) secreted from mast cells; cathepsin G (CTSG, P08311) generated from leukocytes; liver‐derived protein C (PROC, P04070) generated in plasma by thrombin(F2, P00734) and matrix metalloproteinase 1 (MMP1, P45452).

### Further reading on Proteinase‐activated receptors

Adams MN et al. (2011) Structure, function and pathophysiology of protease activated receptors. Pharmacol. Ther. 130: 248‐82 [PMID:21277892]

Canto I et al. (2012) Allosteric modulation of protease‐activated receptor signaling. Mini Rev Med Chem 12: 804‐11 [PMID:22681248]

García PS et al. (2010) The role of thrombin and protease‐activated receptors in pain mechanisms. Thromb. Haemost. 103: 1145‐51 [PMID:20431855]

Hollenberg MD et al. (2002) International Union of Pharmacology. XXVIII. Proteinase‐activated receptors. Pharmacol. Rev. 54: 203‐17 [PMID:12037136]

Ramachandran R et al. (2012) Targeting proteinase‐activated receptors: therapeutic potential and challenges. Nat Rev Drug Discov 11: 69‐86 [PMID:22212680]

Soh UJ et al. (2010) Signal transduction by protease‐activated receptors. Br. J. Pharmacol. 160: 191‐203 [PMID:20423334]

## QRFP receptor

### Overview

The human gene encoding the QRFP receptor (QRFPR, also known as the peptide P518 receptor), previously designated as an orphan GPCR receptor was identified in 2001 by Lee et al. from a hypothalamus cDNA library [1131]. However, the reported cDNA (AF411117) is a chimera with bases 1‐127 derived from chromosome 1 and bases 155‐1368 derived from chromosome 4. When corrected, QRFPR (also referred to as SP9155 or AQ27) encodes a 431 amino acid protein that shares sequence similarities in the transmembrane spanning regions with other peptide receptors. These include neuropeptide FF2 (38%), neuropeptide Y_2_ (37%) and galanin Gal_1_(35%) receptors.

**Table:** 

Nomenclature	QRFP receptor
HGNC, UniProt	QRFPR, Q96P65
Endogenous agonists	QRFP43 (QRFP, P83859) [311, 587, 1923] – Rat, QRFP26 (QRFP) [311, 910]
Agonists	LV‐2172 [1448]
Selective antagonists	compound 25e (pIC_50_ 7.3) [628, 629]
Labelled ligands	[^125^ I]QRFP43 (human) (Agonist) [587, 1063, 1923]

### Comments

The orphan receptor GPR83(9NYM4) shows sequence similarities with the QRFP receptor, as well as with the NPFF1, NPFF2, and PrRP receptors.

### Further reading on QRFP receptor

Fukusumi S et al. (2006) Recent advances in mammalian RFamide peptides: the discovery and functional analyses of PrRP, RFRPs and QRFP. Peptides 27: 1073‐86 [PMID:16500002]

## Relaxin family peptide receptors

### Overview

Relaxin family peptide receptors (RXFP, nomenclature as agreed by the NC‐IUPHAR Subcommittee on Relaxin family peptide receptors [112, 713]) may be divided into two pairs, RXFP1/2 and RXFP3/4. Endogenous agonists at these receptors are a number of heterodimeric peptide hormones structurally related to insulin: relaxin‐1(RLN1, P04808), relaxin(RLN2, P04090), relaxin‐3(RLN3, Q8WXF3) (also known as INSL7), insulin‐like peptide 3 (INSL3(INSL3, P51460)) and INSL5(INSL5, Q9Y5Q6).

Species homologues of relaxin have distinct pharmacology – relaxin(RLN2, P04090) interacts with RXFP1, RXFP2 and RXFP3, whereas mouse and rat relaxin selectively bind to and activate RXFP1 [1755] and porcine relaxin may have a higher efficacy than human relaxin(RLN2, P04090) [714]. Relaxin‐3(RLN3, Q8WXF3) has differential affinity for RXFP2 between species; mouse and rat RXFP2 have a higher affinity for relaxin‐3(RLN3, Q8WXF3) [1754]. At least two binding sites have been identified on RXFP1 and RXFP2: a high‐affinity site in the leucine‐rich repeat region of the ectodomain and a somewhat lower‐affinity site located in the surface loops of the transmembrane domain [714, 1885]. The unique N‐terminal LDLa module of RXFP1 and RXFP2 is essential for receptor signalling [1756].

**Table:** 

Nomenclature	RXFP1	RXFP2	RXFP3	RXFP4
HGNC, UniProt	RXFP1, Q9HBX9	RXFP2, Q8WXD0	RXFP3, Q9NSD7	RXFP4, Q8TDU9
Potency order of endogenous ligands	relaxin (RLN2, P04090) = relaxin‐1 (RLN1, P04808) >relaxin‐3 (RLN3, Q8WXF3) [1885]	INSL3 (INSL3, P51460) >relaxin (RLN2, P04090) ≫relaxin‐3 (RLN3, Q8WXF3) [1072, 1885]	relaxin‐3 (RLN3, Q8WXF3) >relaxin‐3 (B chain) (RLN3, Q8WXF3) >relaxin (RLN2, P04090) [1186]	INSL5 (INSL5, Q9Y5Q6) = relaxin‐3 (RLN3, Q8WXF3) >relaxin‐3 (B chain) (RLN3, Q8WXF3) [1184, 1185]
Endogenous antagonists	–	–	INSL5 (INSL5, Q9Y5Q6) (p*K*_i_ 7) [2223]	–
Antagonists	B‐R13/17K H2 relaxin (pEC_50_ 5.7–6.7) [827, 1446]	–	R3(BΔ23‐27)R/I5 chimeric peptide (pIC_50_ 9.2) [1064]	R3(BΔ23‐27)R/I5 chimeric peptide (pIC_50_ 8–8.6) [749, 1064]
Selective antagonists	–	A(9‐26)INSL3 (p*K*_i_ 9.1) [826], A(10‐24)INSL3 (p*K*_i_ 8.7) [826], A(C10/15S)INSL3 (p*K*_i_ 8.6) [2210], INSL3 B chain dimer analogue 8 (p*K*_i_ 8.5) [1781], A(Δ10/15C)INSL3 (p*K*_i_ 8.3) [2210], cyclic INSL3 B‐chain analogue 6 (p*K*_i_ 6.7) [1779], INSL3 B‐chain analogue (p*K*_i_ 5.1) [434], (des 1‐8) A‐chain INSL3 analogue [262]	minimised relaxin‐3 analogue 3 (p*K*_i_ 7.6) [1777], R3‐B1‐22R (p*K*_i_ 7.4) [749]	minimised relaxin‐3 analogue 3 (pIC_50_ 6.6) [1777]
Allosteric modulators	ML290 (Agonist) (pEC_50_ 7) [2146, 2149]	–	–	–
Labelled ligands	[^33^ P]relaxin (human) (Agonist) [714, 1885], europium‐labelled relaxin (Agonist) [1778], [^125^ I]relaxin (human) (Agonist)	[^125^ I]INSL3 (human) (Agonist) [1395], [^33^ P]relaxin (human) (Agonist) [714, 1885]	[^125^ I]relaxin‐3 (human) (Agonist) [1186], [^125^ I]relaxin‐3‐B/INSL5 A chimera (Agonist) [1184], europium‐labelled relaxin‐3‐B/INSL5 A chimera (Agonist) [749]	[^125^ I]relaxin‐3 (human) (Agonist) [1185], [^125^ I]relaxin‐3‐B/INSL5 A chimera (Agonist) [1184], europium‐labelled mouse INSL5 (Agonist) [126], europium‐labelled relaxin‐3‐B/INSL5 A chimera (Agonist) [749], europium‐labelled INSL5 (p*K*_d_ 8.3) [749]
Comments	–	europium‐labelled INSL3 is a fluorescent ligand for this receptor (K_d_=1nM) [1780].	–	–

### Comments

Relaxin is the cognate peptide ligand for RXFP1 and is in extended Phase III clinical trials for the treatment of acute heart failure [1322]. Relaxin has vasodilatory, anti‐fibrotic, angiogenic, anti‐apoptotic and anti‐inflammatory effects. Small molecule allosteric agonists such as ML290 have been developed [1787, 2149]. The antifibrotic actions of relaxin are dependent on the angiotensin receptor AT_2_[344]. RXFP2 and its cognate ligand INSL3 have a more specialized role with mutations reported in patients with cryptorchidism [538]. cAMP elevation is the major signalling pathway for RXFP1 and RXFP2 [834, 835], but RXFP1 also activates MAP kinases, nitric oxide signalling, tyrosine kinase phosphorylation and relaxin can interact with glucocorticoid receptors [716]. RXFP1 signalling involves lipid rafts, residues in the C‐terminus of the receptor and activation of phosphatidylinositol‐3‐kinase [717] and pre‐assembled protein complexes [715]. Receptor expression profiles suggest that RXFP3 is a brain neuropeptide receptor and RXFP4 a gut hormone receptor with the relaxin‐3/RXFP3 system modulating feeding [596, 597, 749, 1777, 1830], anxiety [1694, 2204], and reward and motivated goal‐directed behaviours [821, 1694, 2055]. Relaxin‐3(RLN3, Q8WXF3) is an agonist at RXFP3 and RXFP4 whereas INSL5(INSL5, Q9Y5Q6) is an agonist at RXFP4 and a weak antagonist at RXFP3. Unlike RXFP1 and RXFP2, both RXFP3 and RXFP4 are encoded by a single exon. INSL5 is secreted from enteroendocrine L cells and the INSL5/RXFP4 system controls food intake and glucose homeostasis [685]. RXFP3 and RXFP4 couple to G_i/o_ and inhibit adenylyl cyclase [1186, 2014], and also cause Erk1/2 phosphorylation [2014]. RXFP4 also causes phosphorylation of p38MAPK, Akt and S6RP [51]. There is evidence that at RXFP3, relaxin(RLN2, P04090) is a biased ligand compared to the cognate ligand relaxin‐3.

### Further reading on Relaxin family peptide receptors

Bathgate RA et al. (2013) Relaxin family peptides and their receptors. Physiol. Rev. 93: 405‐80 [PMID:23303914]

Du XJ et al. (2010) Cardiovascular effects of relaxin: from basic science to clinical therapy. Nat Rev Cardiol 7: 48‐58 [PMID:19935741]

Halls ML et al. (2015) International Union of Basic and Clinical Pharmacology. XCV. Recent advances in the understanding of the pharmacology and biological roles of relaxin family peptide receptors 1‐4, the receptors for relaxin family peptides. Pharmacol. Rev. 67: 389‐440 [PMID:25761609]

Ivell R et al. (2011) Relaxin family peptides in the male reproductive system–a critical appraisal. Mol. Hum. Reprod. 17: 71‐84 [PMID:20952422]

## Somatostatin receptors

### Overview

Somatostatin (somatotropin release inhibiting factor) is an abundant neuropeptide, which acts on five subtypes of somatostatin receptor (SST_1_‐SST_5_; nomenclature as agreed by the NC‐IUPHAR Subcommittee on Somatostatin Receptors [829]). Activation of these receptors produces a wide range of physiological effects throughout the body including the inhibition of secretion of many hormones. Endogenous ligands for these receptors are somatostatin‐14 (SRIF‐14(SST, P61278)) and somatostatin‐28 (SRIF‐28(SST, P61278)). Cortistatin‐14{Mouse, Rat} has also been suggested to be an endogenous ligand for somatostatin receptors [427].

**Table:** 

Nomenclature	SST1 receptor	SST2 receptor	SST3 receptor	SST4 receptor	SST5 receptor
HGNC, UniProt	SSTR1, P30872	SSTR2, P30874	SSTR3, P32745	SSTR4, P31391	SSTR5, P35346
Agonists	pasireotide [1740]	pasireotide [1740]	pasireotide [1740], vapreotide [238, 1540, 1807]	NNC269100 [1197]	pasireotide [1740]
Selective agonists	L‐797,591 [1669], Des‐Ala ^1,2,5^‐[D‐Trp^8^,IAmp^9^]SRIF [512]	L‐054,522 [2172], BIM 23027 [283], octreotide [238, 1540, 1805, 1806, 1807, 2172]	L‐796,778 [1669]	L‐803,087 [1669]	BIM 23052 [1325, 1805, 1806, 1807], L‐817,818 [1669]
Selective antagonists	SRA880 (p*K*_d_ 8–8.1) [831]	[D‐Tyr ^8^]CYN 154806 (p*K*_d_ 8.1–8.9) [1478]	NVP ACQ090 (p*K*_i_ 7.9) [832]	–	–
Labelled ligands	–	[^125^I]Tyr^3^ SMS 201‐995 (Agonist) [1805, 1806], [^125^ I]BIM23027 (Agonist) [811] – Rat	–	–	[^125^I]Tyr^3^ SMS 201‐995 (Agonist) [1805, 1806]

### Comments

[^125^I]Tyr^11^ ‐SRIF‐14, [^125^ I]LTT‐SRIF‐28, [^125^ I]CGP 23996 and [^125^I]Tyr^10^ ‐CST14 may be used to label somatostatin receptors nonselectively. A number of nonpeptide subtype‐selective agonists have been synthesised [1669]. Octreotide and lanreotide are being used in the treatment of SST_2_‐expressing neuroendocrine tumors and pasireotide for SST_5_‐expressing neuroendocrine tumors. A novel peptide somatostatin analogue, veldoreotide (somatoprim), has affinity for SST_2_, SST_4_ and SST_5_ receptors and is a potent inhibitor of GH secretion [1586, 1793].

### Further reading on Somatostatin receptors

Colao A et al. (2011) Resistance to somatostatin analogs in acromegaly. Endocr. Rev. 32: 247‐71 [PMID:21123741]

Hoyer D et al. (2000) Somatostatin receptors. In The IUPHAR Compendium of Receptor Characterization and Classification, 2nd edn. Edited by Watson SP, Girdlestone D: IUPHAR Media: 354‐364

Schulz S et al. (2014) Fine‐tuning somatostatin receptor signalling by agonist‐selective phosphorylation and dephosphorylation: IUPHAR Review 5. Br. J. Pharmacol. 171: 1591‐9 [PMID:24328848]

Weckbecker G et al. (2003) Opportunities in somatostatin research: biological, chemical and therapeutic aspects. Nat Rev Drug Discov 2: 999‐1017 [PMID:14654798]

## Succinate receptor

### Overview

Nomenclature as recommended by NC‐IUPHAR[414]. The Succinate receptor has been identified as being activated by physiological levels of the Krebs' cycle intermediate succinate and other dicarboxylic acids such as maleate in 2004. Since its pairing with its endogenous ligand, the receptor has been the focus of intensive research and its role has been evidenced in various (patho)physiological processes such as regulation of renin production, retinal angiogenesis or immune response.

**Table:** 

Nomenclature	succinate receptor
HGNC, UniProt	SUCNR1, Q9BXA5
Endogenous agonists	succinic acid [762, 1854]

### Comments

In humans, there is the possibility of two open‐reading frames (ORFs) for *SUCNR1*, allowing the generation of 330 or 334 amino acid proteins Wittenberger et al.[2127] noted that the 330‐AA protein was more likely to be expressed given the Kozak sequence surrounding the second ATG. Some databases report SUCNR1 as being 334‐AA long.

### Further reading on Succinate receptor

Ariza AC et al. (2012) The succinate receptor as a novel therapeutic target for oxidative and metabolic stress‐related conditions. Front Endocrinol (Lausanne) 3: 22. [PMID:22649411]

de Castro Fonseca M et al. (2016) GPR91: expanding the frontiers of Krebs cycle intermediates. Cell Commun. Signal 14: 3 [PMID:26759054]

Gilissen J et al. (2016) Insight into SUCNR1 (GPR91) structure and function. Pharmacol. Ther. 159: 56‐65 [PMID:26808164]

Peti‐Peterdi J. (2010) High glucose and renin release: the role of succinate and GPR91. Kidney Int. 78 (12): 1214‐7. [PMID:20861827]

## Tachykinin receptors

### Overview

Tachykinin receptors (provisional nomenclature as recommended by NC‐IUPHAR[557]) are activated by the endogenous peptides substance P(TAC1, P20366) (SP), neurokinin A (TAC1, P20366) (NKA; previously known as substance K, neurokinin *α*, neuromedin L), neurokinin B (TAC3, Q9UHF0) (NKB; previously known as neurokinin *β*, neuromedin K), neuropeptide K(TAC1, P20366) and neuropeptide *γ*(TAC1, P20366) (N‐terminally extended forms of neurokinin A). The neurokinins (A and B) are mammalian members of the tachykinin family, which includes peptides of mammalian and nonmammalian origin containing the consensus sequence: Phe‐x‐Gly‐Leu‐Met. Marked species differences in in vitro pharmacology exist for all three receptors, in the context of nonpeptide ligands. Antagonists such as aprepitant and fosaprepitant were approved by FDA and EMA, in combination with other antiemetic agents, for the prevention of nausea and vomiting associated with emetogenic cancer chemotherapy.

**Table:** 

Nomenclature	NK_1_ receptor	NK_2_ receptor	NK_3_ receptor
HGNC, UniProt	TACR1, P25103	TACR2, P21452	TACR3, P29371
Potency order of endogenous ligands	substance P (TAC1, P20366) >neurokinin A (TAC1, P20366) >neurokinin B (TAC3, Q9UHF0)	neurokinin A (TAC1, P20366) >neurokinin B (TAC3, Q9UHF0) ≫substance P (TAC1, P20366)	neurokinin B (TAC3, Q9UHF0) >neurokinin A (TAC1, P20366) >substance P (TAC1, P20366)
Agonists	substance P‐OMe [1960]	–	–
Selective agonists	[Sar ^9^,Met(O_2_)^11^]SP [1960], septide [130, 746], [Pro ^9^]SP [1975] – Rat	[Lys ^5^,Me‐Leu^9^,Nle^10^]NKA‐(4‐10) [1292] – Rat, GR64349 [432] – Rat, [*β*Ala^8^ ]neurokinin A‐(4‐10) [505]	[Phe(Me)^7^ ]neurokinin B [1717, 1718], senktide [1717, 1718, 1960]
Selective antagonists	aprepitant (p*K*_i_ 10.1) [709, 710], CP 99994 (p*K*_i_ 9.3–9.7) [53, 1718], RP67580 (pIC_50_ 7.7) [555]	GR94800 (p*K*_i_ 9.8) [206], saredutant (p*K*_i_ 9.4–9.7) [53, 505, 1718], GR 159897 (p*K*_d_ 7.8–9.5) [137, 505, 1837], MEN10627 (p*K*_i_ 9.2) [638], nepadutant (p*K*_i_ 8.5–8.7) [284, 358]	osanetant (p*K*_i_ 8.4–9.7) [53, 116, 357, 504, 941, 1518, 1717, 1718, 1960], talnetant (p*K*_i_ 7.4–9) [133, 639, 1717, 1718], PD157672 (pIC_50_ 7.8–7.9) [168, 1960]
Labelled ligands	[^125^ I]L703,606 (Antagonist) (p*K*_d_ 9.5) [566], [^125^ I]BH‐[Sar ^9^,Met(O_2_)^11^]SP (Agonist) [1979] – Rat, [^3^ H]SP (human, mouse, rat) (Agonist) [84], [^125^ I]SP (human, mouse, rat) (Agonist), [^18^ F]SPA‐RQ (Antagonist) [332]	[^3^ H]saredutant (Antagonist) (p*K*_d_ 9.7) [683] – Rat, [^125^ I]NKA (human, mouse, rat) (Agonist) [2077], [^3^ H]GR100679 (Antagonist) (p*K*_d_ 9.2) [705]	[^3^ H]osanetant (Antagonist) (p*K*_d_ 9.9), [^3^ H]senktide (Agonist) [693] – Guinea pig, [^125^ I][MePhe ^7^]NKB (Agonist)

### Comments

The NK_1_ receptor has also been described to couple to G proteins other than G_q/11_[1680]. The hexapeptide agonist septide appears to bind to an overlapping but non‐identical site to substance P(TAC1, P20366) on the NK_1_ receptor. There are suggestions for additional subtypes of tachykinin receptor; an orphan receptor (SwissProt P30098) with structural similarities to the NK_3_ receptor was found to respond to NKB when expressed in Xenopus oocytes or Chinese hamster ovary cells [459, 1049].

### Further reading on Tachykinin receptors

Douglas SD et al. (2011) Neurokinin‐1 receptor: functional significance in the immune system in reference to selected infections and inflammation. Ann. N. Y. Acad. Sci. 1217: 83‐95 [PMID:21091716]

Foord SM et al. (2005) International Union of Pharmacology. XLVI. G protein‐coupled receptor list. Pharmacol Rev 57: 279‐288 [PMID:15914470]

Jones S et al. (2008) The neurokinin 1 receptor: a potential new target for anti‐platelet therapy? Curr Opin Pharmacol 8: 114‐9 [PMID:18296119]

Rance NE et al. (2010) Neurokinin B and the hypothalamic regulation of reproduction. Brain Res. 1364: 116‐28 [PMID:20800582]

Rojas C et al. (2012) Pharmacological mechanisms of 5‐HT_3 and tachykinin NK_1 receptor antagonism to prevent chemotherapy‐induced nausea and vomiting. Eur. J. Pharmacol. 684: 1‐7 [PMID:22425650]

Steinhoff MS et al. (2014) Tachykinins and their receptors: contributions to physiological control and the mechanisms of disease. Physiol. Rev. 94: 265‐301 [PMID:24382888]

## Thyrotropin‐releasing hormone receptors

### Overview

Thyrotropin‐releasing hormone (TRH) receptors (provisional nomenclature as recommended by NC‐IUPHAR[557]) are activated by the endogenous tripeptide TRH(TRH, P20396) (pGlu‐His‐ProNH2). TRH(TRH, P20396) and TRH analogues fail to distinguish TRH_1_ and TRH_2_ receptors [1896]. [^3^ H]TRH (human, mouse, rat) is able to label both TRH_1_ and TRH_2_ receptors with K_d_ values of 13 and 9 nM respectively. Synthesis and biology of ring‐modified L‐Histidine containing TRH analogues has been reported [1316].

**Table:** 

Nomenclature	TRH_1_ receptor	TRH_2_ receptor
HGNC, UniProt	TRHR, P34981	–
Antagonists	diazepam (p*K*_i_ 5.2) [471] – Rat	–
Selective antagonists	midazolam (p*K*_i_ 5.5) [471] – Rat, chlordiazepoxide (p*K*_i_ 4.8) [471] – Rat, chlordiazepoxide (p*K*_i_ 4.7) [1878] – Mouse	–
Comments	–	A class A G protein‐coupled receptor: not present in man

### Further reading on Thyrotropin‐releasing hormone receptors

Bílek R et al. (2011) TRH‐like peptides. Physiol Res 60: 207‐15 [PMID:21114375]

Foord SM et al. (2005) International Union of Pharmacology. XLVI. G protein‐coupled receptor list. Pharmacol Rev 57: 279‐288 [PMID:15914470]

Nillni EA. (2010) Regulation of the hypothalamic thyrotropin releasing hormone (TRH) neuron by neuronal and peripheral inputs. Front Neuroendocrinol 31: 134‐56 [PMID:20074584]

## Trace amine receptor

### Overview

Trace amine‐associated receptors were discovered from a search for novel 5‐HT receptors [189], where 15 mammalian orthologues were identified and divided into two families. The TA_1_ receptor (nomenclature as agreed by the NC‐IUPHAR Subcommittee for the Trace amine receptor [1244]) has affinity for the endogenous trace amines tyramine, *β* ‐phenylethylamine and octopamine in addition to the classical amine dopamine[189]. Emerging evidence suggests that TA_1_ is a modulator of monoaminergic activity in the brain [2151] with TA_1_ and dopamine D_2_ receptors shown to form constitutive heterodimers when co‐expressed [519]. In addition to trace amines, receptors can be activated by amphetamine‐like psychostimulants, and endogenous thyronamines.

**Table:** 

Nomenclature	TA_1_ receptor
HGNC, UniProt	TAAR1, Q96RJ0
Potency order of endogenous ligands	tyramine>*β* ‐phenylethylamine>octopamine = dopamine [189]
Agonists	RO5166017 [1648]
Antagonists	EPPTB (Inverse agonist) (pIC_50_ 5.1) [205]
Labelled ligands	[^3^ H]tyramine (Agonist) [189]

### Comments

In addition to TA_1_, in man there are up to 5 functional TAAR genes (TAAR2,5,6,8,9). See [189] for detailed discussion. The product of the gene TAAR2 (also known as GPR58) appears to respond to *β* ‐phenylethylamine>tyramine and to couple through G_s_[189]. TAAR3, in some individuals, and TAAR4 are pseudogenes in man, although functional in rodents. The signalling characteristics and pharmacology of TAAR_5_(PNR, Putative Neurotransmitter Receptor: TAAR5, O14804), TAAR_6_(Trace amine receptor 4, TaR‐4: TAAR6, 96RI8), TAAR_8_(Trace amine receptor 5, GPR102: TAAR8, Q969N4) and TAAR_9_(trace amine associated receptor 9: TAAR9, 96RI9) are lacking. The thyronamines, endogenous derivatives of thyroid hormone, have affinity for rodent cloned trace amine receptors, including TA_1_[1728]. An antagonist EPPTB has recently been described with a pK_i_ of 9.1 at the mouse TA_1_ but >5.3 for human TA_1_[1863].

### Further reading on Trace amine receptor

Maguire JJ et al. (2009) International Union of Pharmacology. LXXII. Recommendations for trace amine receptor nomenclature. Pharmacol. Rev. 61: 1‐8 [PMID:19325074]

Pei Y et al. (2016) Trace Amines and the Trace Amine‐Associated Receptor 1: Pharmacology, Neurochemistry, and Clinical Implications. Front Neurosci 10: 148 [PMID:27092049]

## Urotensin receptor

### Overview

The urotensin‐II (U‐II) receptor (UT, nomenclature as agreed by the NC‐IUPHAR Subcommittee on the Urotensin receptor [466, 557, 2032]) is activated by the endogenous dodecapeptide urotensin‐II(UTS2, O95399), originally isolated from the urophysis, the endocrine organ of the caudal neurosecretory system of teleost fish [138]. Several structural forms of U‐II exist in fish and amphibians. The goby orthologue was used to identify U‐II as the cognate ligand for the predicted receptor encoded by the rat gene gpr14 [389, 1195, 1379, 1476]. Human urotensin‐II(UTS2, O95399), an 11‐amino‐acid peptide [389], retains the cyclohexapeptide sequence of goby U‐II that is thought to be important in ligand binding [224, 1003]. This sequence is also conserved in the deduced amino‐acid sequence of rat urotensin‐II {Rat} (14 amino‐acids) and mouse urotensin‐II{Mouse} (14 amino‐acids), although the N‐terminal is more divergent from the human sequence [388]. A second endogenous ligand for the UT has been discovered in rat [1890]. This is the urotensin II‐related peptide (UTS2B, Q765I0), an octapeptide that is derived from a different gene, but shares the C‐terminal sequence (CFWKYCV) common to U‐II from other species. Identical sequences to rat urotensin II‐related peptide (UTS2B, Q765I0) are predicted for the mature mouse and human peptides [472]. UT exhibits relatively high sequence identity with somatostatin, opioid and galanin receptors [2032].

**Table:** 

Nomenclature	UT receptor
HGNC, UniProt	UTS2R, Q9UKP6
Endogenous agonists	urotensin II‐related peptide (UTS2B, Q765I0) [472, 1243], urotensin‐II (UTS2, O95399) [467, 503, 681]
Selective agonists	[Pen^5^]U‐(4‐11) (human) [681], U‐II‐(4‐11) (human) [681], [3‐iodo‐Tyr ^6^]U‐II‐(4‐11) (human) [1084], Urolinin [95], FL104 [1139, 1141], AC‐7954 [398, 1140]
Selective antagonists	JNJ‐39319202 (p*K*_i_ 8.4) [1106], urantide (p*K*_i_ 8.3) [1536], SB‐706375 (p*K*_i_ 8) [467], [Orn ^5^]URP (p*K*_i_ 7.2) [445] – Rat, palosuran (pIC_50_ 7.1) [366], SB‐436811 (p*K*_i_ 6.7) [912] – Rat, SB‐611812 (p*K*_i_ 6.6) [1622], S6716 (Inverse agonist) (pIC_50_ 6.4) [554], [Cha^6^]U‐II‐(4‐11) (p*K*_i_ 6.4) [312] – Rat
Labelled ligands	[^125^ I]U‐II (human) (Agonist) [42, 198, 312, 1243], [^125^ I]N‐biotin‐[Ahx ^0^, Bpa^3^]U‐II (human) [454]

### Comments

In the human vasculature, human urotensin‐II(UTS2, O95399) elicits both vasoconstrictor (pD_2_ 9.3‐10.1, [1243]) and vasodilator (pIC_50_ 10.3‐10.4, [1872]) responses.

### Further reading on Urotensin receptor

Foord SM et al. (2005) International Union of Pharmacology. XLVI. G protein‐coupled receptor list. Pharmacol Rev 57: 279‐288 [PMID:15914470]

Hunt BD et al. (2010) A rat brain atlas of urotensin‐II receptor expression and a review of central urotensin‐II effects. Naunyn Schmiedebergs Arch. Pharmacol. 382: 1‐31 [PMID:20422157]

Maryanoff BE et al. (2010) Urotensin‐II receptor modulators as potential drugs. J. Med. Chem. 53: 2695‐708 [PMID:20043680]

Ross B et al. (2010) Role of urotensin II in health and disease. Am. J. Physiol. Regul. Integr. Comp. Physiol. 298: R1156‐72 [PMID:20421634]

## Vasopressin and oxytocin receptors

### Overview

Vasopressin (AVP) and oxytocin (OT) receptors (nomenclature as recommended by NC‐IUPHAR[557]) are activated by the endogenous cyclic nonapeptides vasopressin(AVP, P01185) and oxytocin(OXT, P01178). These peptides are derived from precursors which also produce neurophysins (neurophysin I for oxytocin; neurophysin II for vasopressin).

**Table:** 

Nomenclature	V_1A_ receptor	V_1B_ receptor
HGNC, UniProt	AVPR1A, P37288	AVPR1B, P47901
Potency order of endogenous ligands	vasopressin (AVP, P01185) >oxytocin (OXT, P01178)	vasopressin (AVP, P01185) >oxytocin (OXT, P01178)
Endogenous agonists	vasopressin (AVP, P01185) [24, 326, 383, 439, 1418, 1571, 1702, 1913, 1914, 1945, 1946, 2162]	vasopressin (AVP, P01185) [24, 326, 439, 682, 1418, 1702, 1913, 1914, 1946, 2162]
Selective agonists	F180 [50, 383]	d[Leu ^4^]LVP [1553], d[Cha ^4^]AVP [439, 682]
Antagonists	conivaptan (p*K*_i_ 8.2–8.4) [1913, 1914]	nelivaptan (p*K*_i_ 8.4–9.3) [678, 682, 1773]
Selective antagonists	relcovaptan (p*K*_i_ 8.1–9.3) [24, 383, 682, 1571, 1771, 1913, 1945, 1946, 1986], d(CH_2_)_5_[Tyr(Me)^2^,Arg^8^]VP (p*K*_i_ 9)	–
Labelled ligands	[^125^ I]OH‐LVA (Antagonist) (p*K*_d_ 10.3–10.4) [334, 383, 1571], [^3^ H]AVP (human, mouse, rat) (Agonist) [214, 334, 383, 384, 1418, 1571, 1702, 1913, 1914, 1945, 1946, 1986, 2162], [^3^ H]d(CH_2_)_5_[Tyr(Me)^2^]AVP (Antagonist) (p*K*_d_ 9)	[^3^ H]AVP (human, mouse, rat) (Agonist) [214, 334, 383, 384, 1418, 1571, 1702, 1913, 1914, 1945, 1946, 1986, 2162]

**Table:** 

Nomenclature	V_2_ receptor	OT receptor
HGNC, UniProt	AVPR2, P30518	OXTR, P30559
Potency order of endogenous ligands	vasopressin (AVP, P01185) >oxytocin (OXT, P01178)	oxytocin (OXT, P01178) >vasopressin (AVP, P01185)
Endogenous agonists	vasopressin (AVP, P01185) [24, 326, 334, 439, 1418, 1702, 1771, 1913, 1914, 1946, 2162]	oxytocin (OXT, P01178) [24, 334, 335, 360, 682, 895]
Selective agonists	VNA932 [527], OPC‐51803 [1418], d[Val ^4^,DArg^8^]VP	[Thr ^4^,Gly^7^]OT [335, 500, 895]
Antagonists	–	L‐371,257 (p*K*_i_ 8.8) [682]
Selective antagonists	conivaptan (p*K*_i_ 9.4) [397], tolvaptan (p*K*_i_ 9.4) [2162], satavaptan (p*K*_i_ 8.4–9.3) [24, 383, 384, 1770, 1771, 1913, 1986], lixivaptan (Inverse agonist) (p*K*_i_ 8.9–9.2) [33, 1771], d(CH_2_)_5_[D‐Ile^2^,Ile^4^]AVP (p*K*_i_ 6.9–8.4) [1771], mozavaptan (Inverse agonist) (p*K*_i_ 7.4–8.1) [384, 1771, 1913, 1946, 2162, 2163]	SSR126768A (p*K*_i_ 8.8–9.1) [1772], desGlyNH_2_‐d(CH_2_)_5_[Tyr(Me)^2^,Thr^4^,Orn^8^]OT (p*K*_i_ 8.5), L‐372662 (p*K*_i_ 8.4) [127]
Labelled ligands	[^3^ H]AVP (human, mouse, rat) (Agonist) [334, 383, 384, 1418, 1702, 1913, 1914, 1946, 1986, 2162], [^3^ H]dDAVP (Agonist) [334, 384, 1946], [^3^ H]desGly‐NH_2_[D‐Ile^2^,Ile^4^]VP (p*K*_d_ 8.6)	[^125^ I]d(CH_2_)_5_[Tyr(Me)^2^,Thr^4^,Orn^8^,Tyr‐NH_2_^9^]OVT (Antagonist) (p*K*_d_ 10), [^3^ H]OT (human, mouse, rat) (Agonist) [334, 583, 895, 998], [^111^In]DOTA‐dLVT (p*K*_d_ 8.3) [333]

### Comments

The V_2_ receptor exhibits marked species differences, such that many ligands (d(CH_2_)_5_[D‐Ile^2^,Ile^4^]AVP and [^3^ H]desGly‐NH_2_[D‐Ile^2^,Ile^4^]VP) exhibit low affinity at human V_2_ receptors [29]. Similarly, [^3^ H]d[D‐Arg ^8^]VP is V_2_ selective in the rat, not in the human [1702]. The gene encoding the V_2_ receptor is polymorphic in man, underlying nephrogenic diabetes insipidus [152]. D[Cha ^4^]AVP is selective only for the human and bovine V_1B_ receptors [439], while d[Leu ^4^]LVP has high affinity for the rat V_1B_ receptor [1553].

### Further reading on Vasopressin and oxytocin receptors

Bartz JA et al. (2011) Social effects of oxytocin in humans: context and person matter. Trends Cogn. Sci. (Regul. Ed.) 15: 301‐9 [PMID:21696997]

Knepper MA. (2012) Systems biology in physiology: the vasopressin signaling network in kidney. Am. J. Physiol., Cell Physiol. 303: C1115‐24 [PMID:22932685]

Koshimizu TA et al. (2012) Vasopressin V1a and V1b receptors: from molecules to physiological systems. Physiol. Rev. 92: 1813‐64 [PMID:23073632]

Manning M et al. (2012) Oxytocin and vasopressin agonists and antagonists as research tools and potential therapeutics. J. Neuroendocrinol. 24: 609‐28 [PMID:22375852]

Meyer‐Lindenberg A et al. (2011) Oxytocin and vasopressin in the human brain: social neuropeptides for translational medicine. Nat. Rev. Neurosci. 12: 524‐38 [PMID:21852800]

Neumann ID et al. (2012) Balance of brain oxytocin and vasopressin: implications for anxiety, depression, and social behaviors. Trends Neurosci. 35: 649‐59 [PMID:22974560]

## VIP and PACAP receptors

### Overview

Vasoactive intestinal peptide (VIP) and pituitary adenylate cyclase‐activating peptide (PACAP) receptors (nomenclature as agreed by the NC‐IUPHAR Subcommittee on Vasoactive Intestinal Peptide Receptors [739, 740]) are activated by the endogenous peptides VIP(VIP, P01282), PACAP‐38(ADCYAP1, P18509), PACAP‐27(ADCYAP1, P18509), peptide histidine isoleucineamide (PHI {Mouse, Rat}), peptide histidine methionineamide (PHM(VIP, P01282)) and peptide histidine valine (PHV(VIP, P01282)). VPAC_1_ and VPAC_2_ receptors display comparable affinity for the PACAP peptides, PACAP‐27(ADCYAP1, P18509) and PACAP‐38(ADCYAP1, P18509), and VIP(VIP, P01282), whereas PACAP‐27(ADCYAP1, P18509) and PACAP‐38(ADCYAP1, P18509) are >100 fold more potent than VIP(VIP, P01282) as agonists of most isoforms of the PAC_1_ receptor. However, one splice variant of the human PAC_1_ receptor has been reported to respond to PACAP‐38(ADCYAP1, P18509), PACAP‐27(ADCYAP1, P18509) and VIP(VIP, P01282) with comparable affinity [411]. PG 99‐465[1374] has been used as a selective VPAC_2_receptor antagonist in a number of physiological studies, but has been reported to have significant activity at VPAC_1_ and PAC_1_ receptors [446]. The selective PAC_1_ receptor agonist maxadilan, was extracted from the salivary glands of sand flies (Lutzomyia longipalpis) and has no sequence homology to VIP(VIP, P01282) or the PACAP peptides [1383]. Two deletion variants of maxadilan, M65[1994] and Max.d.4[1384] have been reported to be PAC_1_ receptor antagonists, but these peptides have not been extensively characterised.

**Table:** 

Nomenclature	PAC_1_ receptor	VPAC_1_ receptor	VPAC_2_ receptor
HGNC, UniProt	ADCYAP1R1, P41586	VIPR1, P32241	VIPR2, P41587
Potency order of endogenous ligands	PACAP‐27 (ADCYAP1, P18509), PACAP‐38 (ADCYAP1, P18509) ≫VIP (VIP, P01282)	VIP (VIP, P01282), PACAP‐27 (ADCYAP1, P18509), PACAP‐38 (ADCYAP1, P18509) ≫GHRH (GHRH, P01286), PHI {Pig}, secretin (SCT, P09683)	VIP (VIP, P01282), PACAP‐38 (ADCYAP1, P18509), PACAP‐27 (ADCYAP1, P18509) >PHI {Pig} ≫GHRH (GHRH, P01286), secretin (SCT, P09683)
Selective agonists	maxadilan [446], maxadilan [446]	[Lys^15^,Arg^16^,Leu^27^]VIP‐(1‐7)/GRF‐(8‐27)‐NH_2_ [1369], [Ala ^11,22,28^]VIP [1458]	Ro 25‐1553 [669, 930, 1369], Ro 25‐1392 [2144]
Selective antagonists	–	PG 97‐269 (pIC_50_ 8.7) [668, 930]	–
Labelled ligands	[^125^ I]PACAP‐27 (Agonist) [1581]	[^125^ I]VIP (human, mouse, rat) (Agonist) [1458], [^125^ I]PACAP‐27 (Agonist)	[^125^ I]VIP (human, mouse, rat) (Agonist) [1458], [^125^ I]PACAP‐27 (Agonist)

### Comments

Subtypes of PAC_1_ receptors have been proposed based on tissue differences in the potencies of PACAP‐27(ADCYAP1, P18509) and PACAP‐38(ADCYAP1, P18509); these might result from differences in G protein coupling and second messenger mechanisms [2018], or from alternative splicing of PAC_1_ receptor mRNA [1859].

### Further reading on VIP and PACAP receptors

Harmar AJ et al. (1998) International Union of Pharmacology. XVIII. Nomenclature of receptors for vasoactive intestinal peptide and pituitary adenylate cyclase‐activating polypeptide. Pharmacol Rev 50: 265‐270 [PMID:9647867]

Harmar AJ et al. (2012) Pharmacology and functions of receptors for vasoactive intestinal peptide and pituitary adenylate cyclase‐activating polypeptide: IUPHAR review 1. Br. J. Pharmacol. 166: 4‐17 [PMID:22289055]

Reglodi D et al. (2012) Effects of pituitary adenylate cyclase activating polypeptide in the urinary system, with special emphasis on its protective effects in the kidney. Neuropeptides 46: 61‐70 [PMID:21621841]

Smith CB et al. (2012) Is PACAP the major neurotransmitter for stress transduction at the adrenomedullary synapse? J. Mol. Neurosci. 48: 403‐12 [PMID:22610912]
